# ChatMOF: an artificial intelligence system for predicting and generating metal-organic frameworks using large language models

**DOI:** 10.1038/s41467-024-48998-4

**Published:** 2024-06-03

**Authors:** Yeonghun Kang, Jihan Kim

**Affiliations:** Department of Chemical and Biomolecular Engineering, Korea Advanced Institute of Science and Technology (KAIST), 291, Daehak-ro, Yuseong-gu, Daejeon, Republic of Korea

**Keywords:** Computational methods, Structure prediction, Metal-organic frameworks

## Abstract

ChatMOF is an artificial intelligence (AI) system that is built to predict and generate metal-organic frameworks (MOFs). By leveraging a large-scale language model (GPT-4, GPT-3.5-turbo, and GPT-3.5-turbo-16k), ChatMOF extracts key details from textual inputs and delivers appropriate responses, thus eliminating the necessity for rigid and formal structured queries. The system is comprised of three core components (i.e., an agent, a toolkit, and an evaluator) and it forms a robust pipeline that manages a variety of tasks, including data retrieval, property prediction, and structure generations. ChatMOF shows high accuracy rates of 96.9% for searching, 95.7% for predicting, and 87.5% for generating tasks with GPT-4. Additionally, it successfully creates materials with user-desired properties from natural language. The study further explores the merits and constraints of utilizing large language models (LLMs) in combination with database and machine learning in material sciences and showcases its transformative potential for future advancements.

## Abstract (web summary)

LLMs can be augmented with tools to increase their capabilities. Here, authors have developed an artificial intelligence system called ChatMOF combining LLMs and specialised libraries and utilities to predict and generate metal-organic frameworks.

## Introduction

The realm of generative artificial intelligence (AI) is witnessing an unprecedented surge, predominantly fostered by a new generation of computational tools known as large-scale language models (LLMs)^1–5^. These innovative models are deeply rooted in a novel architectural design paradigm, referred to as transformer models^6^. Their capabilities, however, stretch far beyond the domain of basic language tasks. These models are programmed to perform tasks that mimic certain aspects of human cognition, such as processing and applying new information based on few or no examples (few-shot and zero-shot learning)^3,5,7^. This is achieved through the understanding of vast volumes of text data, underscoring the immense potential held by these models. A significant advancement in this swiftly changing domain is the rise of autonomous LLM agents that employ LLMs for diverse functions^8^ (https://github.com/yoheinakajima/babyagi; https://github.com/Significant-Gravitas/Auto-GPT). The application of LLMs for analyzing information, extracting pertinent details, and creating responses is becoming increasingly popular in several research fields, owing to their capacity for independent operation^9–12^. They harness the capabilities of LLMs using prompt engineering^13–15^, fine-tuned them^16–18^, or meld them with other scientific tools^8,19–21^. These systems are highly effective in autonomously processing data and producing outcomes without the need for human involvement.

Despite marked progress in application of LLM across diverse fields such as chemistry^20,22–26^, medicine^27,28^, and biology^29,30^, the full potential of its advanced technology within materials science remains largely untapped. This limitation primarily stems from two considerable challenges. Firstly, the inherent complexity of these advanced materials, such as zeolite, porous polymer networks, covalent-organic framework, and metal-organic framework, poses a significant hurdle. These materials often lack suitable text-compatible input representations, which hinders the ability to fully capture their intricate properties^31–34^. This difficulty in encoding materials for LLMs restricts their understanding and processing capabilities. Secondly, there is a notable scarcity of material-specific training data in the field. In comparison to other disciplines, materials science lags behind due to fewer dedicated databases and their associated data, exacerbating the challenge of representing this scant data in a text format suitable for LLMs. Despite these obstacles, there are ongoing attempts to leverage the capabilities of LLMs in materials science^15,18,22^. However, so far, these efforts have primarily focused on extracting data from scientific literature and generating responses based on this extracted data, with the actual material itself remaining a largely untouched resource. As such, the exploration and realization of the full potential of LLMs within the sphere of materials science still beckons.

In this work, we highlight the development of a methodology that employs AI systems to automate the generation of new materials and the prediction of their properties, focusing specifically on metal-organic frameworks (MOFs)^35–37^. MOFs are used in many chemical applications^38–41^ due to their large porosity^42–44^, high surface area^43^, and exceptional tunability^45^. To this end, we have developed the artificial intelligence system for MOFs (called ChatMOF), which holds the potential to predict MOF properties from text-based inquiries and to generate MOFs with specified properties (i.e., inverse design). Recently, there has been a rising interest in combining LLMs with traditional machine learning models in the materials field^46^, and this pioneering approach can potentially significantly bridge the gap between the novice users and the computational and machine learning tools, which can potentially facilitate the progress in developing new materials for various applications.

## Results

### Design of ChatMOF

**Fig. 1:**
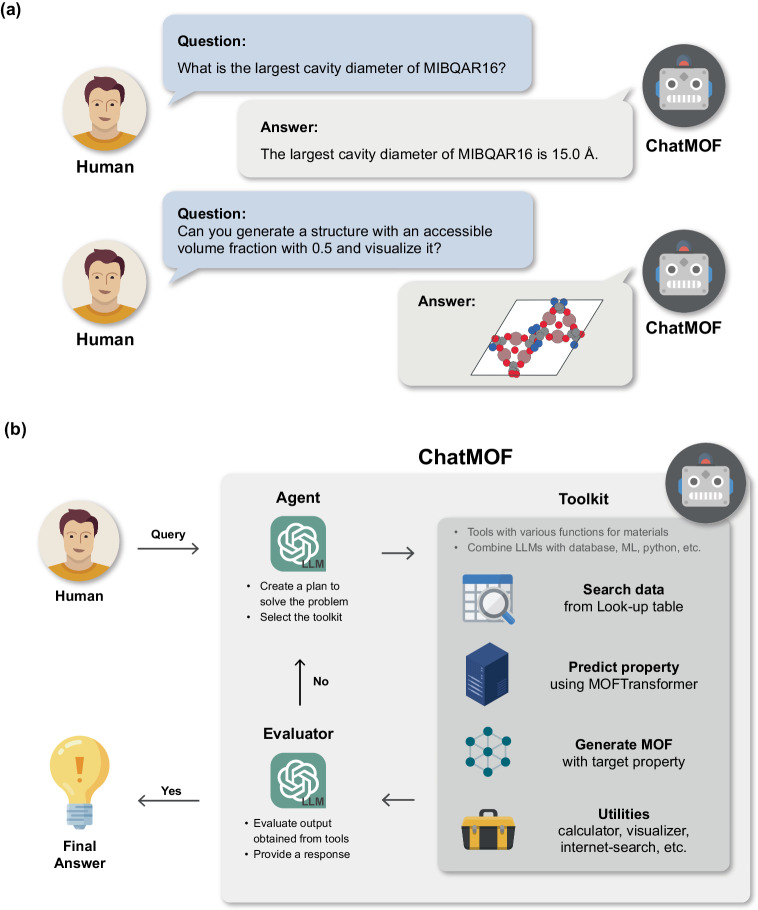
Conceptual and Schematic Representation of ChatMOF. **a** A Conceptual image that explains the ChatMOF. When a user poses a textual question about the properties of a MOF, an appropriate answer is provided by ChatMOF. If a user desires to generate a new MOF, ChatMOF is capable of creating a new MOF that satisfies the condition. **b** The schematic image of ChatMOF. ChatMOF comprises three core components: an agent, toolkit, and an evaluator. Upon receiving a query from human, the agent formulates a plan and selects a suitable toolkit. Subsequently, the toolkit generates outputs following the proposed plan, and the evaluator makes these results into a final response.

The effectiveness of autonomous LLM agents is predicated on its capability to accurately extract essential details from textual inputs and offer relevant responses, irrespective of the presence of a rigidly structured query^22^. This concept is clearly illustrated in ChatMOF, as demonstrated in Fig. 1a. A user may pose a query in textual form regarding the properties of a material, to which ChatMOF responds by supplying a detailed description related to the material in question. Moreover, the operational scope of this system extends beyond the simple retrieval of information. When a user expresses the need to generate a MOF with specific properties, ChatMOF is capable of generating the requested material structure accordingly.

In the context of ChatMOF, LLM functions as central coordinators, managing and evaluating processes, similar to how a central processing unit (CPU) operates in computing. While the LLM excels in general reasoning tasks, its performance in specialized areas is less robust^20^. Supplementary Fig. S1 illustrates this by presenting a direct MOF-related question to the LLM, highlighting the constraints faced when engaging an LLM in specialized tasks. Nonetheless, LLM demonstrates remarkable proficiency in assimilating and leveraging diverse databases and machine learning models. This strength originates from their inherent reasoning abilities and fluid processing capabilities^47,48^. ChatMOF uses the LLM to systematically organize and apply various tools for information gathering, similar to a well-executed algorithm in computer programming. This synergy allows the system to precisely predict material properties, retrieve synthesis methods from a text-mined database, and fabricate new materials with preset properties.

As depicted in Fig. 1b, ChatMOF is composed of three main components: an agent, toolkit, and an evaluator. The agent processes human queries through four primary operational stages (i.e., data analysis, action determination, input management, and result observation), following the methodology outlined in the ReAct^49^ and MRKL papers^50^. Initially, the user’s query is established as the objective, followed by systematic planning to determine the steps to meet this objective. Subsequently, ChatMOF decides on the appropriate tool to employ from the available options. After the chosen tool is executed, the observed results serve as the basis for evaluating whether a final answer can be generated. If feasible, the final answer is presented, otherwise, the process cycles back to the thought step to formulate a new strategy.

### Toolkit

ChatMOF employs an assortment of tools to acquire, predict, or generate material information. These tools can be primarily classified into four categories: table-searcher, internet-searcher, predictor, generator, and utilities. Table-searcher involves obtaining desired information from existing data. The predictor utilizes machine learning models to obtain specified properties. The generator refers to the tool that constructs material structures fulfilling certain properties. Lastly, the utilities encompass a variety of aids like calculators, file saving and reading functions, visualizer, and internet-searcher.

Due to the facile synthesis MOF structures, there are many different database associated with the MOF structures: (1) computational-ready experimental MOFs (CoREMOF)^51,52^ and (2) quantum MOF (QMOF) database^53^. The CoREMOF database is an archive of synthesized materials present in a CSD MOF subset^54^, encompassing computations of various properties of MOFs including geometric descriptors. The QMOF database is populated with electrical property data, such as band gap, formation energy, HOMO, and LUMO, derived through DFT calculations. When a user demands these electrical properties, ChatMOF seeks and extracts them from the QMOF database.

**Fig. 2:**
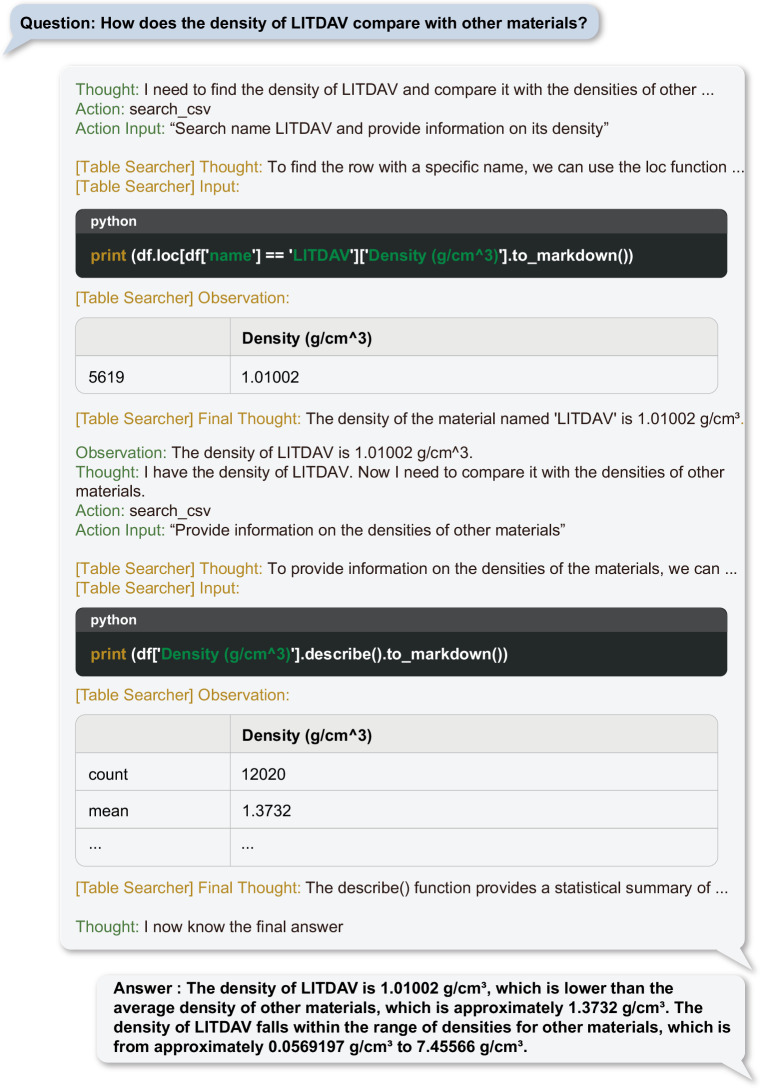
Example of a Table Searcher for the question “How does the density of LITDAV compare to other materials”. The human’s question prompts the system to devise a strategy. From this, Python code is generated and executed to extract the desired information, which is then returned as the final answer. Tested on November 18, 2023 at 17:41 KST with GPT-4.

As such, if a user seeks information about a specific MOF that is included in these databases, ChatMOF can locate and deliver the desired information from the pre-tabulated data. Figure 2 provides an illustrative example of a table-search operation conducted by ChatMOF. In response to a user query, ChatMOF automatically determines the optimal method to extract the necessary data. Subsequently, it creates a Python code tailored to retrieve specific information from the database in accordance with the premeditated strategy. This code typically uses the “pandas^55^” library to extract or filter relevant details. The drafted code is then executed within the ChatMOF’s designated executor. After processing the results of this operation, ChatMOF automatically determines the subsequent procedures needed to produce the final answer, which is then delivered as the requested response.

The versatility of ChatMOF extends to handling diverse table data derived from text mining or rule-based coding processes. For questions related to the building blocks of a MOF, the MOFkey^31^ database proves to be instrumental. This particular database leverages rule-based methods to obtain insights about the organic linkers and metal clusters of a MOF, providing details about its topology and the potential presence or absence of interpenetration. In addition, for users seeking guidance on MOF synthesis, the DigiMOF^56^ database becomes a valuable resource. DigiMOF provides an array of synthesis conditions, extracted via text mining techniques from MOF-related academic papers, and includes information on organic and metal precursors, and solvent.

The accuracy of the look-up table search is contingent on the pre-calculated values available in the specific files. And for queries regarding the properties of MOFs that are not available, computational simulation can serve as an attractive alternative method, but unfortunately, simulations are a time-intensive process and an abundance of computational resources^57^. The best resolution to such challenges is the application of machine learning models, which enable high-accuracy predictions grounded in extensive data. In the case of pre-trained machine learning models, predictions are quick and can be made for a significant volume of substances simultaneously, making it an excellent toolkit for integration into ChatMOF.

As an appropriate tool for the prediction task, ChatMOF uses the MOFTransformer^58,59^ model that has been developed in our group for the universal prediction of MOF properties. This model leverages both local features, such as atoms and bonds, and global features like surface area and topology. Having undergone pre-training with one million hypothetical MOFs and subsequent fine-tuning for specific properties, MOFTransformer shows high performance in predicting various properties. Moreover, it affords the convenience of predicting the desired property by retaining the model structure while altering the weights of a model that’s been fine-tuned for the desired property.

**Fig. 3:**
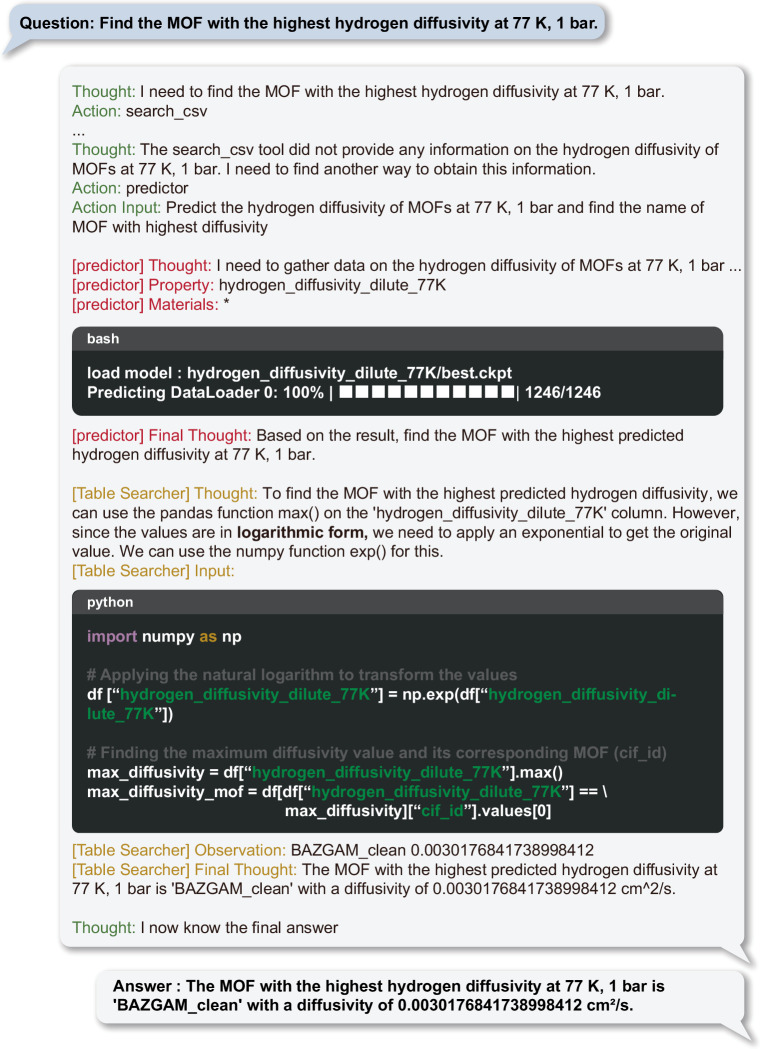
Example of a predictor for the question “Find the MOF with the highest hydrogen diffusivity at 77 K, 1 bar”. The predictor sets up a plan to solve the question, an appropriate model, and target material. Based on this, it uses machine learning to predict the value, which is then used to derive the final answer. Tested on November 20, 2023 at 10:59 KST with GPT-4.

The key to generating accurate responses is selecting the appropriate fine-tuned model with the MOFTransformer and the material to which it will be applied, based on the query. Similar to HuggingGPT^8^, ChatMOF does not directly compute the material properties, but it rather selects the appropriate machine learning model. Figure 3 shows the example prompt for ChatMOF using the MOFTransformer predictor. When a user requests information like “Identify the MOF with the highest hydrogen diffusivity at 77 K, 1 bar,” the agent develops a strategy to address the query. It chooses the most suitable fine-tuned MOF transformer model, in this instance, “hydrogen_diffusivity_dilute_77K,” tailored to the task. The agent then identifies the materials for prediction (here, all materials) and utilizes the selected model for making predictions. Subsequently, we conduct a table search to identify the substance with the highest value, as determined by the machine learning model. This process takes into account specific aspects of the fine-tuning model, including units, conditions, and the logarithmic scale. Since the hydrogen diffusivity model outputs logarithmic values, we execute code to identify the substance with the highest value, converting these values back to their exponential form to obtain the final result.

Finally, a key aspiration among researchers in the field of MOFs is the inverse design of MOFs exhibiting desired properties. In materials science, various generative models, including Generative Adversarial Networks (GAN)^60,61^ and Diffusion models^62^, have been employed for inverse design. However, due to the inherent complexity of MOFs, which includes a large number of atoms, large void fraction, and complex topologies, an atom-by-atom inverse design approach has been elusive. As a workaround, MOF inverse design has been facilitated top-down approaches leveraging through genetic algorithms^63–65^, Variational Autoencoders^66^ (VAE), or reinforcement learning^67^ for the selection of building blocks and their placement into suitable topologies.

Genetic algorithms are notably suitable for integration with the LLM. As a bio-inspired optimization methodology, genetic algorithms operate on a selection, mutation, and crossover principle, making them adaptable and efficient^68^. For their application to MOFs, these frameworks must be delineated by genes comprising topology and building blocks. For instance, a representative MOF, HKUST-1, can be depicted as tbo+N17 + N10, with tbo representing topology and N17 and N10 representing the building block notations. As these gene representations are textual, they facilitate the application of genetic algorithms using an LLM.

**Fig. 4:**
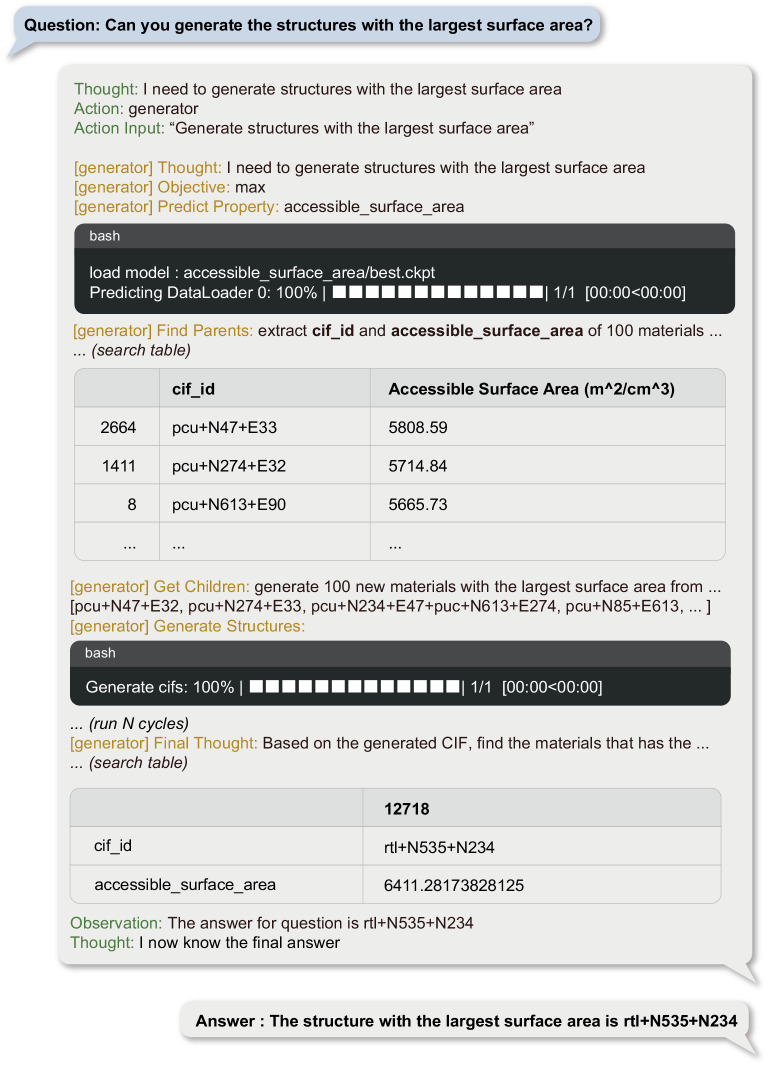
Example of a generator for the question “Can you generate the structures with the largest surface area”. The generator establishes a plan, objective, and property for the human question. Based on this, it finds parents that satisfy the objective. It uses a genetic algorithm to create children genes and generate structures. This is repeated for a number of cycles to generate new MOFs, which are used to derive the final answer. The full process of the example is provided in Supplementary Note S1 and Supplementary Data 1. Tested on October 13, 2023 at 14:26 KST with GPT-4.

Figure 4 showcases the utilization of a genetic algorithm by ChatMOF to fabricate a MOF per user specifications. Upon receiving a user query, the system formulates a strategy, optimized through algorithmic processes, based on genetic algorithms. It also identifies the target property and determines the loss function most suited for the objective, such as choosing the maximum, minimum, or closest value. Following the algorithmic strategy, ChatMOF algorithmically selects parent genes from the existing database, aligning with the predefined loss function. In the genetic algorithm employed, parent genes demonstrating high potential based on the desired target characteristics are selected, enhancing the probability that the resultant child gene will exhibit the targeted property more prominently. These children are then transformed into a structure file, and their properties are estimated through machine learning. This procedure is reiterated a fixed number of times, generating multiple generations of children with each generation yielding MOFs progressively nearer to the target. From the created structures, the one that aligns most closely with the question is finally chosen and presented as the response.

Moreover, ChatMOF is engineered to perform a diverse set of utilities, which extend beyond the realms of LLM. This includes capabilities such as file search, internet search, and even simple calculations. These additional functionalities are primarily enabled by leveraging the varied capabilities provided by LangChain (https://github.com/hwchase17/langchain), enhancing the overall functionality and utility of ChatMOF. Additionally, the development of unit-conversion and visualization tools has broadened ChatMOF’s range of capabilities. Thus, it is not merely a material analysis tool, but a comprehensive system that can accommodate a wide array of tasks and operations.

Furthermore, ChatMOF incorporates the Atomic Simulation Environment (ASE)^69^ library as an integral tool to facilitate diverse operations on material structure data. The ASE library holds considerable importance in the field of materials science due to its capabilities, including atom manipulation, cell information acquisition, and visualization, among others. Similar to the function of a table searcher, when confronted with a query, ChatMOF devises a strategic plan and constructs suitable Python code utilizing the ASE library to fulfil the query’s demands. Subsequently, this code is executed.

**Fig. 5:**
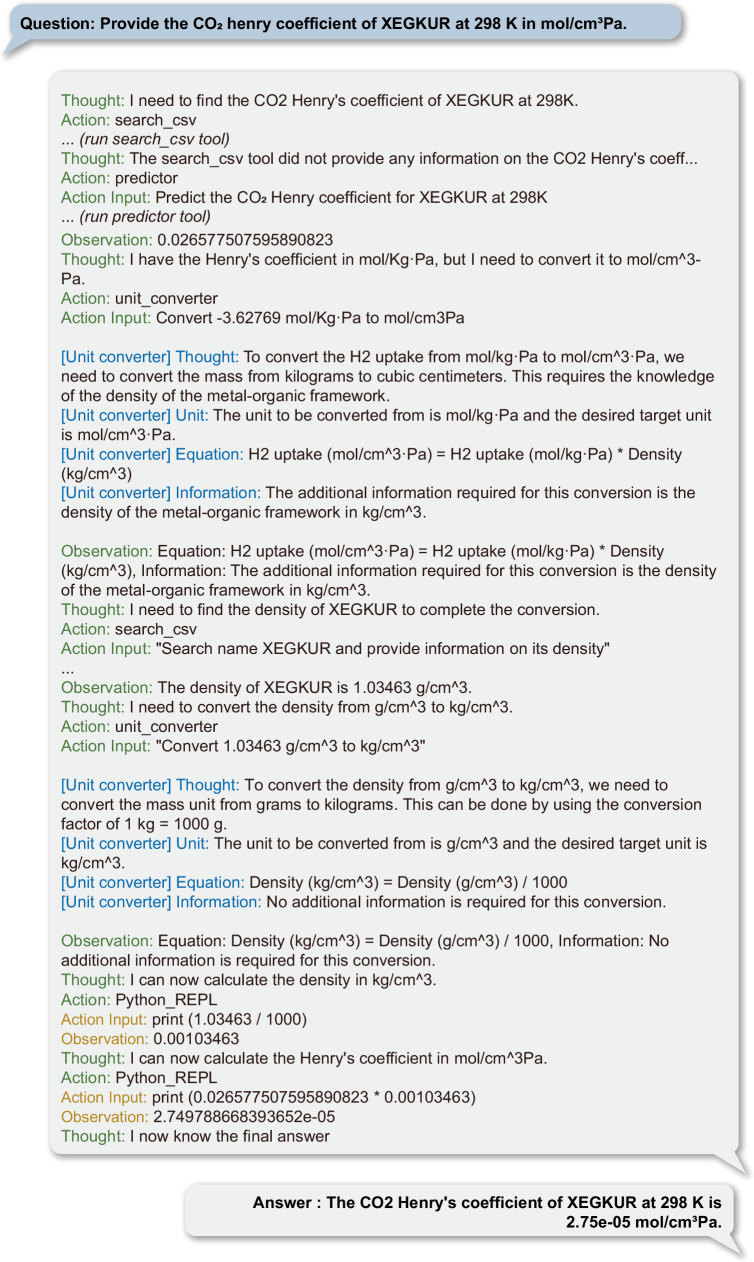
Example of a complex and multi-step question “Provide the CO_2_ Henry coefficient of XEGKUR at 298 K in mol/cm^3^Pa”. ChatMOF uses 4 different tools (predictor, unit-converter, table searcher, python repl) to solve the problem. The full process of the example is provided in Supplementary Data 1. Tested on November 25, 2023 at 18:23 KST with GPT-4.

With these capabilities, ChatMOF is programmed to efficiently process intricate and multi-step tasks. As depicted in Fig. 5, ChatMOF efficiently responds to the query “Provide the CO_2_ Henry coefficient of XEGKUR at 298 K in mol/cm^3^Pa”. Initially, ChatMOF employs a predictor to ascertain the Henry coefficient of CO_2_. It then employs a unit conversion tool to perform the conversion from mol/KgPa into mol/cm^3^Pa units. In this conversion process, ChatMOF identifies the requirement for additional data about the density of XEGKUR. It then conducts a table search to obtain this necessary Density value. With the density figure in hand, ChatMOF applies the unit conversion tool to transform g/cm^3^ to kg/cm^3^, ultimately synthesizing all this information to arrive at the final answer.

Further illustrating ChatMOF’s capabilities, Supplementary Figs. S4 and S5 display its approach in addressing complex, non-intuitive problems that are challenging to translate into structured data. When processing complex inquiries, such as “What is the relationship between the amount of H_2_ absorption and accessible volume in a MOF ChatMOF identifies the core issue—the interplay between these two properties—and determines the most effective approach to generate an accurate response. Additionally, leveraging its material domain expertise, the LLM calculates the absorptions of O_2_ and N_2_, subsequently deriving the selectivity ratio, to answer questions like “Find the O_2_/N_2_ selectivity of XEGKUR at 298 K, 1 bar” without requiring extra information. These scenarios highlight ChatMOF’s proficiency not just in translating natural language into structured data, but in interpreting and strategically processing complex queries to develop a tailored and efficient resolution plan.

### Evaluation

To evaluate performance of ChatMOF, analysis was conducted for “search task”, “prediction task”, and “generation task”. For evaluation purposes, questions for ChatMOF were created utilizing GPT-4 to generate various sentences about the given properties of a MOF. This approach of utilizing GPT-4 for question generation aimed to minimize author involvement, increase measurement fairness, and generate a variety of questions. The prompts used to elicit these questions are displayed in Supplementary Note S2 and S3. The respective questions for each task can be found in Supplementary Tables S1–3. In this investigation, the accuracy measure ensures that the AI’s process of reasoning, from devising a plan to tackle the problem to choosing the toolkit, and then deciphering the results, remains untainted by logical discrepancies. This is because ChatMOF is intended to imitate the expert’s systematic approach to problem-solving.

The accuracy analysis of ChatMOF involved the use of three labels: “True”, “False (token limit exceeded)”, and “False (logic error)”. The “True” label indicates that ChatMOF’s processes correctly matched the logic required to produce an accurate answer. The term “False (Token Limit Exceeded)” was used when the token count in LLM surpassed the maximum allowance of 4000, thus obstructing further progress. Lastly, the “False (Logic Error)” label designated situations where an error in ChatMOF’s logic resulted in an incorrect response or an anomaly. Such situations typically arise due to the formulation of a flawed procedure for obtaining an answer or due to an error in interpreting the output, causing the system to deviate from the intended outcome. When encountering the ‘token limit exceeded’ error, it arises within the LLM, causing the process to halt without further progress. This error mainly influences the recall value because it halts the analysis once the token limit is exceeded. As for the ‘logic error’, it stems from the reasoning abilities of the LLM. This leads to incorrect responses, which in turn affect the precision value. This error type directly challenges the LLM’s capability to provide accurate and logical answers.

**Fig. 6:**
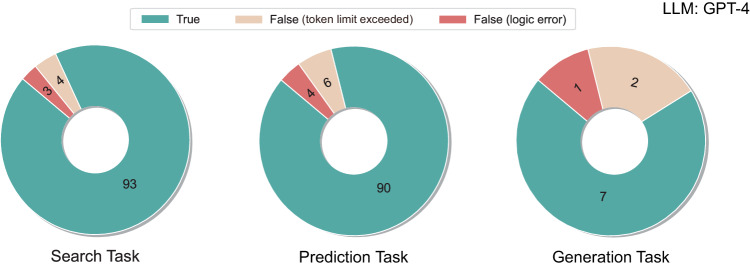
Depiction of accuracies for three tasks using GPT-4 model—search, prediction, and generation (tested on 23.08.10 to 23.08.14). Accuracies were evaluated based on three labels: True, False (token limit exceeded), and False (logic error). False (token limit exceeded) indicates that the LLM has exceeded the maximum token allowance, while False (Logic Error) indicates that the ChatMOF’s logic has resulted in an incorrect response or anomaly. The number in the bar represents the count of each label.

Figure 6 presents the accuracy measurements for the three tasks using ChatMOF with GPT-4. Accuracy was measured for 100 sample questions for the search and prediction tasks, and 10 sample questions for the generation task. The number in the bar graph indicates the number of questions in each class. Both the search and prediction tasks rendered accurate answers with high frequency. Excluding ‘Token Limit Exceeded’ instances (4 out of 100, 6 out of 100, and 2 out of 100, for search, prediction, and generation tasks respectively), they exhibit high accuracies of 96.9% and 95.7%, respectively. For the generation task, the accuracy stood at 87.5%. Due to the inherent complexity of this task, compared to the other two, the accuracy rate observed is lower. Regardless, all three tasks report high accuracy rates, and these tasks carry significant weight because these questions cannot be effectively answered by directly querying LLMs. (Refer to Supplementary Fig. 1). This is because LLM often fall short in providing precise information due to their lack of detailed material-specific data, particularly in the case of properties that are challenging to ascertain via internet searches.

Also ChatMOF, when integrated with GPT-4, exhibits superior performance compared to its integration with GPT-3.5-turbo. As evidenced in Supplementary Fig. S6, the accuracy of ChatMOF with GPT-3.5-turbo stands at 95%, 91%, and 77.8% for the search, prediction, and generation tasks respectively, excluding instances of “Token Limit Exceeded”. Across all tasks, GPT-4 consistently outperforms GPT-3.5-turbo in accuracy. The enhanced accuracy of GPT-4 can be attributed to its improved algorithms and processing capabilities, particularly during the planning phase. Supplementary Fig. S7 illustrates the distinct approaches that GPT-4 and GPT-3.5-turbo take when presented with the same query: “How does the pore limiting diameter of YUSGID_clean compare with other materials?”. While GPT-3.5-turbo processes values for all materials mentioned in the query, leading to a token error and subsequent inability to provide an answer, GPT-4 employs a more comprehensive algorithm. The algorithm analyzes the distribution of all materials, using metrics such as mean, variance, and quartile values of the property in question. This approach enables GPT-4 to determine the relative position of the target material in the overall distribution, thus delivering a more informative response to the user.

It is worth noting that the primary cause of encountering token limit errors is influenced by LLM’s coding proficiency rather than the specific maximum token count utilized. Supplementary Fig. S8 illustrates the accuracies of employing GPT-3.5-turbo-16k (with a maximum token limit of 16,835), which boasts a greater maximum token limit compared to GPT-3.5-turbo (with a maximum tokens limit of 4097). Despite the augmented maximum token capacity, the frequency of surpassing this limit has not decreased. This phenomenon arises from suboptimal code execution, as indicated in the comparison with GPT-4 (see Supplementary Fig. S7). These results print the entire table and greatly exceed the typical maximum token threshold, thereby leading to token limit errors. Consequently, it becomes imperative to enhance the performance of the LLM itself, rather than merely expanding the token count, in order to improve overall performance.

For the “search task,” the writing of code utilizing the pandas library significantly impacts the accuracy. ‘Token Limit Exceeded’ generally occurs when the output code surpasses the permissible token count. This frequently arises when all relevant materials that satisfy a given condition are provided (for example, when a list of materials with a particular property is listed), or when the question contains a comparative clause such as “compared to other materials.” ‘Logic Error’ typically appears due to an incorrect algorithmic approach or a code error. An instance of this would be when a request to provide 10 specific items is met with a misguided strategy that solely aims to “extract high values,” failing to retrieve the specified number of items.

During the “prediction task,” difficulties often occur in the interpretation process of the observed values using machine learning techniques. Both the ‘Token Limit Exceeded’ and ‘Logic Error’ occurrences can stem from the effort to draw the correct answer from the table based on the estimated values. ‘Logic Errors’ can manifest not only during the table search phase but also during the strategy formulation stage. An incorrect algorithm could either cause the loading of an unsuitable model or generate an input that is incompatible with the intended model.

### Inverse design validation

The inverse design process is bifurcated into two stages: planning for a genetic algorithm and executing it. Predominantly, errors in inverse design surface during the planning stage. In this phase, the goal and properties of the genetic algorithm are defined, and code is devised for selecting parental genes for use in the genetic algorithm. As indicated in Fig. 6 and Supplementary Figs. S6 and S8, ChatMOF demonstrates considerable accuracy (7 out 10) in this aspect of inverse design. The primary errors during planning are encountered in the selection of parental genes. These errors typically arise when a parent gene is not retrievable from the database. When the objective function targets a maximum or minimum value, an appropriate parent gene is usually identifiable. However, issues occur if the objective function is inaccurately set, leading either to an inability to find any suitable parent genes or to an overwhelming abundance of them. A lack of suitable parents aligning with the set objective can easily result in a logic error, while an excess of such parents may trigger a token limit error.

The execution phase of the genetic algorithm using LLM plays a crucial role in the efficiency of material generation. It is worth noting that the performance of the LLM is critical to the efficiency of the genetic algorithm execution. Supplementary Fig. S9 illustrates the degree of overlap between these children and their parents, as well as the number of children generated by GPT-4 and GPT-3.5-turbo. It is observed that GPT-3.5-turbo tends to generate children that are almost identical to the parents, despite instructions not to duplicate an existing parent. This indicates that GPT-3.5-turbo is not suitable for executing genetic algorithms. On the other hand, GPT-4 shows a much lower overlap percentage of around 30% between parents and children. One issue with the generated children is their inconsistent number. Although they are intended to create 100 new children per topology, typically only about 60 are generated. This inconsistency is due to limits in the model’s token count and GPT’s generation capabilities. When compared to traditional code-based algorithms, which can generate the exact number of children without repetition, LLMs currently need to improve to match this efficiency in genetic algorithms.

**Fig. 7:**
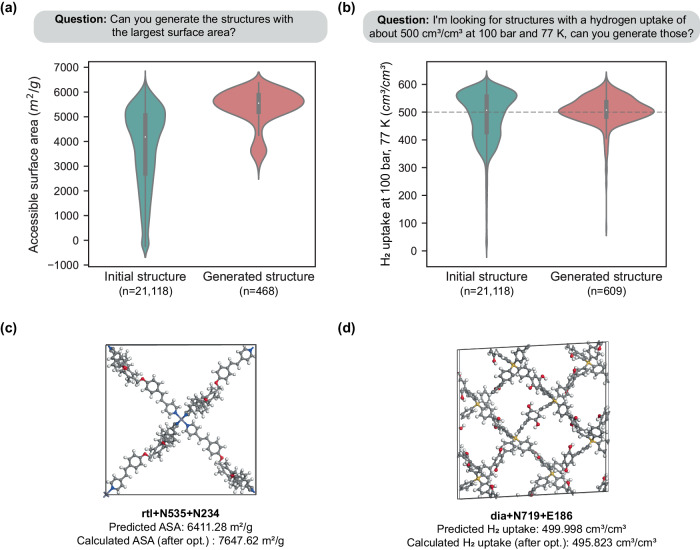
Analysis of MOF inverse design conducted by ChatMOF. **a** Violin plot illustrating the distribution of initial and generated structures for a question related to the maximum surface area value. The inner box represents the interquartile range (IQR) and the white horizontal line inside the box indicates the median of the distribution. The number in parentheses represents the number of data. **b** Violin plot depicting the distributions of initial and generated structures in response to a query about hydrogen uptake values close to 500 cm^3^/cm^3^. The gray horizontal line indicates the location of 500 cm^3^/cm^3^. **c** Illustration of the MOF with the largest surface area as generated by ChatMOF. ASA stand for accessible surface area. **d** Representation of the MOF with an H_2_ uptake value closest to 500 cm^3^/cm^3^ at 298 K, 1 bar, as generated by ChatMOF. The term “opt” stands for the geometric optimization of structures via molecular dynamics simulation. The data used to plot the violin is provided in Source Data 1. Tested on October 13, 2023 at 14:26 KST with GPT-4.

Nevertheless, it is worth noting that ChatMOF has successfully generated materials that meet user requirements. Figure 7 shows the distribution of MOFs generated by the genetic algorithm under two different scenarios. Figure 7a displays the structures generated in response to the question, ‘Can you generate structures with the largest surface area?’ In this case, ChatMOF is configured to interpret the property as accessible surface area, with the setting adjusted to maximize this parameter. The MOFs initially have a wide distribution, averaging 3748 m^2^/g. However, by the third generation, the average peaks at 5554 m^2^/g. Similarly, Fig. 7b illustrates structures that aim for a hydrogen uptake of approximately 500 cm^3^/cm^3^ at 100 bar and 77 K in response to the question, ‘Can you generate structures with a hydrogen uptake of about 500 cm^3^/cm^3^ at 100 bar and 77 K?’ The final generation of the LLM genetic algorithm is narrowly focused around 500 cm^3^/cm^3^, indicating its effectiveness. The initial range was between 250 and 650 cm^3^/cm^3^.

Figures 7c, d depict the final structures for the queries in 7(a) and 7(b). The optimal structure in 7(c), rtl+N535 + N234, boasts the highest surface area amongst the generated MOFs. The predicted value stands at 6411.28 m^2^/g. Upon performing a geometric optimization and calculating accessible surface area using Zeo + +^70^, the surface area is revealed to have 7647.62 m^2^/g. This value is notably higher when compared to the CoREMOF database. Supplementary Fig. S10 illustrates the distribution of accessible surface areas within CoREMOF. This particular structure’s surface area ranks the third-highest position in the CoREMOF ranking. In a similar vein, the optimal configuration of dia+N719 + E186, showcased in Fig. 7d, possesses a predicted H_2_ uptake of 499.998 cm^3^/cm^3^ at 1 bar and 298 K, mirroring the stipulated target of 500 cm^3^/cm^3^. Following geometric optimization of this structure, its uptake was calculated using RASPA, yielding a value strikingly close to the goal, at 495.823 cm^3^/cm^3^.

Despite its successes, the generation task of ChatMOF does present some limitations. Chief among these is the decrease in gene diversity due to constraints on input and output tokens. The token count restricts the number of parent and child structures to around 100, a fraction compared to inversed design studies that employ conventional genetic algorithm procedures that generate upwards of 100,000 structures for each generation. Other constraints, such as the limited number of topologies and cycles, stem from resource and time restrictions. Yet, despite these limitations, ChatMOF’s algorithms effectively generate MOFs that align well with the objective function, demonstrating its operational efficacy.

## Discussion

The investigation into the role of generative AI in materials science, specifically through the lens of ChatMOF, unveils substantial potential for predicting and generating MOFs. This unique system, which bridges the transformative capabilities of AI and the intricate facets of materials science, demonstrates exceptional performance across various tasks. The accuracy analysis reports high success rates, notably 96.9% and 95.7% for the search and prediction tasks, respectively. Meanwhile, the more complex structure generation task, despite its intricacy, yields a notable accuracy rate of 87.5%. These promising results highlight ChatMOF’s effectiveness, even in managing the most demanding tasks. Despite certain limitations, like the reliance on the number of pre-trained weights, ChatMOF represents significant progress towards achieving higher autonomy in AI for materials science. As the technology advances, and with structured improvements in model capacity and data sharing on online platforms, it is possible to further optimize ChatMOF’s performance, facilitating remarkable progress in MOF research.

## Methods

### Prompt engineering

Prompt engineering emerges as a vital strategy within the realm of artificial intelligence to enhance and calibrate language models for bespoke tasks and results^71^. It consists of crafting high-caliber prompts that steer machine learning models towards producing precise outcomes^72^. This task necessitates selecting the apt prompt category, refining their size and architecture, and sequencing them effectively in relation to the task at hand. To channel this innovation into the field of materials science, we developed a suite of prompts for agent and toolkit, all delineated in Supplementary Note S4–11. These prompts are combined with LLM through the LangChain library in the ChatMOF system.

Every prompt derives its structure from insights within the MRKL^50^ and ReAct^49^ papers. Each agent and toolkit operates on a sequence encompassing a question/thought/input/observation/final thought/final answer format for problem resolution, with every tool tailored to deliver a format that caters to its specific function. The prediction tool configures the MOFTransformer model and designates the MOF target for forecasting, utilizing the “property” and “material” format, while the generator configures the inverse-design process that setting the aim, searching the parent gene, and facilitating child gene creation through the object/search look-up table/genetic algorithm format. Importantly, the table search provides both the header and the first five lines from the table, and the predictor and generator are provided with a list of predictable properties to guide the LLM to formulate a response within the prescribed bounds. Each prompt is reinforced by two to three exemplars, affording the LLM multiple attempts to refine its proficiency in generating precise responses.

### Details of ChatMOF

ChatMOF is a code that links the LLM and tools (Machine learning, python-library, database, etc) together and the process of manipulating them to generate the user-desired output. For the roles of agent, evaluator, and toolkit within ChatMOF, various LLMs (GPT-4, GPT-3.5-turbo, llama2-7B-chat, llama-2-13B-chat) are employed. To minimize the influence of existing examples and evaluate the effectiveness of the LLM, the ChatMOF system employs the LLM without any fine-tuning. During the experiments, the temperature parameter was calibrated to 0.1.

The search functionality within ChatMOF utilizes the CoREMOF structure^51^, augmented with geometric characteristics obtained via ZEO++^70^ calculations performed at high accuracy (indicated by the “-ha” flag), and incorporates a hard sphere model with a diameter of 3.31 Å (nitrogen). In instances of code discrepancies, corrections are made up to a threshold of three attempts. The predictor module within ChatMOF leans on MOFTransformer, trained on insights from four academic articles^53,73–75^ cited in the MOFTransformer publication.

The example of ChatMOF was tested with 0.2.0 version, and the accuracy measurement was tested with 0.0.0 version.

### Genetic algorithm

The genetic algorithm was executed by running the LLM with prompts in Supplementary Note S8 and generating structure files with PORMAKE^63^ library. The generative aspect of ChatMOF is structured around three iterative cycles. This generator employs a genetic algorithm across nine unique topologies, namely pcu, dia, acs, rtl, cds, srs, ths, bcu, and fsc. For every topology, a batch of 100 offspring genes arises from a set of 100 parental genes, chosen from a foundational group of 2000 MOFs. Structures are then formulated based on these newly minted genes, followed by value computation via the predictor. This cycle refines the pool of parental genes, and after the designated cycles, an optimized target structure is procured from the cumulative data.

### Simulation details for inverse design

MOF structures were optimized using the Universal Force Field (UFF)^76^ within the Forcite module of Materials Studio^77^, without assigning partial charges to the MOF atoms. The procedure for measuring hydrogen uptake was aligned with the approach used by MOFTransformer^58^. The RASPA^78^ software facilitated the calculations. A united atom model was utilized for H_2_, and the pseudo-Feynman–Hibbs approach^79,80^ described its low-temperature behavior, adjusting Lenard–Jones potentials at 77 K. Aside from H_2_, the UFF force field with Lorentz–Berthelot mixing rules and a 12.8 Å cutoff was applied, with no partial charges assigned to the MOF atoms. Grand Canonical Monte Carlo (GCMC) simulations were conducted at 100 bar and 77 K, spanning 10,000 production cycles after 5000 cycles of equilibration.

### Reporting summary

Further information on research design is available in the Nature Portfolio Reporting Summary linked to this article.

### Supplementary information

Supplementary Information

Peer Review File

Description of Additional Supplementary Files

Supplementary Data 1

Reporting Summary

### Source data

Source Data

## Data Availability

Procedures and answers to questions in LLM-specific ChatMOF (GPT-4, GPT-3.5-turbo, GPT-3.5-turbo-16k, Llama-2-7B-chat-hf, Llama-2-13B-chat-hf) are available in the experimental section of the GitHub repository under https://github.com/Yeonghun1675/ChatMOF.git. The fine-tuned model used in ChatMOF is available at https://figshare.com/articles/dataset/Fine-tuned_MOFTransformer_model/24288919, and the hMOF used in the genetic algorithm is available at https://figshare.com/articles/dataset/Database_for_ChatMOF/24287731. Source data are provided with this paper.

## Code availability

The ChatMOF library is available at GitHub (https://github.com/Yeonghun1675/ChatMOF.git.) ref. ^81^. An online demo is available at https://chatmof-online.streamlit.app/.
