# Supplementary material for: ChatMOF: an artificial intelligence system for predicting and generating metal-organic frameworks using large language models
Source: Nat Commun. 2024 Jun 3;15:4705. doi: 10.1038/s41467-024-48998-4 (PMC11148193; doi:10.1038/s41467-024-48998-4)
Supplement: Supplementary file 1 — Supplementary Information [file 41467_2024_48998_MOESM1_ESM.pdf]

Supplementary information for:

ChatMOF: An Artificial Intelligence System for Predicting and Generating Metal-Organic Frameworks Using Large Language Models

*Yeonghun Kang, Jihan Kim**

Department of Chemical and Biomolecular Engineering, Korea Advanced Institute of Science and Technology (KAIST), 291, Daehak-ro, Yuseong-gu, Daejeon 34141, Republic of Korea

Table of Contents

Supplementary Note S1. Full description for example of generator for question "Can you generate the structures with the largest surface area"
Supplementary Note S2. Question generation prompts for search and prediction tasks
Supplementary Note S3. Question generation prompts for generation tasks
Supplementary Note S4. Prompt for planning in the searcher
Supplementary Note S5. Prompt for planning in the predictor
Supplementary Note S6. Prompt for single data retrieval in predictor
Supplementary Note S7. Prompt for planning in generator
Supplementary Note S8. Prompt for genetic algorithm in generator
Supplementary Note S9. Prompt for planning in agent
Supplementary Note S10. Prompt for planning in unit-converter
Supplementary Note S11. Prompt for planning in ASE repl tool
Supplementary Note S12. Analyzing ChatMOF with LLaMA2
Supplementary Note S13. Collaborative Online Platform
Supplementary Note S14. Fine-tuning agent
Supplementary Note S15. Evaluation of ChatMOF at different temperatures of GPT.
Supplementary Note S16. Prompt for Evaluation Score
Supplementary Note S17. Diversity of questions
Supplementary Figure S1. Responses to MOF-related questions posed directly to GPT-4
Supplementary Figure S2. Example of an internet-search for the question "Can you explain metal-organic framework in one sentence?"
Supplementary Figure S3. Example of an ASE repl tool for the question "Provide the number of atoms and cell length of XEGKUR"
Supplementary Figure S4. Example of a complex and multi-step question "What is the relationship between H2 uptake and accessible volume in MOFs?"
Supplementary Figure S5. Example of a complex and multi-step question "Find the O2/N2 selectivity of XEGKUR at 298K, 1bar"
Supplementary Figure S6. Depiction of accuracies for three tasks using GPT-3.5-turbo model - search, prediction, and generation
Supplementary Figure S7. ChatMOF's answer to the question "How does the pore limiting diameter of YUSGID_clean compare with other materials?" using GPT-4 and GPT-3.5-turbo
Supplementary Figure S8. Depiction of accuracies for three tasks using GPT-3.5-turbo-16k model - search, prediction, and generation.
Supplementary Figure S9. Evaluation of GPT-4 and GPT-3.5-turbo's genetic algorithm performance in generating structures with the largest accessible surface area.

Supplementary Figure S10. Violin plot illustrating the distribution of CoREMOF structure for a accessible surface area (m2/g)
Supplementary Figure S11. Illustration of ChatMOF-online platform
Supplementary Figure S12. Example of ChatMOF's answer to the question “What is the CH4 uptake of MIBQAR16 at 298K and 1bar”
Supplementary Figure S13. ChatMOF's response with fine-tuned LLM to the question "What is the non-accessible surface area of ARAHIM02."
Supplementary Figure S14. ChatMOF's response with fine-tuned Language Model (LLM) to the question "Provide the number of atoms and cell length of XEGKUR," compared with GPT-4.
Supplementary Figure S15. ChatMOF's response with fine-tuned Language Model (LLM) to the question "Provide the CO2 henry coefficient of XEGKUR at 298 K in mol/cm3Pa" compared with GPT-4.
Supplementary Figure S16. Evaluation of ChatMOF at various GPT temperatures.
Supplementary Figure S17. Comparison of Question Diversity between GPT-4, GPT-3.5-turbo, and Template-based Method.
Supplementary Table S1. 100 questions in search task
Supplementary Table S2. 100 questions in prediction task
Supplementary Table S3. 10 questions in generation task
Supplementary Table S4. Accuracy for search and prediction task using Llama-2-chat-7B-hf and Llama-2-chat-13B-hf model

Supplementary Note S1. Full description for example of generator for question "Can you generate the structures with the largest surface area".

>>> Console logs (under WARNING level) are disabled.

WARNING:root:File logs (under WARNING level) are disabled.

> Entering new ChatMOF chain...

########## Question ###############################

Can you generate the structures with the largest surface area?

> Entering new AgentExecutor chain...

The question is asking to generate a structure with the largest surface area. I can use the generator tool for this.

Action: generator

Action Input: "Generate the structure with the largest surface area"

> Entering new Generator chain...

[Generator] Thought: I need to generate a material with the largest surface area.

[Generator] Objective: max

[Generator] Predict Properties: accessible_surface_area

load model : /home/dudgns1675/ChatMOF/chatmof/database/load_model/accessible_surface_area/best.ckpt

Predicting DataLoader 0: 100%|██████████| 178/178 [00:11<00:00, 15.56it/s]

load model : /home/dudgns1675/ChatMOF/chatmof/database/load_model/accessible_surface_area/best.ckpt

Predicting DataLoader 0: 100%|██████████| 160/160 [00:12<00:00, 13.25it/s]

load model : /home/dudgns1675/ChatMOF/chatmof/database/load_model/accessible_surface_area/best.ckpt

Predicting DataLoader 0: 100%|██████████| 157/157 [00:10<00:00, 15.13it/s]

load model : /home/dudgns1675/ChatMOF/chatmof/database/load_model/accessible_surface_area/best.ckpt

Predicting DataLoader 0: 100%|██████████| 149/149 [00:09<00:00, 15.23it/s]

load model : /home/dudgns1675/ChatMOF/chatmof/database/load_model/accessible_surface_area/best.ckpt

Predicting DataLoader 0: 100%|██████████| 169/169 [00:10<00:00, 15.83it/s]

load model : /home/dudgns1675/ChatMOF/chatmof/database/load_model/accessible_surface_area/best.ckpt

Predicting DataLoader 0: 100%|██████████| 150/150 [00:11<00:00, 13.62it/s]

load model : /home/dudgns1675/ChatMOF/chatmof/database/load_model/accessible_surface_area/best.ckpt

Predicting DataLoader 0: 100%|██████████| 81/81 [00:05<00:00, 14.39it/s]

load model : /home/dudgns1675/ChatMOF/chatmof/database/load_model/accessible_surface_area/best.ckpt

Predicting DataLoader 0: 100%|██████████| 173/173 [00:11<00:00, 14.86it/s]

load model : /home/dudgns1675/ChatMOF/chatmof/database/load_model/accessible_surface_area/best.ckpt

Predicting DataLoader 0: 100%|██████████| 108/108 [00:06<00:00, 15.49it/s]

[Generator] Run genetic algorithm : Cycle 1.

[Generator] Find Parents: extract cif_id and accessible_surface_area of 100 materials with the largest surface area from look-up tables.

> Entering new TableSearcher chain...

[Table Searcher] Thought: To extract the top 100 materials with the largest surface area, we can sort the DataFrame by the 'accessible_surface_area' column in descending order and then use the head() function to get the first 100 rows.

[Table Searcher] Input: print(df.sort_values('accessible_surface_area', ascending=False).head(100).to_markdown())

[Table Searcher] Observation:

	cif_id	accessible_surface_area
150	pcu+N47+E33	5808.59
2051	pcu+N274+E32	5723.77
2284	pcu+N234+E229	5665.73
2826	pcu+N613+E106	5661.26
419	pcu+N85+E77	5656.8
314	pcu+N694+E10	5647.87
861	pcu+N559+E61	5643.41
2525	pcu+N613+E90	5643.41
339	pcu+N613+E6	5638.94
1547	pcu+N47+E41	5638.94
1900	pcu+N574+E30	5616.62
1181	pcu+N613+E223	5612.16
...	.....	...

> Finished chain.

> Entering new TableSearcher chain...

[Table Searcher] Thought: To extract the top 100 materials with the largest surface area, we need to sort the DataFrame by the 'accessible_surface_area' column in descending order and then use the head() function to get the top 100 rows.

```
[Table Searcher] Input: import pandas as pd
import json
df_sorted = df.sort_values(by='accessible_surface_area', ascending=False)
print(df_sorted.head(100).to_markdown())
[Table Searcher] Observation:
|      | cif_id         |   accessible_surface_area |
|-----:|:---------------|--------------------------:|
|  430 | dia+N515+E23   |                   6375.57 |
| 1238 | dia+N515+E24   |                   6362.17 |
|  180 | dia+N310+E3    |                   6295.21 |
| 2062 | dia+N649+E47   |                   6290.74 |
| 1807 | dia+N267+E23   |                   6250.56 |
| 1265 | dia+N310+E47   |                   6250.56 |
| 2136 | dia+N238+E21   |                   6241.63 |
| 2151 | dia+N76+E163   |                   6237.17 |
|  637 | dia+N697+E34   |                   6232.71 |
| 2158 | dia+N515+E17   |                   6232.71 |
|  383 | dia+N492+E28   |                   6232.71 |
|  539 | dia+N697+E35   |                   6232.71 |
| 1828 | dia+N515+E112  |                   6232.71 |
| 1756 | dia+N515+E32   |                   6228.24 |
| 2087 | dia+N310+E35   |                   6223.78 |
| 2315 | dia+N238+E47   |                   6214.85 |
| 1632 | dia+N435+E8    |                   6214.85 |
| 1877 | dia+N161+E32   |                   6214.85 |
|  762 | dia+N515+E42   |                   6210.38 |
| 2275 | dia+N113+E36   |                   6205.92 |
| 2288 | dia+N93+E151   |                   6205.92 |
```

```
| 1574 | dia+N93+E149  |                 6205.92 |
|  866 | dia+N238+E12  |                 6196.99 |
|  472 | dia+N161+E107 |                 6196.99 |
|  215 | dia+N93+E72   |                 6192.53 |
| 1131 | dia+N307+E80  |                 6183.6  |
||   ...   |      .....             |                  ...          |
```

> Entering new TableSearcher chain...

[Table Searcher] Thought: To extract the top 100 materials with the largest surface area, we can use the sort_values() function in pandas to sort the DataFrame by the 'accessible_surface_area' column in descending order. Then, we can use the head() function to get the top 100 rows.

[Table Searcher] Input: import pandas as pd

```
import json
df_sorted = df.sort_values(by='accessible_surface_area', ascending=False)
print(df_sorted[['cif_id', 'accessible_surface_area']].head(100).to_markdown())
```

[Table Searcher] Observation:

```
|      | cif_id        |   accessible_surface_area |
|-----:|:--------------|--------------------------:|
| 1247 | acs+N489+E10  |                   6000.56 |
| 1747 | acs+N553+E10  |                   5978.24 |
|  469 | acs+N481+E10  |                   5960.38 |
| 1781 | acs+N628+E194 |                   5871.09 |
|  582 | acs+N536+E39  |                   5835.38 |
| 2035 | acs+N343+E39  |                   5830.91 |
|  143 | acs+N481+E39  |                   5821.98 |
|  614 | acs+N125+E39  |                   5772.87 |
```

| 766 | acs+N287+E10 | 5759.48 |

| 2296 | acs+N589+E39 | 5755.02 |

| 1229 | acs+N481+E194 | 5750.55 |

| 2156 | acs+N628+E9 | 5746.09 |

| 1641 | acs+N553+E39 | 5732.69 |

| 1068 | acs+N489+E39 | 5723.77 |

| ... | ..... | ... |

> Finished chain.

> Entering new TableSearcher chain...

[Table Searcher] Thought: To extract the top 100 materials with the largest surface area, we can use the sort_values() function in pandas to sort the DataFrame by the 'accessible_surface_area' column in descending order. Then, we can use the head() function to get the top 100 rows.

[Table Searcher] Input: import pandas as pd

import json

print(df.sort_values(by='accessible_surface_area', ascending=False).head(100).to_markdown())

[Table Searcher] Observation:

| | cif_id | accessible_surface_area |

|-----:|:--------------|--------------------------:|

| 991 | rtl+N535+N222 | 6313.06 |

| 1503 | rtl+N535+N680 | 6259.49 |

| 1399 | rtl+N535+N162 | 6259.49 |

| 497 | rtl+N535+N613 | 6255.03 |

| 321 | rtl+N535+N668 | 6250.56 |

| 2361 | rtl+N535+N213 | 6241.63 |

| 1288 | rtl+N535+N483 | 6232.71 |

```
| 2360 | rtl+N535+N465 |                 6223.78 |
|  405 | rtl+N535+N106 |                 6183.6  |
|  701 | rtl+N535+N598 |                 6170.2  |
|   ...   |      .....             |                  ...        |
> Finished chain.



> Entering new TableSearcher chain...


[Table Searcher] Thought: To extract the top 100 materials with the largest surface area, we can sort the DataFrame by the 'accessible_surface_area' column in descending order and then use the head() function to get the first 100 rows.
[Table Searcher] Input: print(df.sort_values(by='accessible_surface_area', ascending=False).head(100).to_markdown())
[Table Searcher] Observation:
|      | cif_id        |   accessible_surface_area |
|-----:|:--------------|--------------------------:|
| 2396 | cds+N112+E32  |                 5933.59 |
| 2417 | cds+N654+E32  |                 5929.13 |
|  508 | cds+N561+E32  |                 5915.73 |
| 1395 | cds+N464+E224 |                 5862.16 |
|  959 | cds+N464+E234 |                 5853.23 |
| 1369 | cds+N292+E176 |                 5839.84 |
| 1037 | cds+N470+E7   |                 5839.84 |
| 1063 | cds+N193+E41  |                 5826.45 |
|  515 | cds+N551+E33  |                 5808.59 |
| 1669 | cds+N259+E98  |                 5804.12 |
|  334 | cds+N512+E33  |                 5795.2  |
|  930 | cds+N503+E224 |                 5790.73 |
```

```
| 1666 | cds+N193+E22  |                  5786.27 |
| 1674 | cds+N194+E152 |                  5781.8  |
| 2577 | cds+N193+E33  |                  5781.8  |
|  ...  |      .....              |                   ...         |
> Finished chain.



> Entering new TableSearcher chain...

[Table Searcher] Thought: To extract the top 100 materials with the largest surface area, we can sort the DataFrame by the 'accessible_surface_area' column in descending order and then use the head() function to get the top 100 rows.
[Table Searcher] Input: import pandas as pd
import json
df_sorted = df.sort_values(by='accessible_surface_area', ascending=False)
print(df_sorted.head(100).to_markdown())
[Table Searcher] Observation:
|      | cif_id        |   accessible_surface_area |
|-----:|:--------------|--------------------------:|
|  235 | srs+N285+E129 |                  6063.06 |
| 2112 | srs+N606+E45  |                  6058.6  |
|  723 | srs+N298+E63  |                  6058.6  |
|  278 | srs+N298+E128 |                  6054.13 |
|  319 | srs+N285+E172 |                  6049.67 |
| 1110 | srs+N298+E168 |                  6045.2  |
| 1655 | srs+N298+E212 |                  6040.74 |
| 2290 | srs+N606+E14  |                  6036.27 |
| 2152 | srs+N611+E58  |                  6031.81 |
```

```
| 1126 | srs+N606+E180 |                 6031.81 |
| 1560 | srs+N298+E151 |                 6018.42 |
|  ...  |     .....           |                ...        |
> Finished chain.



> Entering new TableSearcher chain...


[Table Searcher] Thought: To extract the top 100 materials with the largest surface area, we can sort the DataFrame by the 'accessible_surface_area' column in descending order and then use the head() function to get the first 100 rows.
[Table Searcher] Input: import pandas as pd
import json
df_sorted = df.sort_values(by='accessible_surface_area', ascending=False)
print(df_sorted.head(100).to_markdown())
[Table Searcher] Observation:
|      | cif_id        |   accessible_surface_area |
|-----:|:--------------|--------------------------:|
| 1274 | ths+N606+E8   |                   5853.23 |
|  193 | ths+N298+E81  |                   5835.38 |
|  605 | ths+N96+E149  |                   5835.38 |
|  597 | ths+N606+E23  |                   5830.91 |
| 1151 | ths+N606+E11  |                   5830.91 |
|  147 | ths+N411+E27  |                   5821.98 |
|  293 | ths+N411+E44  |                   5808.59 |
|  506 | ths+N298+E168 |                   5804.12 |
|  632 | ths+N606+E156 |                   5804.12 |
| 1185 | ths+N606+E26  |                   5795.2  |
```

```
|  499 | ths+N606+E24  |                5795.2  |
|  494 | ths+N298+E167 |                5795.2  |
|  861 | ths+N606+E195 |                5786.27 |
| 1251 | ths+N96+E19   |                5772.87 |
|  403 | ths+N606+E219 |                5772.87 |
|  508 | ths+N606+E112 |                5750.55 |
|  ...  |     .....          |                ...        |
> Finished chain.



> Entering new TableSearcher chain...
```

[Table Searcher] Thought: To extract the top 100 materials with the largest surface area, we can sort the DataFrame by the 'accessible_surface_area' column in descending order and then use the head() function to get the top 100 rows.

[Table Searcher] Input: import pandas as pd

```
import json
df_sorted = df.sort_values(by='accessible_surface_area', ascending=False)
print(df_sorted.head(100).to_markdown())
```

[Table Searcher] Observation:

```
|      | cif_id        |   accessible_surface_area |
|-----:|:--------------|--------------------------:|
|    3 | bcu+N672+E229 |                   6152.35 |
| 1191 | bcu+N293+E39  |                   6040.74 |
| 1097 | bcu+N204+E10  |                   5951.45 |
|  438 | bcu+N293+E1   |                   5911.27 |
| 1740 | bcu+N293+E121 |                   5902.34 |
|  403 | bcu+N372+E10  |                   5902.34 |
```

```
| 1652 | bcu+N204+E39  |                  5897.88 |
|   21 | bcu+N293+E36  |                  5884.48 |
| 1389 | bcu+N656+E10  |                  5746.09 |
| 1720 | bcu+N642+E229 |                  5732.69 |
|  492 | bcu+N64+E225  |                  5728.23 |
| 2139 | bcu+N293+E21  |                  5701.44 |
| 1115 | bcu+N228+E39  |                  5661.26 |
| 1252 | bcu+N672+E59  |                  5614.39 |
| 1071 | bcu+N293+E40  |                  5601    |
| 2181 | bcu+N293+E126 |                  5576.44 |
| 1032 | bcu+N281+E10  |                  5547.42 |
|  856 | bcu+N228+E28  |                  5525.1  |
| 2106 | bcu+N281+E194 |                  5502.78 |
| 1053 | bcu+N212+E39  |                  5482.69 |
|  985 | bcu+N64+E223  |                  5431.35 |
| 2672 | bcu+N362+E1   |                  5417.96 |
|  730 | bcu+N637+E10  |                  5413.49 |
|  565 | bcu+N293+E176 |                  5404.56 |
|  304 | bcu+N189+E10  |                  5404.56 |
|  430 | bcu+N189+E39  |                  5346.53 |
| 1104 | bcu+N64+E82   |                  5344.29 |
|  ...  |     .....          |                  ...       |
> Finished chain.


> Entering new TableSearcher chain...
```

[Table Searcher] Thought: To extract the top 100 materials with the largest surface area, we can sort the DataFrame by the 'accessible_surface_area' column in descending order and then use the head() function to get the first 100 rows.

[Table Searcher] Input: import pandas as pd

import json

df_sorted = df.sort_values(by='accessible_surface_area', ascending=False)

print(df_sorted.head(100).to_markdown())

[Table Searcher] Observation:

	cif_id	accessible_surface_area
35	fsc+N614+N483	4279.54
1511	fsc+N255+N214	4263.91
342	fsc+N614+N457	4241.59
621	fsc+N201+N457	4159
885	fsc+N614+N678	4154.54
1220	fsc+N201+N483	4049.62
1524	fsc+N201+N208	4042.93
420	fsc+N255+N392	4022.84
1677	fsc+N129+N208	3980.42
1609	fsc+N665+N457	3978.19
1411	fsc+N665+N208	3944.71
1676	fsc+N255+N521	3929.08
669	fsc+N614+N521	3913.46
1241	fsc+N665+N483	3902.3
220	fsc+N201+N392	3897.83
981	fsc+N614+N214	3891.14
1617	fsc+N255+N274	3882.21
1439	fsc+N255+N678	3877.74

```
| 1401 | fsc+N255+N457 |                3859.89 |
| 1057 | fsc+N12+N457  |                3855.42 |
| 1171 | fsc+N12+N208  |                3848.72 |
| 1386 | fsc+N12+N483  |                3826.4  |
| 1425 | fsc+N614+N680 |                3819.71 |
|  913 | fsc+N614+N570 |                3750.51 |
|  ...  |     .....          |                ...        |
```

> Finished chain.

[Generator] Get Children: generate 100 new materials with the largest surface area from the 100 materials.

pcu: ['N47+E32', 'N274+E33', 'N234+E47', 'N613+E274', 'N85+E613', 'N694+E85', 'N559+E694', 'N613+E559', 'N613+E613', 'N47+E613', 'N574+E47', 'N613+E574', 'N668+E613', 'N47+E668', 'N628+E47', 'N559+E628', 'N678+E559', 'N680+E678', 'N406+E680', 'N483+E406', 'N274+E483', 'N274+E274', 'N613+E274', 'N213+E613', 'N694+E213', 'N180+E694', 'N222+E180', 'N680+E222', 'N222+E680', 'N47+E222', 'N180+E47', 'N392+E180', 'N595+E392', 'N483+E595', 'N167+E483', 'N540+E167', 'N180+E540', 'N222+E180', 'N657+E222', 'N222+E657', 'N657+E657', 'N392+E657', 'N222+E392', 'N47+E222', 'N559+E47', 'N392+E559', 'N598+E392', 'N678+E598', 'N392+E678', 'N598+E392', 'N162+E598', 'N274+E162', 'N483+E274', 'N483+E483', 'N680+E483', 'N668+E680', 'N214+E668', 'N483+E214', 'N521+E483', 'N480+E521', 'N234+E480', 'N295+E234', 'N483+E295', 'N694+E483', 'N559+E694', 'N55+E559', 'N392+E55', 'N613+E392', 'N341+E613', 'N148+E341', 'N465+E148', 'N613+E465', 'N483+E613', 'N295+E483', 'N180+E295', 'N678+E180', 'N214+E678', 'N392+E214', 'N162+E392', 'N680+E162', 'N392+E680', 'N483+E392', 'N213+E483', 'N680+E213', 'N205+E680', 'N598+E205', 'N16+E598', 'N16+E16', 'N240+E16', 'N336+E240', 'N309+E336', 'N489+E309', 'N341+E489', 'N105+E341', 'N483+E105', 'N595+E483', 'N162+E595', 'N392+E162', 'N274+E392', 'N55+E274']

dia: ['N515+E3', 'N515+E47', 'N310+E23', 'N310+E24', 'N649+E23', 'N649+E24', 'N267+E24', 'N267+E32', 'N238+E23', 'N238+E32', 'N76+E23', 'N76+E32', 'N697+E23', 'N697+E24', 'N492+E23', 'N492+E24', 'N113+E23', 'N113+E24', 'N93+E23', 'N93+E24', 'N307+E23', 'N307+E24', 'N2+E23', 'N2+E24', 'N156+E23', 'N156+E24', 'N50+E23', 'N50+E24', 'N68+E23', 'N68+E24', 'N188+E23', 'N188+E24', 'N398+E23', 'N398+E24', 'N371+E23', 'N371+E24', 'N256+E23', 'N256+E24', 'N219+E23', 'N219+E24', 'N515+E310', 'N515+E649', 'N310+E267', 'N310+E238', 'N649+E76', 'N649+E697', 'N267+E492', 'N267+E113', 'N238+E93', 'N238+E307', 'N76+E2', 'N76+E156', 'N697+E50', 'N697+E68', 'N492+E188', 'N492+E398', 'N113+E371', 'N113+E256', 'N93+E219', 'N93+E515', 'N307+E310', 'N307+E649', 'N2+E267',

'N2+E238', 'N156+E76', 'N156+E697', 'N50+E492', 'N50+E113', 'N68+E93', 'N68+E307', 'N188+E2', 'N188+E156', 'N398+E50', 'N398+E68', 'N371+E188', 'N371+E398', 'N256+E371', 'N256+E219', 'N219+E515', 'N219+E310', 'N515+E267', 'N515+E238', 'N310+E76', 'N310+E697', 'N649+E492', 'N649+E113', 'N267+E93', 'N267+E307', 'N238+E2', 'N238+E156', 'N76+E50', 'N76+E68', 'N697+E188', 'N697+E398', 'N492+E371', 'N492+E256', 'N113+E219', 'N113+E515', 'N93+E310', 'N93+E649', 'N307+E267', 'N307+E238', 'N2+E76', 'N2+E697', 'N156+E492', 'N156+E113', 'N50+E93', 'N50+E307', 'N68+E2', 'N68+E156', 'N188+E50', 'N188+E68', 'N398+E188', 'N398+E398', 'N371+E371', 'N371+E256', 'N256+E219', 'N256+E515', 'N219+E310', 'N219+E649']

acs: ['N489+E39', 'N481+E10', 'N628+E10', 'N536+E10', 'N343+E10', 'N125+E10', 'N287+E194', 'N589+E194', 'N481+E9', 'N628+E39', 'N553+E10', 'N489+E10', 'N198+E10', 'N270+E10', 'N553+E39', 'N481+E4', 'N287+E4', 'N536+E194', 'N553+E194', 'N125+E194', 'N589+E194', 'N198+E194', 'N489+E194', 'N198+E39', 'N574+E39', 'N628+E39', 'N553+E9', 'N489+E9', 'N198+E9', 'N574+E9', 'N628+E9', 'N553+E4', 'N489+E4', 'N198+E4', 'N574+E4', 'N628+E4', 'N553+E7', 'N489+E7', 'N198+E7', 'N574+E7', 'N628+E7', 'N553+E33', 'N489+E33', 'N198+E33', 'N574+E33', 'N628+E33', 'N553+E22', 'N489+E22', 'N198+E22', 'N574+E22', 'N628+E22', 'N553+E59', 'N489+E59', 'N198+E59', 'N574+E59', 'N628+E59', 'N553+E1', 'N489+E1', 'N198+E1', 'N574+E1', 'N628+E1', 'N553+E28', 'N489+E28', 'N198+E28', 'N574+E28', 'N628+E28', 'N553+E36', 'N489+E36', 'N198+E36', 'N574+E36', 'N628+E36', 'N553+E121', 'N489+E121', 'N198+E121', 'N574+E121', 'N628+E121', 'N553+E2', 'N489+E2', 'N198+E2', 'N574+E2', 'N628+E2', 'N553+E33', 'N489+E33', 'N198+E33', 'N574+E33', 'N628+E33', 'N553+E9', 'N489+E9', 'N198+E9', 'N574+E9', 'N628+E9', 'N553+E229', 'N489+E229', 'N198+E229', 'N574+E229', 'N628+E229', 'N553+E10', 'N489+E10', 'N198+E10', 'N574+E10', 'N628+E10', 'N553+E39', 'N489+E39', 'N198+E39', 'N574+E39', 'N628+E39']

rtl: ['N234+N222', 'N535+N392', 'N535+N678', 'N535+N222', 'N535+N214', ‘N535+N234’, 'N535+N392', 'N535+N678', 'N386+N234', 'N386+N222', 'N386+N214', 'N386+N392', 'N386+N678', 'N48+N535', 'N48+N386', 'N48+N234', 'N48+N222', 'N48+N214', 'N48+N392', 'N48+N678', 'N126+N535', 'N126+N386', 'N126+N234', 'N126+N222', 'N126+N214', 'N126+N392', 'N126+N678', 'N535+N33', 'N535+N613', 'N535+N151', 'N535+N559', 'N535+N222', 'N535+N214', 'N535+N392', 'N535+N678', 'N386+N33', 'N386+N613', 'N386+N151', 'N386+N559', 'N386+N222', 'N386+N214', 'N386+N392', 'N386+N678', 'N48+N33', 'N48+N613', 'N48+N151', 'N48+N559', 'N48+N222', 'N48+N214', 'N48+N392', 'N48+N678', 'N126+N33', 'N126+N613', 'N126+N151', 'N126+N559', 'N126+N222', 'N126+N214', 'N126+N392', 'N126+N678', 'N535+N205', 'N535+N593', 'N535+N324', 'N535+N486', 'N535+N518', 'N535+N16', 'N535+N447', 'N535+N234', 'N535+N552', 'N535+N442', 'N535+N559', 'N535+N555', 'N535+N489', 'N535+N33', 'N535+N336', 'N535+N678', 'N386+N205', 'N386+N593', 'N386+N324', 'N386+N486', 'N386+N518', 'N386+N16', 'N386+N447', 'N386+N234', 'N386+N552', 'N386+N442', 'N386+N559', 'N386+N555', 'N386+N489', 'N386+N33', 'N386+N336', 'N386+N678', 'N48+N205', 'N48+N593', 'N48+N324', 'N48+N486', 'N48+N518', 'N48+N16', 'N48+N447', 'N48+N234', 'N48+N552', 'N48+N442', 'N48+N559', 'N48+N555', 'N48+N489', 'N48+N33', 'N48+N336',

'N48+N678', 'N126+N205', 'N126+N593', 'N126+N324', 'N126+N486', 'N126+N518', 'N126+N16', 'N126+N447', 'N126+N234', 'N126+N552', 'N126+N442', 'N126+N559', 'N126+N555', 'N126+N489', 'N126+N33', 'N126+N336', 'N126+N678']

cds: ['N112+E224', 'N654+E110', 'N561+E7', 'N464+E33', 'N292+E41', 'N470+E32', 'N193+E32', 'N551+E32', 'N259+E32', 'N512+E32', 'N503+E7', 'N193+E110', 'N194+E32', 'N193+E224', 'N259+E33', 'N194+E33', 'N409+E32', 'N551+E7', 'N500+E32', 'N194+E7', 'N211+E32', 'N557+E32', 'N500+E7', 'N365+E32', 'N464+E32', 'N365+E110', 'N557+E110', 'N259+E7', 'N445+E32', 'N689+E32', 'N464+E224', 'N618+E7', 'N259+E110', 'N194+E32', 'N500+E33', 'N654+E33', 'N342+E32', 'N500+E110', 'N335+E32', 'N355+E32', 'N464+E33', 'N211+E110', 'N174+E32', 'N618+E33', 'N365+E110', 'N512+E110', 'N200+E32', 'N259+E32', 'N654+E33', 'N302+E32', 'N464+E110', 'N194+E224', 'N500+E7', 'N549+E32', 'N292+E110', 'N654+E7', 'N259+E33', 'N112+E32', 'N194+E110', 'N561+E110', 'N153+E32', 'N557+E224', 'N335+E7', 'N27+E32', 'N112+E110', 'N259+E7', 'N503+E32', 'N259+E224', 'N112+E7', 'N424+E32', 'N317+E32', 'N335+E110', 'N292+E33', 'N618+E7', 'N654+E110', 'N409+E110', 'N561+E33', 'N618+E110', 'N551+E32', 'N500+E110', 'N193+E110', 'N193+E33', 'N718+E110', 'N551+E7', 'N503+E7', 'N335+E224', 'N464+E7', 'N561+E7', 'N317+E110', 'N464+E32', 'N500+E33', 'N128+E32', 'N112+E224', 'N464+E7', 'N259+E110', 'N551+E33', 'N112+E32', 'N614+E32', 'N292+E33']

srs: ['N285+E45', 'N285+E63', 'N285+E128', 'N285+E168', 'N285+E212', 'N285+E14', 'N285+E58', 'N285+E180', 'N285+E151', 'N285+E8', 'N285+E71', 'N285+E37', 'N285+E16', 'N285+E132', 'N285+E29', 'N285+E216', 'N285+E18', 'N285+E205', 'N285+E16', 'N285+E188', 'N285+E166', 'N285+E29', 'N285+E146', 'N285+E130', 'N285+E221', 'N285+E67', 'N285+E25', 'N285+E220', 'N285+E66', 'N285+E201', 'N285+E186', 'N285+E81', 'N285+E139', 'N285+E119', 'N285+E206', 'N285+E81', 'N285+E108', 'N285+E226', 'N285+E182', 'N285+E27', 'N285+E52', 'N285+E159', 'N285+E24', 'N285+E58', 'N285+E204', 'N285+E6', 'N285+E23', 'N285+E221', 'N285+E26', 'N285+E181', 'N285+E108', 'N285+E190', 'N285+E98', 'N285+E134', 'N285+E37', 'N285+E173', 'N285+E187', 'N285+E50', 'N285+E190', 'N285+E204', 'N285+E48', 'N285+E151', 'N285+E220', 'N285+E167', 'N285+E173', 'N285+E87', 'N285+E224', 'N285+E171', 'N285+E160', 'N285+E167', 'N285+E5', 'N285+E19', 'N285+E129', 'N285+E49', 'N285+E113', 'N285+E171', 'N285+E85', 'N285+E73', 'N285+E180', 'N285+E11', 'N285+E129', 'N285+E79', 'N285+E175', 'N285+E11', 'N285+E149', 'N285+E70', 'N285+E61', 'N285+E16', 'N285+E168', 'N285+E47', 'N285+E52', 'N285+E175', 'N285+E138', 'N285+E72', 'N285+E174', 'N285+E129', 'N285+E70']

ths: ['N606+E81', 'N298+E149', 'N96+E8', 'N411+E23', 'N606+E12', 'N298+E11', 'N96+E27', 'N411+E44', 'N606+E168', 'N298+E156', 'N96+E26', 'N411+E24', 'N606+E167', 'N298+E195', 'N96+E219', 'N411+E112', 'N606+E8', 'N298+E13', 'N96+E128', 'N411+E72', 'N606+E76', 'N298+E136', 'N96+E16', 'N411+E57', 'N606+E27', 'N298+E127', 'N96+E216', 'N411+E220', 'N606+E182', 'N298+E216', 'N96+E26', 'N411+E187', 'N606+E18', 'N298+E15', 'N96+E81', 'N411+E182', 'N606+E158', 'N298+E128', 'N96+E207', 'N411+E108', 'N606+E13', 'N298+E95', 'N96+E85', 'N411+E181', 'N606+E187', 'N298+E130', 'N96+E148', 'N411+E45', 'N606+E180', 'N298+E234', 'N96+E201', 'N411+E72', 'N606+E180', 'N298+E159',

'N96+E234', 'N411+E146', 'N606+E43', 'N298+E216', 'N96+E86', 'N411+E76', 'N606+E138', 'N298+E71', 'N96+E132', 'N411+E44', 'N606+E130', 'N298+E120', 'N96+E190', 'N411+E34', 'N606+E151', 'N298+E178', 'N96+E48', 'N411+E16', 'N606+E128', 'N298+E186', 'N96+E150', 'N411+E175', 'N606+E113', 'N298+E131', 'N96+E86', 'N411+E195', 'N606+E81', 'N298+E112', 'N96+E162', 'N411+E13', 'N606+E52', 'N298+E171', 'N96+E187', 'N411+E137', 'N606+E189', 'N298+E191', 'N96+E92', 'N411+E204', 'N606+E160', 'N298+E69', 'N96+E175', 'N411+E138', 'N606+E127']

bcu: ['N672+E39', 'N293+E229', 'N204+E229', 'N293+E10', 'N372+E229', 'N204+E1', 'N293+E10', 'N656+E229', 'N642+E10', 'N64+E10', 'N293+E229', 'N228+E1', 'N672+E1', 'N293+E1', 'N293+E10', 'N281+E229', 'N228+E229', 'N281+E1', 'N212+E229', 'N64+E1', 'N362+E229', 'N637+E229', 'N293+E1', 'N189+E229', 'N189+E1', 'N64+E229', 'N629+E229', 'N664+E229', 'N84+E229', 'N281+E1', 'N204+E229', 'N204+E1', 'N281+E229', 'N26+E1', 'N281+E1', 'N587+E10', 'N64+E10', 'N141+E1', 'N204+E10', 'N212+E1', 'N204+E229', 'N642+E229', 'N281+E1', 'N293+E229', 'N637+E1', 'N293+E229', 'N637+E229', 'N587+E1', 'N672+E229', 'N204+E10', 'N587+E1', 'N149+E1', 'N281+E10', 'N262+E229', 'N281+E10', 'N293+E229', 'N431+E1', 'N204+E10', 'N212+E229', 'N362+E10', 'N141+E10', 'N281+E10', 'N626+E1', 'N84+E10', 'N281+E229', 'N262+E229', 'N26+E10', 'N204+E10', 'N642+E10', 'N626+E229', 'N293+E10', 'N293+E229', 'N626+E10', 'N228+E10', 'N228+E229', 'N637+E10', 'N228+E10', 'N64+E10', 'N281+E229', 'N189+E10', 'N120+E1', 'N45+E229', 'N672+E229', 'N35+E10', 'N204+E10', 'N226+E10', 'N45+E10', 'N35+E10', 'N228+E10', 'N370+E229', 'N350+E229', 'N642+E229', 'N637+E10', 'N69+E10', 'N204+E10', 'N587+E229', 'N362+E229', 'N199+E10', 'N204+E10']

fsc: ['N614+N392', 'N255+N457', 'N201+N214', 'N665+N483', 'N12+N208', 'N614+N274', 'N255+N208', 'N201+N678', 'N665+N392', 'N12+N214', 'N614+N457', 'N255+N678', 'N201+N274', 'N665+N208', 'N12+N678', 'N614+N392', 'N255+N214', 'N201+N457', 'N665+N678', 'N12+N274', 'N614+N208', 'N255+N392', 'N201+N214', 'N665+N457', 'N12+N392', 'N614+N678', 'N255+N457', 'N201+N392', 'N665+N214', 'N12+N457', 'N614+N274', 'N255+N208', 'N201+N678', 'N665+N392', 'N12+N208', 'N614+N457', 'N255+N678', 'N201+N274', 'N665+N208', 'N12+N678', 'N614+N392', 'N255+N214', 'N201+N457', 'N665+N678', 'N12+N274', 'N614+N208', 'N255+N392', 'N201+N214', 'N665+N457', 'N12+N392', 'N614+N678', 'N255+N457', 'N201+N392', 'N665+N214', 'N12+N457', 'N614+N274', 'N255+N208', 'N201+N678', 'N665+N392', 'N12+N208', 'N614+N457', 'N255+N678', 'N201+N274', 'N665+N208', 'N12+N678', 'N614+N392', 'N255+N214', 'N201+N457', 'N665+N678', 'N12+N274', 'N614+N208', 'N255+N392', 'N201+N214', 'N665+N457', 'N12+N392', 'N614+N678', 'N255+N457', 'N201+N392', 'N665+N214', 'N12+N457', 'N614+N274', 'N255+N208', 'N201+N678', 'N665+N392', 'N12+N208', 'N614+N457', 'N255+N678', 'N201+N274', 'N665+N208', 'N12+N678', 'N614+N392', 'N255+N214', 'N201+N457', 'N665+N678', 'N12+N274', 'N614+N208', 'N255+N392', 'N201+N214', 'N665+N457', 'N12+N392', 'N614+N678', 'N255+N457', 'N201+N392', 'N665+N214', 'N12+N457']

[Generator] Generate Structures:

pcu: 100%|██████████| 100/100 [00:11<00:00, 8.46it/s]

dia: 100%|██████████| 120/120 [00:50<00:00, 2.39it/s]

acs: 100%|██████████| 106/106 [00:43<00:00, 2.46it/s]

rtl: 100%|██████████| 123/123 [00:58<00:00, 2.10it/s]

cds: 100%|██████████| 99/99 [00:32<00:00, 3.08it/s]

srs: 100%|██████████| 97/97 [00:00<00:00, 159.10it/s]

ths: 100%|██████████| 97/97 [00:46<00:00, 2.10it/s]

bcu: 100%|██████████| 99/99 [00:38<00:00, 2.55it/s]

fsc: 100%|██████████| 105/105 [00:15<00:00, 6.67it/s]

[Generator] Predict Properties: accessible_surface_area

load model : /home/dudgns1675/ChatMOF/chatmof/database/load_model/accessible_surface_area/best.ckpt

Predicting DataLoader 0: 100%|██████████| 2/2 [00:00<00:00, 9.30it/s]

load model : /home/dudgns1675/ChatMOF/chatmof/database/load_model/accessible_surface_area/best.ckpt

Predicting DataLoader 0: 100%|██████████| 5/5 [00:00<00:00, 11.89it/s]

load model : /home/dudgns1675/ChatMOF/chatmof/database/load_model/accessible_surface_area/best.ckpt

Predicting DataLoader 0: 100%|██████████| 6/6 [00:00<00:00, 13.32it/s]

load model : /home/dudgns1675/ChatMOF/chatmof/database/load_model/accessible_surface_area/best.ckpt

Predicting DataLoader 0: 100%|██████████| 6/6 [00:00<00:00, 12.90it/s]

load model : /home/dudgns1675/ChatMOF/chatmof/database/load_model/accessible_surface_area/best.ckpt

Predicting DataLoader 0: 100%|██████████| 5/5 [00:00<00:00, 11.66it/s]

load model : /home/dudgns1675/ChatMOF/chatmof/database/load_model/accessible_surface_area/best.ckpt

Predicting DataLoader 0: 100%|██████████| 1/1 [00:00<00:00, 7.45it/s]

load model : /home/dudgns1675/ChatMOF/chatmof/database/load_model/accessible_surface_area/best.ckpt

Predicting DataLoader 0: 100%|██████████| 1/1 [00:00<00:00, 5.22it/s]

load model : /home/dudgns1675/ChatMOF/chatmof/database/load_model/accessible_surface_area/best.ckpt

Predicting DataLoader 0: 100%|██████████| 4/4 [00:00<00:00, 11.06it/s]

load model : /home/dudgns1675/ChatMOF/chatmof/database/load_model/accessible_surface_area/best.ckpt

Predicting DataLoader 0: 100%|██████████| 2/2 [00:00<00:00, 8.60it/s]

[Generator] Run genetic algorithm : Cycle 2.

[Generator] Find Parents: extract cif_id and accessible_surface_area of 100 materials with the largest surface area from look-up tables.

> Entering new TableSearcher chain...

[Table Searcher] Thought: To extract the top 100 materials with the largest surface area, we can sort the DataFrame by the 'accessible_surface_area' column in descending order and then use the head() function to get the top 100 rows.

[Table Searcher] Input: import pandas as pd

import json

df_sorted = df.sort_values(by='accessible_surface_area', ascending=False)

print(df_sorted.head(100).to_markdown())

[Table Searcher] Observation:

	cif_id	accessible_surface_area
150	pcu+N47+E33	5808.59
2051	pcu+N274+E32	5723.77

| 2284 | pcu+N234+E229 | 5665.73 |

| 2826 | pcu+N613+E106 | 5661.26 |

| 419 | pcu+N85+E77 | 5656.8 |

| 2853 | pcu+N274+E33 | 5652.34 |

| 314 | pcu+N694+E10 | 5647.87 |

| 861 | pcu+N559+E61 | 5643.41 |

| 2525 | pcu+N613+E90 | 5643.41 |

| 339 | pcu+N613+E6 | 5638.94 |

| 1547 | pcu+N47+E41 | 5638.94 |

| 1900 | pcu+N574+E30 | 5616.62 |

| ... | ..... | ... |

> Finished chain.

> Entering new TableSearcher chain...

[Table Searcher] Thought: To extract the top 100 materials with the largest surface area, we need to sort the DataFrame by the 'accessible_surface_area' column in descending order and then use the head() function to get the first 100 rows.

[Table Searcher] Input: print(df.sort_values(by='accessible_surface_area', ascending=False).head(100).to_markdown())

[Table Searcher] Observation:

| | cif_id | accessible_surface_area |

|-----:|:--------------|-------------------------:|

| 430 | dia+N515+E23 | 6375.57 |

| 1238 | dia+N515+E24 | 6362.17 |

| 2556 | dia+N267+E24 | 6326.46 |

| 2613 | dia+N515+E3 | 6308.6 |

| 180 | dia+N310+E3 | 6295.21 |

```
| 2062 | dia+N649+E47  |                6290.74 |
| 2569 | dia+N697+E24  |                6286.28 |
| 2566 | dia+N310+E24  |                6250.56 |
| 1807 | dia+N267+E23  |                6250.56 |
| 1265 | dia+N310+E47  |                6250.56 |
| 2598 | dia+N76+E23   |                6246.1  |
| 2618 | dia+N238+E23  |                6246.1  |
| 2136 | dia+N238+E21  |                6241.63 |
|  ...  |      .....      |              ...        |
> Finished chain.



> Entering new TableSearcher chain...
```

[Table Searcher] Thought: To extract the top 100 materials with the largest surface area, we can sort the DataFrame by the 'accessible_surface_area' column in descending order and then use the head() function to get the first 100 rows.

[Table Searcher] Input: print(df.sort_values(by='accessible_surface_area', ascending=False).head(100).to_markdown())

[Table Searcher] Observation:

```
|      | cif_id        |   accessible_surface_area |
|-----:|:--------------|--------------------------:|
| 2555 | acs+N628+E39  |                6228.24 |
| 2538 | acs+N574+E10  |                6143.42 |
| 2559 | acs+N553+E10  |                6121.1  |
| 2568 | acs+N198+E10  |                6080.92 |
| 2569 | acs+N198+E39  |                6045.2  |
| 1247 | acs+N489+E10  |                6000.56 |
| 1747 | acs+N553+E10  |                5978.24 |
```

```
|  469 | acs+N481+E10  |                 5960.38 |
| 2552 | acs+N553+E39  |                 5880.02 |
| 2514 | acs+N628+E10  |                 5875.56 |
| 2536 | acs+N489+E10  |                 5875.56 |
| 1781 | acs+N628+E194 |                 5871.09 |
| 2586 | acs+N198+E9   |                 5857.7  |
| 2570 | acs+N553+E229 |                 5848.77 |
|  582 | acs+N536+E39  |                 5835.38 |
| 2035 | acs+N343+E39  |                 5830.91 |
|   ...   |      .....            |                   ...         |
> Finished chain.



> Entering new TableSearcher chain...


[Table Searcher] Thought: To extract the top 100 materials with the largest surface area, we can sort the DataFrame by the 'accessible_surface_area' column in descending order and then use the head() function to get the top 100 rows.
[Table Searcher] Input: import pandas as pd
import json
df_sorted = df.sort_values(by='accessible_surface_area', ascending=False)
print(df_sorted.head(100).to_markdown())
[Table Searcher] Observation:
|      | cif_id        |   accessible_surface_area |
|-----:|:--------------|--------------------------:|
| 2400 | rtl+N535+N234 |                 6411.28|
| 2400 | rtl+N535+N392 |                 6375.57 |
|  991 | rtl+N535+N222 |                 6313.06 |
```

```
| 2395 | rtl+N535+N234 |                 6313.06 |
| 1503 | rtl+N535+N680 |                 6259.49 |
| 1399 | rtl+N535+N162 |                 6259.49 |
|  497 | rtl+N535+N613 |                 6255.03 |
|  321 | rtl+N535+N668 |                 6250.56 |
| 2361 | rtl+N535+N213 |                 6241.63 |
| 1288 | rtl+N535+N483 |                 6232.71 |
| 2386 | rtl+N535+N613 |                 6228.24 |
| 2360 | rtl+N535+N465 |                 6223.78 |
| 2405 | rtl+N535+N222 |                 6188.06 |
|  405 | rtl+N535+N106 |                 6183.6  |
|  701 | rtl+N535+N598 |                 6170.2  |
|   28 | rtl+N535+N590 |                 6161.28 |
|  747 | rtl+N535+N289 |                 6161.28 |
|  205 | rtl+N535+N283 |                 6147.88 |
| 2445 | rtl+N535+N678 |                 6147.88 |
|  ...  |     .....         |                 ...        |
```

> Finished chain.

> Entering new TableSearcher chain...

[Table Searcher] Thought: To extract the top 100 materials with the largest surface area, we can sort the DataFrame by the 'accessible_surface_area' column in descending order and then use the head() function to get the top 100 rows.

[Table Searcher] Input: print(df.sort_values(by='accessible_surface_area', ascending=False).head(100).to_markdown())

[Table Searcher] Observation:

```
|      | cif_id        |   accessible_surface_area |
```

```
|-----:|:--------------|--------------------------:|
| 2720 | cds+N259+E224 |                  5964.84 |
| 2396 | cds+N112+E32  |                  5933.59 |
| 2417 | cds+N654+E32  |                  5929.13 |
| 2737 | cds+N464+E224 |                  5920.2  |
|  508 | cds+N561+E32  |                  5915.73 |
| 2765 | cds+N112+E32  |                  5906.81 |
| 2746 | cds+N194+E224 |                  5902.34 |
| 2740 | cds+N500+E32  |                  5897.88 |
| 2768 | cds+N500+E33  |                  5893.41 |
| 2725 | cds+N512+E32  |                  5862.16 |
| 1395 | cds+N464+E224 |                  5862.16 |
|  959 | cds+N464+E234 |                  5853.23 |
| 2742 | cds+N194+E32  |                  5853.23 |
| 2718 | cds+N654+E33  |                  5848.77 |
| 1369 | cds+N292+E176 |                  5839.84 |
| 1037 | cds+N470+E7   |                  5839.84 |
| 2749 | cds+N551+E32  |                  5830.91 |
|  ...  |     .....          |                  ...        |
> Finished chain.



> Entering new TableSearcher chain...



[Table Searcher] Thought: To extract the top 100 materials with the largest surface area, we can sort the DataFrame by the 'accessible_surface_area' column in descending order and then use the head() function to get the first 100 rows.

[Table Searcher] Input: import pandas as pd
```

```
import json
df_sorted = df.sort_values(by='accessible_surface_area', ascending=False)
print(df_sorted.head(100).to_markdown())
[Table Searcher] Observation:
|      | cif_id         |   accessible_surface_area |
|-----:|:---------------|--------------------------:|
|  235 | srs+N285+E129 |                   6063.06 |
|  723 | srs+N298+E63  |                   6058.6  |
| 2112 | srs+N606+E45  |                   6058.6  |
|  278 | srs+N298+E128 |                   6054.13 |
|  319 | srs+N285+E172 |                   6049.67 |
| 1110 | srs+N298+E168 |                   6045.2  |
| 1655 | srs+N298+E212 |                   6040.74 |
| 2290 | srs+N606+E14  |                   6036.27 |
| 2152 | srs+N611+E58  |                   6031.81 |
| 1126 | srs+N606+E180 |                   6031.81 |
| 1470 | srs+N606+E8   |                   6018.42 |
| 1560 | srs+N298+E151 |                   6018.42 |
|  910 | srs+N298+E71  |                   6018.42 |
| 1846 | srs+N298+E37  |                   6013.95 |
|   ...  |      .....             |                   ...         |
> Finished chain.



> Entering new TableSearcher chain...
```

[Table Searcher] Thought: To extract the top 100 materials with the largest surface area, we can sort the DataFrame by the 'accessible_surface_area' column in descending order and then use the head() function to get the first 100 rows.

[Table Searcher] Input: import pandas as pd

```
import json

df_sorted = df.sort_values(by='accessible_surface_area', ascending=False)

print(df_sorted.head(100).to_markdown())
```

[Table Searcher] Observation:

```
|      | cif_id        |   accessible_surface_area |
|-----:|:--------------|--------------------------:|
| 1274 | ths+N606+E8   |                   5853.23 |
|  605 | ths+N96+E149  |                   5835.38 |
|  193 | ths+N298+E81  |                   5835.38 |
|  597 | ths+N606+E23  |                   5830.91 |
| 1151 | ths+N606+E11  |                   5830.91 |
|  147 | ths+N411+E27  |                   5821.98 |
| 1284 | ths+N411+E44  |                   5821.98 |
|  293 | ths+N411+E44  |                   5808.59 |
|  632 | ths+N606+E156 |                   5804.12 |
|  ... |      .....    |                     ...   |
```

> Finished chain.

> Entering new TableSearcher chain...

[Table Searcher] Thought: To extract the top 100 materials with the largest surface area, we can sort the DataFrame by the 'accessible_surface_area' column in descending order and then use the head() function to get the top 100 rows.

[Table Searcher] Input: top_100 = df.sort_values(by='accessible_surface_area', ascending=False).head(100)

```
print(top_100[['cif_id', 'accessible_surface_area']].to_markdown())
```

[Table Searcher] Observation:

```
|      | cif_id         |   accessible_surface_area |
|-----:|:---------------|--------------------------:|
| 2794 | bcu+N642+E10   |                   6219.31 |
|    3 | bcu+N672+E229  |                   6152.35 |
| 2757 | bcu+N293+E10   |                   6112.17 |
| 2796 | bcu+N26+E10    |                   6080.92 |
| 2775 | bcu+N64+E10    |                   6067.52 |
| 1191 | bcu+N293+E39   |                   6040.74 |
| 2768 | bcu+N228+E229  |                   6027.34 |
| 2762 | bcu+N293+E1    |                   6009.49 |
| 2758 | bcu+N69+E10    |                   5955.91 |
| 1097 | bcu+N204+E10   |                   5951.45 |
||   ...   |      .....       |                 ...        |
```

> Finished chain.

> Entering new TableSearcher chain...

[Table Searcher] Thought: To extract the top 100 materials with the largest surface area, we can sort the DataFrame by the 'accessible_surface_area' column in descending order and then use the head() function to get the top 100 rows.

[Table Searcher] Input:
```
import pandas as pd
import json
df_sorted = df.sort_values(by='accessible_surface_area', ascending=False)
print(df_sorted.head(100).to_markdown())
```

[Table Searcher] Observation:

	cif_id	accessible_surface_area
35	fsc+N614+N483	4279.54
1751	fsc+N255+N214	4266.14
1511	fsc+N255+N214	4263.91
1731	fsc+N614+N457	4243.82
342	fsc+N614+N457	4241.59
1750	fsc+N614+N208	4176.86
1735	fsc+N665+N392	4174.62
621	fsc+N201+N457	4159
885	fsc+N614+N678	4154.54
1753	fsc+N665+N457	4083.1
1741	fsc+N614+N392	4049.62
1220	fsc+N201+N483	4049.62
1524	fsc+N201+N208	4042.93
...	.....	...

> Finished chain.

[Generator] Get Children: generate 100 new materials with the largest surface area from the 100 materials.

Pcu: ['N47+E32', 'N274+E33', 'N234+E10', 'N613+E77', 'N85+E33', 'N274+E10', 'N694+E61', 'N559+E90', 'N613+E6', 'N613+E41', 'N47+E30', 'N574+E229', 'N613+E33', 'N668+E229', 'N628+E28', 'N47+E82', 'N559+E33', 'N678+E229', 'N406+E229', 'N680+E1', 'N274+E77', 'N483+E28', 'N274+E10', 'N613+E1', 'N213+E9', 'N180+E121', 'N694+E106', 'N222+E78', 'N680+E106', 'N222+E10', 'N47+E10', 'N180+E176', 'N47+E121', 'N392+E9', 'N595+E24', 'N483+E33', 'N167+E1', 'N540+E111', 'N222+E77', 'N180+E90', 'N657+E6', 'N222+E83', 'N657+E59', 'N392+E6', 'N47+E59', 'N559+E6', 'N222+E78', 'N392+E4', 'N598+E203', 'N678+E96', 'N392+E91', 'N598+E59', 'N162+E203', 'N274+E96', 'N483+E91', 'N483+E10', 'N680+E59', 'N483+E97', 'N214+E106', 'N668+E82', 'N521+E32', 'N47+E9', 'N234+E33', 'N480+E3', 'N483+E229', 'N295+E36', 'N694+E203', 'N559+E1', 'N55+E141', 'N392+E10', 'N341+E107', 'N613+E22', 'N148+E1', 'N465+E179', 'N613+E68', 'N483+E126', 'N295+E12', 'N180+E87', 'N678+E222', 'N680+E39', 'N214+E77', 'N392+E83', 'N162+E225', 'N680+E229', 'N213+E6', 'N392+E98', 'N483+E141', 'N680+E90', 'N598+E12', 'N16+E59', 'N205+E28',

'N16+E33', 'N240+E22', 'N309+E30', 'N336+E223', 'N489+E101', 'N341+E39', 'N483+E106', 'N595+E106', 'N105+E33']

dia: ['N515+E47', 'N267+E3', 'N310+E24', 'N649+E23', 'N697+E3', 'N310+E32', 'N267+E32', 'N310+E12', 'N76+E24', 'N238+E24', 'N238+E3', 'N76+E32', 'N515+E34', 'N492+E3', 'N515+E35', 'N697+E32', 'N697+E12', 'N515+E31', 'N310+E47', 'N515+E24', 'N238+E32', 'N161+E3', 'N435+E23', 'N76+E3', 'N515+E21', 'N93+E24', 'N113+E24', 'N93+E3', 'N161+E24', 'N238+E31', 'N93+E32', 'N68+E3', 'N2+E24', 'N76+E31', 'N267+E31', 'N310+E31', 'N307+E24', 'N68+E32', 'N310+E34', 'N76+E34', 'N310+E35', 'N515+E36', 'N76+E35', 'N492+E31', 'N2+E32', 'N492+E24', 'N156+E3', 'N649+E32', 'N492+E32', 'N515+E33', 'N310+E33', 'N649+E31', 'N76+E33', 'N697+E33', 'N310+E36', 'N492+E36', 'N435+E24', 'N161+E32', 'N50+E3', 'N310+E12', 'N2+E31', 'N93+E31', 'N68+E31', 'N267+E33', 'N307+E31', 'N2+E33', 'N2+E34', 'N515+E37', 'N238+E33', 'N93+E33', 'N310+E37', 'N188+E3', 'N2+E35', 'N515+E38', 'N238+E34', 'N435+E31', 'N515+E39', 'N310+E39', 'N2+E36', 'N307+E32', 'N719+E3', 'N93+E34', 'N93+E35', 'N708+E24', 'N68+E33', 'N697+E31', 'N515+E40', 'N93+E36', 'N113+E31', 'N113+E32', 'N238+E35', 'N708+E31', 'N492+E31', 'N158+E24', 'N697+E34', 'N398+E3', 'N515+E41', 'N50+E31']

acs: ['N628+E10', 'N574+E39', 'N553+E32', 'N198+E31', 'N198+E32', 'N489+E31', 'N553+E31', 'N481+E32', 'N553+E10', 'N628+E32', 'N489+E32', 'N628+E194', 'N198+E39', 'N553+E229', 'N536+E32', 'N343+E32', 'N628+E229', 'N481+E10', 'N481+E31', 'N125+E32', 'N287+E31', 'N198+E39', 'N589+E32', 'N481+E194', 'N628+E39', 'N553+E22', 'N553+E10', 'N198+E28', 'N489+E10', 'N198+E39', 'N125+E31', 'N270+E31', 'N553+E33', 'N270+E32', 'N553+E194', 'N553+E39', 'N553+E28', 'N343+E31', 'N481+E229', 'N553+E59', 'N287+E32', 'N536+E31', 'N589+E194', 'N574+E59', 'N553+E39', 'N553+E7', 'N125+E33', 'N489+E59', 'N489+E10', 'N574+E7', 'N198+E194', 'N553+E39', 'N628+E39', 'N589+E31', 'N574+E121', 'N198+E22', 'N489+E39', 'N553+E7', 'N489+E229', 'N553+E39', 'N553+E121', 'N198+E59', 'N574+E7', 'N489+E39', 'N628+E28', 'N553+E36', 'N489+E121', 'N489+E39', 'N481+E39', 'N422+E28', 'N86+E121', 'N198+E59', 'N574+E22', 'N198+E33', 'N536+E31', 'N489+E7', 'N574+E39', 'N574+E229', 'N574+E33', 'N553+E36', 'N489+E33', 'N102+E121', 'N422+E31', 'N198+E36', 'N489+E7', 'N691+E31', 'N134+E32', 'N574+E39', 'N574+E28', 'N628+E2', 'N553+E121', 'N447+E121', 'N553+E28', 'N489+E2', 'N574+E32', 'N198+E39', 'N554+E32', 'N328+E33', 'N198+E36', 'N628+E225']

rtl: ['N234+N392', 'N234+N222', 'N234+N680', 'N234+N162', 'N234+N613', 'N234+N668', 'N234+N213', 'N234+N483', 'N234+N465', 'N234+N106', 'N234+N598', 'N234+N590', 'N234+N289', 'N234+N283', 'N234+N678', 'N234+N521', 'N234+N559', 'N234+N341', 'N234+N696', 'N234+N16', 'N234+N309', 'N234+N151', 'N234+N593', 'N234+N457', 'N234+N336', 'N234+N148', 'N234+N214', 'N234+N504', 'N234+N36', 'N234+N569', 'N234+N508', 'N234+N480', 'N234+N311', 'N234+N33', 'N234+N447', 'N234+N135', 'N234+N205', 'N234+N105', 'N234+N630', 'N234+N453', 'N234+N529', 'N234+N491', 'N234+N65', 'N234+N146', 'N234+N518', 'N234+N366', 'N234+N250', 'N234+N324', 'N234+N555', 'N234+N486', 'N234+N552', 'N234+N442', 'N535+N386', 'N535+N48', 'N535+N126', 'N392+N222', 'N392+N680', 'N392+N162', 'N392+N613', 'N392+N668',

'N392+N213', 'N392+N483', 'N392+N465', 'N392+N106', 'N392+N598', 'N392+N590', 'N392+N289', 'N392+N283', 'N392+N678', 'N392+N521', 'N392+N559', 'N392+N341', 'N392+N696', 'N392+N16', 'N392+N309', 'N392+N151', 'N392+N593', 'N392+N457', 'N392+N336', 'N392+N148', 'N392+N214', 'N392+N504', 'N392+N36', 'N392+N569', 'N392+N508', 'N392+N480', 'N392+N311', 'N392+N33', 'N392+N447', 'N392+N135', 'N392+N205', 'N392+N105', 'N392+N630', 'N392+N453', 'N392+N529', 'N392+N491', 'N392+N65', 'N392+N146', 'N392+N518', 'N392+N366', 'N392+N250', 'N392+N324', 'N392+N555', 'N392+N486', 'N392+N552', 'N392+N442']

cds: ['N259+E32', 'N112+E224', 'N654+E224', 'N464+E32', 'N561+E224', 'N112+E224', 'N194+E32', 'N500+E224', 'N500+E224', 'N512+E224', 'N464+E32', 'N464+E32', 'N194+E224', 'N654+E224', 'N292+E32', 'N470+E224', 'N551+E224', 'N561+E224', 'N211+E224', 'N193+E224', 'N549+E224', 'N193+E32', 'N112+E32', 'N551+E32', 'N259+E32', 'N512+E224', 'N500+E32', 'N503+E32', 'N193+E224', 'N335+E32', 'N194+E32', 'N193+E32', 'N259+E224', 'N464+E224', 'N259+E32', 'N689+E224', 'N194+E32', 'N500+E224', 'N551+E32', 'N551+E224', 'N409+E224', 'N557+E224', 'N500+E32', 'N211+E224', 'N365+E224', 'N464+E32', 'N27+E224', 'N194+E32', 'N365+E224', 'N689+E224', 'N259+E32', 'N365+E224', 'N557+E224', 'N464+E224', 'N445+E224', 'N618+E32', 'N561+E224', 'N259+E224', 'N194+E224', 'N500+E32', 'N654+E32', 'N342+E224', 'N464+E32', 'N335+E224', 'N500+E32', 'N355+E224', 'N335+E224', 'N464+E32', 'N174+E224', 'N618+E32', 'N654+E32', 'N211+E224', 'N194+E224', 'N512+E224', 'N365+E224', 'N200+E224', 'N302+E224', 'N654+E32', 'N259+E224', 'N112+E32', 'N193+E224', 'N500+E32', 'N292+E224', 'N549+E224', 'N464+E224', 'N654+E32', 'N194+E224', 'N512+E32', 'N259+E224', 'N112+E32', 'N200+E224', 'N153+E224', 'N194+E224', 'N561+E224', 'N112+E224', 'N503+E224', 'N335+E224', 'N259+E32', 'N464+E224', 'N27+E224']

srs: ['N285+E63', 'N298+E129', 'N606+E129', 'N298+E45', 'N285+E32', 'N298+E172', 'N606+E172', 'N611+E45', 'N606+E14', 'N298+E14', 'N285+E14', 'N611+E172', 'N606+E168', 'N298+E180', 'N285+E168', 'N611+E168', 'N298+E8', 'N606+E8', 'N285+E8', 'N611+E8', 'N298+E71', 'N606+E71', 'N285+E71', 'N611+E71', 'N298+E37', 'N606+E37', 'N285+E37', 'N611+E37', 'N298+E16', 'N606+E16', 'N285+E16', 'N611+E16', 'N298+E132', 'N606+E132', 'N285+E132', 'N611+E132', 'N298+E29', 'N606+E29', 'N285+E29', 'N611+E29', 'N298+E25', 'N606+E25', 'N285+E25', 'N611+E25', 'N298+E66', 'N606+E66', 'N285+E66', 'N611+E66', 'N298+E186', 'N606+E186', 'N285+E186', 'N611+E186', 'N298+E119', 'N606+E119', 'N285+E119', 'N611+E119', 'N298+E81', 'N606+E81', 'N285+E81', 'N611+E81', 'N298+E27', 'N606+E27', 'N285+E27', 'N611+E27', 'N298+E159', 'N606+E159', 'N285+E159', 'N611+E159', 'N298+E24', 'N606+E24', 'N285+E24', 'N611+E24', 'N298+E6', 'N606+E6', 'N285+E6', 'N611+E6', 'N298+E181', 'N606+E181', 'N285+E181', 'N611+E181', 'N298+E23', 'N606+E23', 'N285+E23', 'N611+E23', 'N298+E26', 'N606+E26', 'N285+E26', 'N611+E26', 'N298+E98', 'N606+E98', 'N285+E98', 'N611+E98', 'N298+E37', 'N606+E37', 'N285+E37', 'N611+E37', 'N298+E173', 'N606+E173', 'N285+E173', 'N611+E173', 'N298+E187', 'N606+E187', 'N285+E187', 'N611+E187', 'N298+E50', 'N606+E50', 'N285+E50', 'N611+E50', 'N298+E48', 'N606+E48', 'N285+E48', 'N611+E48', 'N298+E204', 'N606+E204', 'N285+E204',

'N611+E204', 'N298+E167', 'N606+E167', 'N285+E167', 'N611+E167', 'N298+E220', 'N606+E220', 'N285+E220', 'N611+E220', 'N298+E173', 'N606+E173', 'N285+E173', 'N611+E173', 'N298+E87', 'N606+E87', 'N285+E87', 'N611+E87', 'N298+E224', 'N606+E224', 'N285+E224', 'N611+E224', 'N298+E171', 'N606+E171', 'N285+E171', 'N611+E171', 'N298+E5', 'N606+E5', 'N285+E5', 'N611+E5', 'N298+E160', 'N606+E160', 'N285+E160', 'N611+E160', 'N298+E129', 'N606+E129', 'N285+E129', 'N611+E129', 'N298+E19', 'N606+E19', 'N285+E19', 'N611+E19', 'N298+E113', 'N606+E113', 'N285+E113', 'N611+E113', 'N298+E49', 'N606+E49', 'N285+E49', 'N611+E49', 'N298+E171', 'N606+E171', 'N285+E171', 'N611+E171', 'N298+E85', 'N606+E85', 'N285+E85', 'N611+E85', 'N298+E73', 'N606+E73', 'N285+E73', 'N611+E73', 'N298+E11', 'N606+E11', 'N285+E11', 'N611+E11', 'N298+E79', 'N606+E79', 'N285+E79', 'N611+E79', 'N298+E175', 'N606+E175', 'N285+E175', 'N611+E175', 'N298+E149', 'N606+E149', 'N285+E149', 'N611+E149', 'N298+E70', 'N606+E70', 'N285+E70', 'N611+E70', 'N298+E61', 'N606+E61', 'N285+E61', 'N611+E61', 'N298+E16', 'N606+E16', 'N285+E16', 'N611+E16', 'N298+E168', 'N606+E168', 'N285+E168', 'N611+E168', 'N298+E47', 'N606+E47', 'N285+E47', 'N611+E47', 'N298+E52', 'N606+E52', 'N285+E52', 'N611+E52', 'N298+E175', 'N606+E175', 'N285+E175', 'N611+E175', 'N298+E72', 'N606+E72', 'N285+E72', 'N611+E72', 'N298+E138', 'N606+E138', 'N285+E138', 'N611+E138', 'N298+E129', 'N606+E129', 'N285+E129', 'N611+E129']

ths: ['N606+E149', 'N96+E8', 'N298+E8', 'N606+E12', 'N411+E12', 'N411+E8', 'N606+E32', 'N298+E32', 'N606+E11', 'N411+E11', 'N411+E31', 'N606+E31', 'N298+E31', 'N606+E24', 'N96+E24', 'N298+E24', 'N606+E26', 'N96+E26', 'N298+E26', 'N606+E195', 'N96+E195', 'N298+E195', 'N606+E219', 'N96+E219', 'N298+E219', 'N411+E13', 'N606+E13', 'N96+E13', 'N411+E8', 'N606+E8', 'N96+E8', 'N96+E216', 'N411+E216', 'N298+E216', 'N606+E216', 'N411+E128', 'N606+E128', 'N96+E128', 'N298+E72', 'N606+E72', 'N96+E72', 'N298+E76', 'N606+E76', 'N96+E76', 'N606+E136', 'N96+E136', 'N298+E136', 'N96+E16', 'N411+E16', 'N298+E16', 'N606+E16', 'N96+E57', 'N411+E57', 'N298+E57', 'N606+E57', 'N96+E27', 'N411+E27', 'N298+E27', 'N606+E27', 'N315+E216', 'N606+E216', 'N96+E216', 'N411+E216', 'N96+E127', 'N411+E127', 'N298+E127', 'N606+E127', 'N96+E220', 'N411+E220', 'N298+E220', 'N606+E220', 'N298+E182', 'N606+E182', 'N96+E182', 'N411+E182', 'N411+E26', 'N606+E26', 'N96+E26', 'N298+E26', 'N137+E216', 'N606+E216', 'N96+E216', 'N411+E216', 'N411+E187', 'N606+E187', 'N96+E187', 'N298+E187', 'N96+E18', 'N411+E18', 'N298+E18', 'N606+E18', 'N298+E15', 'N606+E15', 'N96+E15', 'N411+E15', 'N96+E182', 'N411+E182', 'N298+E182', 'N606+E182', 'N611+E81', 'N606+E81', 'N96+E81', 'N411+E81', 'N611+E108', 'N606+E108', 'N96+E108', 'N411+E108', 'N315+E128', 'N606+E128', 'N96+E128', 'N411+E128', 'N96+E158', 'N411+E158', 'N298+E158', 'N606+E158', 'N277+E207', 'N606+E207', 'N96+E207', 'N411+E207', 'N411+E95', 'N606+E95', 'N96+E95', 'N298+E95', 'N298+E13', 'N606+E13', 'N96+E13', 'N411+E13', 'N315+E85', 'N606+E85', 'N96+E85', 'N411+E85', 'N606+E187', 'N96+E187', 'N298+E187', 'N411+E187', 'N606+E181', 'N96+E181', 'N298+E181', 'N411+E181', 'N96+E130', 'N411+E130', 'N298+E130', 'N606+E130', 'N606+E148', 'N96+E148', 'N298+E148', 'N411+E148', 'N96+E45', 'N411+E45', 'N298+E45', 'N606+E45', 'N298+E180', 'N606+E180', 'N96+E180', 'N411+E180', 'N411+E85', 'N606+E85', 'N96+E85', 'N298+E85', 'N298+E201', 'N606+E201', 'N96+E201', 'N411+E201',

'N606+E234', 'N96+E234', 'N298+E234', 'N411+E234', 'N96+E72', 'N411+E72', 'N298+E72', 'N606+E72', 'N415+E180', 'N606+E180', 'N96+E180', 'N411+E180', 'N411+E138', 'N606+E138', 'N96+E138', 'N298+E138', 'N298+E43', 'N606+E43', 'N96+E43', 'N411+E43', 'N611+E146', 'N606+E146', 'N96+E146', 'N411+E146', 'N411+E234', 'N606+E234', 'N96+E234', 'N298+E234', 'N298+E159', 'N606+E159', 'N96+E159', 'N411+E159', 'N606+E86', 'N96+E86', 'N298+E86', 'N411+E86', 'N298+E216', 'N606+E216', 'N96+E216', 'N411+E216', 'N411+E76', 'N606+E76', 'N96+E76', 'N298+E76', 'N315+E138', 'N606+E138', 'N96+E138', 'N411+E138', 'N611+E132', 'N606+E132', 'N96+E132', 'N411+E132', 'N298+E71', 'N606+E71', 'N96+E71', 'N411+E71', 'N298+E130', 'N606+E130', 'N96+E130', 'N411+E130', 'N611+E44', 'N606+E44', 'N96+E44', 'N411+E44', 'N606+E190', 'N96+E190', 'N298+E190', 'N411+E190', 'N298+E120', 'N606+E120', 'N96+E120', 'N411+E120', 'N606+E34', 'N96+E34', 'N298+E34', 'N411+E34', 'N606+E178', 'N96+E178', 'N298+E178', 'N411+E178', 'N611+E151', 'N606+E151', 'N96+E151', 'N411+E151', 'N186+E48', 'N606+E48', 'N96+E48', 'N411+E48', 'N415+E186', 'N606+E186', 'N96+E186', 'N411+E186', 'N298+E128', 'N606+E128', 'N96+E128', 'N411+E128', 'N415+E16', 'N606+E16', 'N96+E16', 'N411+E16', 'N411+E150', 'N606+E150', 'N96+E150', 'N298+E150', 'N298+E175', 'N606+E175', 'N96+E175', 'N411+E175', 'N606+E113', 'N96+E113', 'N298+E113', 'N411+E113', 'N315+E131', 'N606+E131', 'N96+E131', 'N411+E131', 'N298+E86', 'N606+E86', 'N96+E86', 'N411+E86', 'N96+E195', 'N411+E195', 'N298+E195', 'N606+E195', 'N411+E112', 'N606+E112', 'N96+E112', 'N298+E112', 'N415+E81', 'N606+E81', 'N96+E81', 'N411+E81', 'N96+E52', 'N411+E52', 'N298+E52', 'N606+E52', 'N315+E171', 'N606+E171', 'N96+E171', 'N411+E171', 'N298+E162', 'N606+E162', 'N96+E162', 'N411+E162', 'N315+E13', 'N606+E13', 'N96+E13', 'N411+E13', 'N499+E187', 'N606+E187', 'N96+E187', 'N411+E187', 'N606+E137', 'N96+E137', 'N298+E137', 'N411+E137', 'N130+E191', 'N606+E191', 'N96+E191', 'N411+E191', 'N411+E189', 'N606+E189', 'N96+E189', 'N298+E189', 'N411+E204', 'N606+E204', 'N96+E204', 'N298+E204', 'N315+E92', 'N606+E92', 'N96+E92', 'N411+E92', 'N298+E69', 'N606+E69', 'N96+E69', 'N411+E69', 'N411+E160', 'N606+E160', 'N96+E160', 'N298+E160', 'N186+E175', 'N606+E175', 'N96+E175', 'N411+E175']

bcu: ['N642+E229', 'N672+E10', 'N293+E229', 'N26+E229', 'N64+E229', 'N293+E10', 'N228+E10', 'N293+E229', 'N69+E229', 'N204+E229', 'N281+E229', 'N293+E229', 'N293+E10', 'N372+E229', 'N204+E10', 'N362+E229', 'N672+E10', 'N293+E229', 'N281+E10', 'N656+E229', 'N642+E10', 'N64+E10', 'N281+E229', 'N293+E10', 'N642+E10', 'N293+E10', 'N189+E229', 'N228+E229', 'N64+E229', 'N228+E229', 'N262+E10', 'N672+E10', 'N672+E229', 'N204+E10', 'N293+E229', 'N204+E229', 'N293+E10', 'N281+E229', 'N26+E229', 'N189+E10', 'N664+E10', 'N228+E229', 'N281+E10', 'N64+E10', 'N212+E229', 'N64+E10', 'N362+E229', 'N637+E229', 'N141+E229', 'N189+E229', 'N293+E10', 'N637+E10', 'N587+E229', 'N35+E229', 'N228+E229', 'N626+E229', 'N189+E10', 'N64+E229', 'N629+E229', 'N664+E229', 'N84+E229', 'N281+E10', 'N204+E229', 'N204+E229', 'N204+E229', 'N281+E229', 'N656+E10', 'N637+E229', 'N26+E229', 'N281+E10', 'N587+E10', 'N199+E229', 'N64+E229', 'N141+E10', 'N204+E229', 'N212+E229', 'N204+E10', 'N642+E229', 'N189+E229', 'N587+E10', 'N281+E229', 'N293+E229', 'N226+E229',

'N626+E10', 'N637+E229', 'N212+E10', 'N212+E229', 'N293+E229', 'N637+E229', 'N587+E10', 'N672+E229', 'N204+E229', 'N587+E229', 'N149+E229', 'N281+E10', 'N262+E229', 'N281+E10', 'N293+E229', 'N431+E229', 'N204+E10']

fsc: ['N614+N214', 'N255+N483', 'N255+N457', 'N614+N208', 'N665+N392', 'N201+N678', 'N614+N392', 'N201+N208', 'N665+N483', 'N255+N208', 'N201+N274', 'N614+N570', 'N255+N162', 'N201+N560', 'N12+N678', 'N665+N680', 'N91+N678', 'N201+N214', 'N665+N162', 'N129+N678', 'N255+N559', 'N202+N208', 'N614+N151', 'N91+N483', 'N255+N560', 'N201+N151', 'N12+N690', 'N1+N208', 'N201+N222', 'N12+N521', 'N614+N283', 'N129+N680', 'N201+N559', 'N12+N214', 'N201+N613', 'N614+N341', 'N129+N570', 'N665+N167', 'N255+N614', 'N255+N614', 'N614+N255', 'N614+N255', 'N255+N665', 'N255+N665', 'N665+N255', 'N665+N255', 'N201+N614', 'N201+N614', 'N614+N201', 'N614+N201', 'N201+N665', 'N201+N665', 'N665+N201', 'N665+N201', 'N255+N201', 'N255+N201', 'N201+N255', 'N201+N255', 'N12+N614', 'N12+N614', 'N614+N12', 'N614+N12', 'N12+N665', 'N12+N665', 'N665+N12', 'N665+N12', 'N12+N201', 'N12+N201', 'N201+N12', 'N201+N12', 'N12+N255', 'N12+N255', 'N255+N12', 'N255+N12', 'N91+N614', 'N91+N614', 'N614+N91', 'N614+N91', 'N91+N665', 'N91+N665', 'N665+N91', 'N665+N91', 'N91+N201', 'N91+N201', 'N201+N91', 'N201+N91', 'N91+N255', 'N91+N255', 'N255+N91', 'N255+N91', 'N202+N614', 'N202+N614', 'N614+N202', 'N614+N202', 'N202+N665', 'N202+N665', 'N665+N202', 'N665+N202', 'N202+N201', 'N202+N201', 'N201+N202', 'N201+N202', 'N202+N255', 'N202+N255', 'N255+N202', 'N255+N202', 'N129+N614', 'N129+N614', 'N614+N129', 'N614+N129', 'N129+N665', 'N129+N665', 'N665+N129', 'N665+N129', 'N129+N201', 'N129+N201', 'N201+N129', 'N201+N129', 'N129+N255', 'N129+N255', 'N255+N129', 'N255+N129']

[Generator] Generate Structures:

pcu: 100%|██████████| 100/100 [00:44<00:00, 2.24it/s]

dia: 100%|██████████| 98/98 [01:08<00:00, 1.43it/s]

acs: 100%|██████████| 100/100 [00:41<00:00, 2.42it/s]

rtl: 100%|██████████| 106/106 [00:00<00:00, 2057.10it/s]

cds: 100%|██████████| 100/100 [00:20<00:00, 4.96it/s]

srs: 100%|██████████| 232/232 [01:34<00:00, 2.46it/s]

ths: 100%|██████████| 347/347 [02:34<00:00, 2.24it/s]

bcu: 100%|██████████| 100/100 [00:28<00:00, 3.53it/s]

fsc: 100%|██████████| 122/122 [00:22<00:00, 5.40it/s]

[Generator] Predict Properties: accessible_surface_area

load model : /home/dudgns1675/ChatMOF/chatmof/database/load_model/accessible_surface_area/best.ckpt

Predicting DataLoader 0: 100%|██████████| 7/7 [00:00<00:00, 14.08it/s]

load model : /home/dudgns1675/ChatMOF/chatmof/database/load_model/accessible_surface_area/best.ckpt

Predicting DataLoader 0: 100%|██████████| 6/6 [00:00<00:00, 11.38it/s]

load model : /home/dudgns1675/ChatMOF/chatmof/database/load_model/accessible_surface_area/best.ckpt

Predicting DataLoader 0: 100%|██████████| 5/5 [00:00<00:00, 11.73it/s]

load model : /home/dudgns1675/ChatMOF/chatmof/database/load_model/accessible_surface_area/best.ckpt

Predicting DataLoader 0: 100%|██████████| 3/3 [00:00<00:00, 9.74it/s]

load model : /home/dudgns1675/ChatMOF/chatmof/database/load_model/accessible_surface_area/best.ckpt

Predicting DataLoader 0: 100%|██████████| 9/9 [00:00<00:00, 12.69it/s]

load model : /home/dudgns1675/ChatMOF/chatmof/database/load_model/accessible_surface_area/best.ckpt

Predicting DataLoader 0: 100%|██████████| 4/4 [00:00<00:00, 11.45it/s]

load model : /home/dudgns1675/ChatMOF/chatmof/database/load_model/accessible_surface_area/best.ckpt

Predicting DataLoader 0: 100%|██████████| 3/3 [00:00<00:00, 9.13it/s]

load model : /home/dudgns1675/ChatMOF/chatmof/database/load_model/accessible_surface_area/best.ckpt

Predicting DataLoader 0: 100%|██████████| 3/3 [00:00<00:00, 9.69it/s]

[Generator] Run genetic algorithm : Cycle 3.

[Generator] Find Parents: extract cif_id and accessible_surface_area of 100 materials with the largest surface area from look-up tables.

> Entering new TableSearcher chain...

[Table Searcher] Thought: To extract the top 100 materials with the largest surface area, we can sort the DataFrame by the 'accessible_surface_area' column in descending order and then use the head() function to get the first 100 rows.

[Table Searcher] Input: import pandas as pd

import json

df_sorted = df.sort_values(by='accessible_surface_area', ascending=False)

print(df_sorted.head(100).to_markdown())

[Table Searcher] Observation:

	cif_id	accessible_surface_area
2957	pcu+N85+E33	5826.45
150	pcu+N47+E33	5808.59
2886	pcu+N613+E33	5728.23
2900	pcu+N483+E33	5723.77
2051	pcu+N274+E32	5723.77
2907	pcu+N613+E22	5696.98
2875	pcu+N483+E10	5692.52
2926	pcu+N559+E33	5683.59
2937	pcu+N234+E33	5679.12
2284	pcu+N234+E229	5665.73
2826	pcu+N613+E106	5661.26
2884	pcu+N613+E41	5656.8
419	pcu+N85+E77	5656.8
2853	pcu+N274+E33	5652.34
314	pcu+N694+E10	5647.87

```
|  861 | pcu+N559+E61  |                  5643.41 |
| 2525 | pcu+N613+E90  |                  5643.41 |
| 1547 | pcu+N47+E41   |                  5638.94 |
|   ...   |      .....             |                  ...         |
```

> Finished chain.

> Entering new TableSearcher chain...

[Table Searcher] Thought: To extract the top 100 materials with the largest surface area, we can sort the DataFrame by the 'accessible_surface_area' column in descending order and then use the head() function to get the top 100 rows.

[Table Searcher] Input: print(df.sort_values('accessible_surface_area', ascending=False).head(100).to_markdown())

[Table Searcher] Observation:

```
|      | cif_id        |   accessible_surface_area |
|-----:|:--------------|--------------------------:|
|  430 | dia+N515+E23  |                  6375.57 |
| 1238 | dia+N515+E24  |                  6362.17 |
| 2556 | dia+N267+E24  |                  6326.46 |
| 2613 | dia+N515+E3   |                  6308.6  |
| 2647 | dia+N515+E38  |                  6308.6  |
| 2715 | dia+N492+E36  |                  6304.14 |
| 2698 | dia+N76+E35   |                  6295.21 |
|  180 | dia+N310+E3   |                  6295.21 |
| 2062 | dia+N649+E47  |                  6290.74 |
| 2569 | dia+N697+E24  |                  6286.28 |
| 2688 | dia+N161+E24  |                  6268.42 |
| 2634 | dia+N515+E35  |                  6263.96 |
```

```
| 2706 | dia+N161+E3   |                  6263.96 |
| 2678 | dia+N515+E21  |                  6259.49 |
| 1807 | dia+N267+E23  |                  6250.56 |
| 2674 | dia+N76+E34   |                  6250.56 |
| 2566 | dia+N310+E24  |                  6250.56 |
| 1265 | dia+N310+E47  |                  6250.56 |
| 2598 | dia+N76+E23   |                  6246.1  |
||   ...  |      .....             |                  ...         |
```

> Finished chain.

> Entering new TableSearcher chain...

[Table Searcher] Thought: To extract the top 100 materials with the largest surface area, we can sort the DataFrame by the 'accessible_surface_area' column in descending order and then use the head() function to get the first 100 rows.

[Table Searcher] Input: import pandas as pd

```
import json
df_sorted = df.sort_values(by='accessible_surface_area', ascending=False)
print(df_sorted.head(100).to_markdown())
```

[Table Searcher] Observation:

```
|      | cif_id        |   accessible_surface_area |
|-----:|:--------------|--------------------------:|
| 2555 | acs+N628+E39  |                  6228.24 |
| 2538 | acs+N574+E10  |                  6143.42 |
| 2559 | acs+N553+E10  |                  6121.1  |
| 2633 | acs+N553+E10  |                  6103.24 |
| 2568 | acs+N198+E10  |                  6080.92 |
```

```
| 2569 | acs+N198+E39  |                6045.2  |
| 2645 | acs+N198+E39  |                6040.74 |
| 1247 | acs+N489+E10  |                6000.56 |
| 1747 | acs+N553+E10  |                5978.24 |
|  469 | acs+N481+E10  |                5960.38 |
| 2552 | acs+N553+E39  |                5880.02 |
| 2591 | acs+N481+E39  |                5880.02 |
| 2536 | acs+N489+E10  |                5875.56 |
| 2514 | acs+N628+E10  |                5875.56 |
| 1781 | acs+N628+E194 |                5871.09 |
||  ...  |     .....           |                  ...        |
```

> Finished chain.

> Entering new TableSearcher chain...

[Table Searcher] Thought: To extract the top 100 materials with the largest surface area, we need to sort the DataFrame by the 'accessible_surface_area' column in descending order and then use the head() function to get the top 100 rows.

[Table Searcher] Input: import pandas as pd

```
import json
df_sorted = df.sort_values(by='accessible_surface_area', ascending=False)
print(df_sorted.head(100).to_markdown())
```

[Table Searcher] Observation:

```
|      | cif_id        |   accessible_surface_area |
|-----:|:--------------|--------------------------:|
|  686 | rtl+N535+N234 |                6411.28 |
| 2400 | rtl+N535+N392 |                6375.57 |
```

```
|  991 | rtl+N535+N222 |                  6313.06 |
| 2395 | rtl+N535+N234 |                  6313.06 |
| 1503 | rtl+N535+N680 |                  6259.49 |
| 1399 | rtl+N535+N162 |                  6259.49 |
|  497 | rtl+N535+N613 |                  6255.03 |
|  321 | rtl+N535+N668 |                  6250.56 |
| 2361 | rtl+N535+N213 |                  6241.63 |
| 1288 | rtl+N535+N483 |                  6232.71 |
| 2386 | rtl+N535+N613 |                  6228.24 |
| 2360 | rtl+N535+N465 |                  6223.78 |
| 2405 | rtl+N535+N222 |                  6188.06 |
|  405 | rtl+N535+N106 |                  6183.6  |
|  701 | rtl+N535+N598 |                  6170.2  |
|   28 | rtl+N535+N590 |                  6161.28 |
|  747 | rtl+N535+N289 |                  6161.28 |
|  205 | rtl+N535+N283 |                  6147.88 |
| 2445 | rtl+N535+N678 |                  6147.88 |
|  195 | rtl+N535+N521 |                  6138.95 |
| 1405 | rtl+N535+N559 |                  6138.95 |
| 2418 | rtl+N535+N559 |                  6125.56 |
| 1377 | rtl+N535+N341 |                  6125.56 |
| 1907 | rtl+N535+N696 |                  6121.1  |
| 2410 | rtl+N535+N16  |                  6098.77 |
| 1605 | rtl+N535+N309 |                  6089.85 |
|  ...  |     .....         |                   ...        |
> Finished chain.
```

> Entering new TableSearcher chain...

[Table Searcher] Thought: To extract the top 100 materials with the largest surface area, we can sort the DataFrame by the 'accessible_surface_area' column in descending order and then use the head() function to get the top 100 rows.

[Table Searcher] Input: print(df.sort_values(by='accessible_surface_area', ascending=False).head(100).to_markdown())

[Table Searcher] Observation:

	cif_id	accessible_surface_area
2720	cds+N259+E224	5964.84
2803	cds+N654+E32	5951.45
2396	cds+N112+E32	5933.59
2774	cds+N292+E32	5929.13
2417	cds+N654+E32	5929.13
2737	cds+N464+E224	5920.2
508	cds+N561+E32	5915.73
2796	cds+N194+E224	5906.81
2765	cds+N112+E32	5906.81
2746	cds+N194+E224	5902.34
2740	cds+N500+E32	5897.88
2768	cds+N500+E33	5893.41
2783	cds+N512+E32	5875.56
2725	cds+N512+E32	5862.16
1395	cds+N464+E224	5862.16
959	cds+N464+E234	5853.23
2742	cds+N194+E32	5853.23
2718	cds+N654+E33	5848.77
2794	cds+N194+E32	5844.3

```
| 1369 | cds+N292+E176 |                  5839.84 |
| 1037 | cds+N470+E7   |                  5839.84 |
| 2775 | cds+N193+E32  |                  5830.91 |
| 2699 | cds+N561+E110 |                  5830.91 |
| 2749 | cds+N551+E32  |                  5830.91 |
| 2772 | cds+N549+E32  |                  5826.45 |
| 1063 | cds+N193+E41  |                  5826.45 |
|  ...  |      .....            |                   ...         |
```

> Finished chain.

> Entering new TableSearcher chain...

[Table Searcher] Thought: To extract the top 100 materials with the largest surface area, we need to sort the DataFrame by the 'accessible_surface_area' column in descending order and then use the head() function to get the first 100 rows.

[Table Searcher] Input: import pandas as pd

```
import json
df_sorted = df.sort_values(by='accessible_surface_area', ascending=False)
print(df_sorted.head(100).to_markdown())
```

[Table Searcher] Observation:

```
|      | cif_id        |   accessible_surface_area |
|-----:|:--------------|--------------------------:|
| 2455 | srs+N611+E29  |                   6147.88 |
| 2412 | srs+N611+E25  |                   6112.17 |
| 2517 | srs+N606+E14  |                   6098.77 |
| 2406 | srs+N611+E16  |                   6067.52 |
|  235 | srs+N285+E129 |                   6063.06 |
```

```
|  723 | srs+N298+E63  |                 6058.6  |
| 2429 | srs+N606+E85  |                 6058.6  |
| 2112 | srs+N606+E45  |                 6058.6  |
|  278 | srs+N298+E128 |                 6054.13 |
| 2478 | srs+N606+E220 |                 6049.67 |
|  319 | srs+N285+E172 |                 6049.67 |
| 1110 | srs+N298+E168 |                 6045.2  |
|  ...  |      .....          |               ...        |
> Finished chain.



> Entering new TableSearcher chain...


[Table Searcher] Thought: To extract the top 100 materials with the largest surface area, we can sort the DataFrame by the 'accessible_surface_area' column in descending order and then use the head() function to get the top 100 rows.
[Table Searcher] Input: import pandas as pd
import json
print(df.sort_values('accessible_surface_area', ascending=False).head(100).to_markdown())
[Table Searcher] Observation:
|      | cif_id        |   accessible_surface_area |
|-----:|:--------------|--------------------------:|
| 1274 | ths+N606+E8   |                   5853.23 |
|  193 | ths+N298+E81  |                   5835.38 |
|  605 | ths+N96+E149  |                   5835.38 |
| 1151 | ths+N606+E11  |                   5830.91 |
|  597 | ths+N606+E23  |                   5830.91 |
|  147 | ths+N411+E27  |                   5821.98 |
```

```
| 1284 | ths+N411+E44  |                 5821.98 |
| 1303 | ths+N411+E27  |                 5817.52 |
| 1307 | ths+N411+E44  |                 5813.05 |
|  293 | ths+N411+E44  |                 5808.59 |
|  506 | ths+N298+E168 |                 5804.12 |
|  632 | ths+N606+E156 |                 5804.12 |
|  499 | ths+N606+E24  |                 5795.2  |
| 1328 | ths+N315+E128 |                 5795.2  |
|  494 | ths+N298+E167 |                 5795.2  |
| 1185 | ths+N606+E26  |                 5795.2  |
|  861 | ths+N606+E195 |                 5786.27 |
|  403 | ths+N606+E219 |                 5772.87 |
| 1251 | ths+N96+E19   |                 5772.87 |
| 1258 | ths+N411+E13  |                 5750.55 |
|  508 | ths+N606+E112 |                 5750.55 |
|  735 | ths+N411+E8   |                 5750.55 |
|  527 | ths+N96+E15   |                 5746.09 |
||  ...  |   .....       |        ...       |
```

> Finished chain.

> Entering new TableSearcher chain...

[Table Searcher] Thought: To extract the top 100 materials with the largest surface area, we can sort the DataFrame by the 'accessible_surface_area' column in descending order and then use the head() function to get the top 100 rows. We can then select the 'cif_id' and 'accessible_surface_area' columns.

[Table Searcher] Input: import pandas as pd

import json

```
print(df.sort_values('accessible_surface_area', ascending=False).head(100)[['cif_id', 'accessible_surface_area']].to_markdown())
```

[Table Searcher] Observation:

```
|      | cif_id        |   accessible_surface_area |
|-----:|:--------------|--------------------------:|
| 2794 | bcu+N642+E10  |                   6219.31 |
| 2809 | bcu+N672+E10  |                   6156.81 |
|    3 | bcu+N672+E229 |                   6152.35 |
| 2813 | bcu+N293+E10  |                   6121.1  |
| 2757 | bcu+N293+E10  |                   6112.17 |
| 2796 | bcu+N26+E10   |                   6080.92 |
| 2826 | bcu+N64+E10   |                   6071.99 |
| 2775 | bcu+N64+E10   |                   6067.52 |
| 1191 | bcu+N293+E39  |                   6040.74 |
| 2768 | bcu+N228+E229 |                   6027.34 |
| 2820 | bcu+N228+E229 |                   6013.95 |
| 2762 | bcu+N293+E1   |                   6009.49 |
| 2758 | bcu+N69+E10   |                   5955.91 |
| 1097 | bcu+N204+E10  |                   5951.45 |
| 2779 | bcu+N281+E10  |                   5929.13 |
|  438 | bcu+N293+E1   |                   5911.27 |
|  403 | bcu+N372+E10  |                   5902.34 |
| 1740 | bcu+N293+E121 |                   5902.34 |
| 1652 | bcu+N204+E39  |                   5897.88 |
| 2801 | bcu+N362+E10  |                   5897.88 |
| 2753 | bcu+N672+E229 |                   5884.48 |
|   21 | bcu+N293+E36  |                   5884.48 |
| 2756 | bcu+N281+E229 |                   5844.3  |
```

```
| 2812 | bcu+N281+E229 |                5839.84 |
| 2836 | bcu+N262+E10  |                5817.52 |
| 1389 | bcu+N656+E10  |                5746.09 |
| 1720 | bcu+N642+E229 |                5732.69 |
|  492 | bcu+N64+E225  |                5728.23 |
| 2828 | bcu+N26+E229  |                5723.77 |
|  ...  |     .....          |                 ...        |
```

> Finished chain.

> Entering new TableSearcher chain...

[Table Searcher] Thought: To extract the top 100 materials with the largest surface area, we can sort the DataFrame by the 'accessible_surface_area' column in descending order and then use the head() function to get the top 100 rows.

[Table Searcher] Input: import pandas as pd

```
import json
df_sorted = df.sort_values(by='accessible_surface_area', ascending=False)
print(df_sorted.head(100).to_markdown())
```

[Table Searcher] Observation:

```
|      | cif_id        |   accessible_surface_area |
|-----:|:--------------|--------------------------:|
|   35 | fsc+N614+N483 |                   4279.54 |
| 1751 | fsc+N255+N214 |                   4266.14 |
| 1768 | fsc+N255+N483 |                   4266.14 |
| 1511 | fsc+N255+N214 |                   4263.91 |
| 1731 | fsc+N614+N457 |                   4243.82 |
|  342 | fsc+N614+N457 |                   4241.59 |
```

```
| 1750 | fsc+N614+N208 |          4176.86 |
| 1735 | fsc+N665+N392 |          4174.62 |
| 1767 | fsc+N665+N392 |          4167.93 |
| 1788 | fsc+N614+N208 |          4165.7  |
|  621 | fsc+N201+N457 |          4159    |
|  885 | fsc+N614+N678 |          4154.54 |
| 1753 | fsc+N665+N457 |          4083.1  |
| 1220 | fsc+N201+N483 |          4049.62 |
| 1741 | fsc+N614+N392 |          4049.62 |
| 1524 | fsc+N201+N208 |          4042.93 |
| 1747 | fsc+N665+N208 |          4040.69 |
| 1777 | fsc+N614+N392 |          4040.69 |
|  420 | fsc+N255+N392 |          4022.84 |
|  ...  |     .....      |          ...     |
> Finished chain.
```

[Generator] Get Children: generate 100 new materials with the largest surface area from the 100 materials.

acs: ['N85+E32', 'N47+E32', 'N613+E32', 'N483+E32', 'N274+E33', 'N613+E33', 'N483+E33', 'N559+E32', 'N234+E32', 'N234+E33', 'N613+E33', 'N613+E32', 'N85+E32', 'N274+E32', 'N694+E33', 'N559+E33', 'N613+E32', 'N47+E32', 'N613+E33', 'N574+E33', 'N613+E32', 'N295+E33', 'N668+E32', 'N47+E33', 'N628+E33', 'N613+E32', 'N559+E32', 'N678+E32', 'N613+E33', 'N680+E33', 'N406+E32', 'N341+E33', 'N274+E33', 'N483+E33', 'N274+E33', 'N613+E33', 'N680+E33', 'N47+E33', 'N213+E33', 'N295+E33', 'N694+E32', 'N483+E33', 'N180+E32', 'N274+E33', 'N47+E33', 'N680+E32', 'N559+E33', 'N222+E33', 'N222+E32', 'N222+E33', 'N47+E33', 'N180+E33', 'N47+E33', 'N680+E33', 'N392+E33', 'N16+E32', 'N595+E33', 'N483+E33', 'N222+E33', 'N483+E33', 'N167+E32', 'N540+E33', 'N180+E33', 'N222+E33', 'N559+E33', 'N213+E33', 'N47+E33', 'N657+E33', 'N222+E33', 'N657+E33', 'N274+E33', 'N222+E33', 'N47+E33', 'N483+E33', 'N240+E33', 'N392+E33', 'N392+E33', 'N47+E33', 'N222+E33', 'N392+E33', 'N559+E33', 'N392+E33', 'N598+E33', 'N234+E33', 'N678+E33', 'N483+E33', 'N180+E33', 'N392+E33', 'N598+E33', 'N162+E33', 'N47+E33', 'N392+E33', 'N274+E33', 'N483+E33', 'N148+E33', 'N483+E33', 'N680+E33', 'N222+E33', 'N214+E33', 'N483+E33']

dia: ['N515+E35', 'N267+E38', 'N492+E23', 'N76+E24', 'N310+E3', 'N649+E24', 'N697+E3', 'N161+E38', 'N515+E47', 'N161+E3', 'N267+E21', 'N76+E35', 'N310+E24', 'N649+E35', 'N697+E47', 'N161+E21', 'N515+E34', 'N267+E35', 'N76+E24', 'N310+E47', 'N649+E34', 'N697+E23', 'N161+E35', 'N515+E21', 'N267+E34', 'N76+E23', 'N310+E23', 'N649+E23', 'N697+E21', 'N161+E24', 'N515+E35', 'N267+E23', 'N76+E3', 'N310+E35', 'N649+E3', 'N697+E35', 'N161+E3', 'N515+E24', 'N267+E3', 'N76+E47', 'N310+E34', 'N649+E47', 'N697+E34', 'N161+E47', 'N515+E47', 'N267+E47', 'N76+E21', 'N310+E21', 'N649+E21', 'N697+E21', 'N161+E21', 'N515+E23', 'N267+E24', 'N76+E35', 'N310+E35', 'N649+E35', 'N697+E35', 'N161+E35', 'N515+E34', 'N267+E34', 'N76+E34', 'N310+E34', 'N649+E34', 'N697+E34', 'N161+E34', 'N515+E21', 'N267+E21', 'N76+E21', 'N310+E21', 'N649+E21', 'N697+E21', 'N161+E21', 'N515+E35', 'N267+E35', 'N76+E35', 'N310+E35', 'N649+E35', 'N697+E35', 'N161+E35', 'N515+E24', 'N267+E24', 'N76+E24', 'N310+E24', 'N649+E24', 'N697+E24', 'N161+E24', 'N515+E3', 'N267+E3', 'N76+E3', 'N310+E3', 'N649+E3', 'N697+E3', 'N161+E3', 'N515+E47', 'N267+E47', 'N76+E47', 'N310+E47', 'N649+E47', 'N697+E47', 'N161+E47', 'N515+E34', 'N267+E34', 'N76+E34', 'N310+E34', 'N649+E34', 'N697+E34', 'N161+E34', 'N515+E21', 'N267+E21', 'N76+E21', 'N310+E21', 'N649+E21', 'N697+E21', 'N161+E21']

acs: ['N628+E10', 'N628+E9', 'N628+E1', 'N628+E4', 'N628+E7', 'N628+E22', 'N628+E28', 'N628+E33', 'N628+E36', 'N628+E59', 'N628+E121', 'N628+E194', 'N628+E225', 'N628+E229', 'N574+E39', 'N574+E10', 'N574+E9', 'N574+E1', 'N574+E4', 'N574+E7', 'N574+E33', 'N574+E36', 'N574+E121', 'N574+E194', 'N574+E225', 'N574+E229', 'N553+E39', 'N553+E10', 'N553+E9', 'N553+E1', 'N553+E4', 'N553+E7', 'N553+E22', 'N553+E28', 'N553+E33', 'N553+E36', 'N553+E59', 'N553+E121', 'N553+E194', 'N553+E225', 'N553+E229', 'N198+E39', 'N198+E10', 'N198+E9', 'N198+E1', 'N198+E4', 'N198+E7', 'N198+E22', 'N198+E28', 'N198+E33', 'N198+E36', 'N198+E59', 'N198+E121', 'N198+E194', 'N198+E225', 'N198+E229', 'N489+E39', 'N489+E10', 'N489+E9', 'N489+E1', 'N489+E4', 'N489+E7', 'N489+E22', 'N489+E28', 'N489+E33', 'N489+E36', 'N489+E59', 'N489+E121', 'N489+E194', 'N489+E225', 'N489+E229', 'N481+E39', 'N481+E10', 'N481+E9', 'N481+E1', 'N481+E4', 'N481+E7', 'N481+E22', 'N481+E28', 'N481+E33', 'N481+E36', 'N481+E59', 'N481+E121', 'N481+E194', 'N481+E225', 'N481+E229', 'N125+E39', 'N125+E10', 'N125+E9', 'N125+E1', 'N125+E4', 'N125+E7', 'N125+E22', 'N125+E28', 'N125+E33', 'N125+E36', 'N125+E59', 'N125+E121', 'N125+E194', 'N125+E225', 'N125+E229']

rtl: ['N234+N392', 'N234+N222', 'N234+N680', 'N234+N162', 'N234+N613', 'N234+N668', 'N234+N213', 'N234+N483', 'N234+N465', 'N234+N106', 'N234+N598', 'N234+N590', 'N234+N289', 'N234+N283', 'N234+N678', 'N234+N521', 'N234+N559', 'N234+N341', 'N234+N696', 'N234+N16', 'N234+N309', 'N234+N151', 'N234+N593', 'N234+N457', 'N234+N336', 'N234+N148', 'N234+N214', 'N234+N504', 'N234+N36', 'N234+N569', 'N234+N508', 'N234+N480', 'N234+N311', 'N234+N33', 'N234+N447', 'N234+N135', 'N234+N205', 'N234+N105', 'N234+N630', 'N234+N453', 'N234+N529', 'N234+N491', 'N234+N65', 'N234+N146', 'N234+N518', 'N234+N366', 'N234+N250', 'N234+N324', 'N234+N555', 'N234+N486', 'N535+N392', 'N535+N680', 'N535+N162', 'N535+N668', 'N535+N213', 'N535+N483', 'N535+N465', 'N535+N598', 'N535+N590', 'N535+N289',

'N535+N283', 'N535+N521', 'N535+N341', 'N535+N696', 'N535+N16', 'N535+N309', 'N535+N593', 'N535+N457', 'N535+N336', 'N535+N148', 'N535+N214', 'N535+N569', 'N535+N508', 'N535+N480', 'N535+N311', 'N535+N33', 'N535+N447', 'N535+N135', 'N535+N205', 'N535+N105', 'N535+N630', 'N535+N453', 'N535+N529', 'N535+N491', 'N535+N65', 'N535+N146', 'N535+N518', 'N535+N366', 'N535+N250', 'N535+N324', 'N535+N555', 'N535+N486', 'N535+N442']

cds: ['N259+E32', 'N654+E224', 'N112+E259', 'N292+E654', 'N654+E112', 'N464+E292', 'N561+E654', 'N194+E464', 'N112+E561', 'N194+E194', 'N500+E112', 'N500+E194', 'N512+E500', 'N512+E500', 'N464+E512', 'N464+E512', 'N194+E464', 'N654+E464', 'N194+E654', 'N292+E194', 'N470+E654', 'N193+E292', 'N561+E470', 'N551+E193', 'N549+E561', 'N193+E551', 'N211+E549', 'N112+E193', 'N193+E211', 'N112+E112', 'N551+E112', 'N551+E193', 'N259+E551', 'N500+E259', 'N512+E500', 'N503+E512', 'N200+E503', 'N193+E200', 'N194+E193', 'N193+E194', 'N259+E193', 'N335+E259', 'N464+E335', 'N689+E464', 'N464+E689', 'N259+E464', 'N689+E259', 'N194+E689', 'N409+E194', 'N500+E409', 'N551+E500', 'N551+E551', 'N557+E551', 'N27+E557', 'N365+E27', 'N464+E365', 'N211+E464', 'N500+E211', 'N194+E500', 'N365+E194', 'N464+E365', 'N259+E464', 'N365+E259', 'N445+E365', 'N557+E445', 'N689+E557', 'N561+E689', 'N259+E561', 'N618+E259', 'N503+E618', 'N194+E503', 'N464+E194', 'N500+E464', 'N654+E500', 'N342+E654', 'N335+E342', 'N355+E335', 'N500+E355', 'N654+E500', 'N464+E654', 'N618+E464', 'N211+E618', 'N174+E211', 'N335+E174', 'N194+E335', 'N512+E194', 'N365+E512', 'N561+E365', 'N200+E561', 'N302+E200', 'N654+E302', 'N259+E654', 'N112+E259', 'N464+E112', 'N194+E464', 'N193+E194', 'N292+E193', 'N654+E292', 'N549+E654', 'N500+E549']

srs: ['N611+E14', 'N611+E85', 'N611+E45', 'N611+E128', 'N611+E220', 'N611+E172', 'N611+E168', 'N611+E212', 'N611+E58', 'N611+E180', 'N611+E138', 'N611+E8', 'N611+E71', 'N611+E151', 'N611+E37', 'N611+E19', 'N611+E48', 'N611+E132', 'N611+E173', 'N611+E119', 'N611+E216', 'N611+E72', 'N611+E50', 'N611+E18', 'N611+E205', 'N611+E105', 'N611+E16', 'N611+E166', 'N611+E188', 'N611+E221', 'N611+E204', 'N611+E146', 'N611+E167', 'N611+E130', 'N611+E67', 'N611+E186', 'N611+E66', 'N611+E201', 'N611+E159', 'N611+E139', 'N611+E81', 'N611+E206', 'N611+E11', 'N611+E160', 'N611+E19', 'N611+E182', 'N611+E108', 'N611+E27', 'N611+E226', 'N611+E175', 'N611+E37', 'N611+E149', 'N611+E52', 'N611+E220', 'N611+E159', 'N285+E14', 'N285+E85', 'N285+E45', 'N285+E128', 'N285+E220', 'N285+E172', 'N285+E168', 'N285+E212', 'N285+E58', 'N285+E180', 'N285+E138', 'N285+E8', 'N285+E71', 'N285+E151', 'N285+E37', 'N285+E19', 'N285+E48', 'N285+E132', 'N285+E173', 'N285+E119', 'N285+E216', 'N285+E72', 'N285+E50', 'N285+E18', 'N285+E205', 'N285+E105', 'N285+E16', 'N285+E166', 'N285+E188', 'N285+E221', 'N285+E204', 'N285+E146', 'N285+E167', 'N285+E130', 'N285+E67', 'N285+E186', 'N285+E66', 'N285+E201', 'N285+E159', 'N285+E139', 'N285+E81', 'N285+E206', 'N285+E11', 'N285+E160', 'N285+E19', 'N285+E182', 'N285+E108', 'N285+E27', 'N285+E226', 'N285+E175', 'N285+E37', 'N285+E149', 'N285+E52', 'N285+E220', 'N285+E159']

ths: ['N606+E81', 'N298+E8', 'N96+E8', 'N606+E149', 'N411+E8', 'N411+E149', 'N298+E149', 'N96+E11', 'N606+E23', 'N411+E23', 'N298+E23', 'N96+E27', 'N606+E44', 'N411+E44', 'N298+E44', 'N96+E156', 'N606+E168', 'N411+E168', 'N298+E168', 'N96+E24', 'N606+E128', 'N411+E128', 'N298+E128', 'N96+E167', 'N606+E26', 'N411+E26', 'N298+E26', 'N96+E195', 'N606+E219', 'N411+E219', 'N298+E219', 'N96+E19', 'N606+E13', 'N411+E13', 'N298+E13', 'N96+E112', 'N606+E8', 'N411+E8', 'N298+E8', 'N96+E15', 'N606+E216', 'N411+E216', 'N298+E216', 'N96+E72', 'N606+E76', 'N411+E76', 'N298+E76', 'N96+E136', 'N606+E16', 'N411+E16', 'N298+E16', 'N96+E57', 'N606+E27', 'N411+E27', 'N298+E27', 'N96+E220', 'N606+E182', 'N411+E182', 'N298+E182', 'N96+E171', 'N606+E187', 'N411+E187', 'N298+E187', 'N96+E18', 'N606+E15', 'N411+E15', 'N298+E15', 'N96+E158', 'N606+E108', 'N411+E108', 'N298+E108', 'N96+E207', 'N606+E95', 'N411+E95', 'N298+E95', 'N96+E130', 'N606+E181', 'N411+E181', 'N298+E181', 'N96+E148', 'N606+E45', 'N411+E45', 'N298+E45', 'N96+E201', 'N606+E234', 'N411+E234', 'N298+E234', 'N96+E138', 'N606+E146', 'N411+E146', 'N298+E146', 'N96+E43', 'N606+E159', 'N411+E159', 'N298+E159', 'N96+E86', 'N606+E76', 'N411+E76', 'N298+E76', 'N96+E71', 'N606+E132', 'N411+E132', 'N298+E132', 'N96+E44', 'N606+E190', 'N411+E190', 'N298+E190', 'N96+E34', 'N606+E120', 'N411+E120', 'N298+E120', 'N96+E178', 'N606+E151', 'N411+E151', 'N298+E151', 'N96+E48', 'N606+E186', 'N411+E186', 'N298+E186', 'N96+E16', 'N606+E150', 'N411+E150', 'N298+E150', 'N96+E113', 'N606+E175', 'N411+E175', 'N298+E175', 'N96+E131', 'N606+E195', 'N411+E195', 'N298+E195', 'N96+E86', 'N606+E81', 'N411+E81', 'N298+E81', 'N96+E52', 'N606+E112', 'N411+E112', 'N298+E112', 'N96+E13', 'N606+E162', 'N411+E162', 'N298+E162', 'N96+E52']

bcu: ['N642+E39', 'N672+E36', 'N293+E229', 'N26+E229', 'N64+E229', 'N372+E229', 'N204+E229', 'N362+E229', 'N672+E1', 'N281+E36', 'N262+E229', 'N656+E229', 'N26+E1', 'N189+E229', 'N228+E10', 'N212+E229', 'N672+E121', 'N204+E1', 'N362+E1', 'N293+E10', 'N281+E1', 'N262+E1', 'N656+E1', 'N642+E1', 'N189+E1', 'N228+E1', 'N212+E1', 'N672+E10', 'N204+E10', 'N362+E10', 'N293+E229', 'N281+E229', 'N262+E229', 'N656+E229', 'N642+E229', 'N189+E229', 'N228+E229', 'N212+E229', 'N672+E229', 'N204+E229', 'N362+E229', 'N293+E1', 'N281+E1', 'N262+E1', 'N656+E1', 'N642+E1', 'N189+E1', 'N228+E1', 'N212+E1', 'N672+E1', 'N204+E1', 'N362+E1', 'N293+E39', 'N281+E39', 'N262+E39', 'N656+E39', 'N642+E39', 'N189+E39', 'N228+E39', 'N212+E39', 'N672+E39', 'N204+E39', 'N362+E39', 'N293+E59', 'N281+E59', 'N262+E59', 'N656+E59', 'N642+E59', 'N189+E59', 'N228+E59', 'N212+E59', 'N672+E59', 'N204+E59', 'N362+E59', 'N293+E225', 'N281+E225', 'N262+E225', 'N656+E225', 'N642+E225', 'N189+E225', 'N228+E225', 'N212+E225', 'N672+E225', 'N204+E225', 'N362+E225', 'N293+E223', 'N281+E223', 'N262+E223', 'N656+E223', 'N642+E223', 'N189+E223', 'N228+E223', 'N212+E223', 'N672+E223', 'N204+E223', 'N362+E223']

fsc: ['N614+N214', 'N255+N483', 'N665+N392', 'N201+N457', 'N12+N208', 'N255+N678', 'N614+N274', 'N201+N613', 'N91+N678', 'N255+N162', 'N12+N678', 'N665+N214', 'N255+N680', 'N91+N457', 'N201+N570', 'N91+N678', 'N665+N274', 'N614+N162', 'N129+N678', 'N255+N55', 'N202+N457', 'N614+N151', 'N91+N392', 'N255+N559', 'N91+N483', 'N202+N208', 'N614+N167', 'N399+N457', 'N255+N33', 'N201+N151',

'N12+N274', 'N255+N690', 'N614+N283', 'N129+N214', 'N1+N457', 'N255+N214', 'N665+N457', 'N201+N483', 'N614+N392', 'N255+N392', 'N665+N483', 'N12+N208', 'N255+N457', 'N614+N680', 'N201+N678', 'N255+N208', 'N12+N392', 'N614+N570', 'N201+N274', 'N614+N560', 'N255+N560', 'N201+N214', 'N614+N678', 'N614+N570', 'N255+N162', 'N202+N208', 'N614+N151', 'N91+N483', 'N202+N208', 'N614+N151', 'N91+N483', 'N255+N560', 'N201+N560', 'N12+N274', 'N665+N680', 'N1+N457', 'N665+N214', 'N201+N483', 'N614+N392', 'N255+N392', 'N665+N483', 'N12+N208', 'N255+N457', 'N614+N680', 'N201+N678', 'N255+N208', 'N12+N392', 'N614+N570', 'N201+N274', 'N614+N560', 'N255+N560', 'N201+N214', 'N614+N678', 'N614+N570', 'N255+N162', 'N202+N208', 'N614+N151', 'N91+N483', 'N202+N208', 'N614+N151', 'N91+N483', 'N255+N560', 'N201+N560', 'N12+N274', 'N665+N680', 'N1+N457']

[Generator] Generate Structures:

pcu: 100%|██████████| 100/100 [00:18<00:00, 5.35it/s]

dia: 100%|██████████| 114/114 [00:37<00:00, 3.01it/s]

acs: 100%|██████████| 101/101 [00:55<00:00, 1.83it/s]

rtl: 100%|██████████| 93/93 [00:28<00:00, 3.22it/s]

cds: 100%|██████████| 100/100 [00:13<00:00, 7.20it/s]

srs: 100%|██████████| 110/110 [00:33<00:00, 3.33it/s]

ths: 100%|██████████| 144/144 [01:09<00:00, 2.08it/s]

bcu: 100%|██████████| 96/96 [00:57<00:00, 1.67it/s]

fsc: 100%|██████████| 96/96 [00:33<00:00, 2.90it/s]

[Generator] Predict Properties: accessible_surface_area

load model : /home/dudgns1675/ChatMOF/chatmof/database/load_model/accessible_surface_area/best.ckpt

Predicting DataLoader 0: 100%|██████████| 3/3 [00:00<00:00, 9.43it/s]

load model : /home/dudgns1675/ChatMOF/chatmof/database/load_model/accessible_surface_area/best.ckpt

Predicting DataLoader 0: 100%|██████████| 4/4 [00:00<00:00, 10.84it/s]

load model : /home/dudgns1675/ChatMOF/chatmof/database/load_model/accessible_surface_area/best.ckpt

Predicting DataLoader 0: 100%|██████████| 7/7 [00:00<00:00, 12.82it/s]

load model : /home/dudgns1675/ChatMOF/chatmof/database/load_model/accessible_surface_area/best.ckpt

Predicting DataLoader 0: 100%|██████████| 3/3 [00:00<00:00, 7.89it/s]

load model : /home/dudgns1675/ChatMOF/chatmof/database/load_model/accessible_surface_area/best.ckpt

Predicting DataLoader 0: 100%|██████████| 2/2 [00:00<00:00, 7.92it/s]

load model : /home/dudgns1675/ChatMOF/chatmof/database/load_model/accessible_surface_area/best.ckpt

Predicting DataLoader 0: 100%|██████████| 4/4 [00:00<00:00, 10.65it/s]

load model : /home/dudgns1675/ChatMOF/chatmof/database/load_model/accessible_surface_area/best.ckpt

Predicting DataLoader 0: 100%|██████████| 2/2 [00:00<00:00, 7.84it/s]

load model : /home/dudgns1675/ChatMOF/chatmof/database/load_model/accessible_surface_area/best.ckpt

Predicting DataLoader 0: 100%|██████████| 5/5 [00:00<00:00, 10.66it/s]

load model : /home/dudgns1675/ChatMOF/chatmof/database/load_model/accessible_surface_area/best.ckpt

Predicting DataLoader 0: 100%|██████████| 4/4 [00:00<00:00, 11.35it/s]

[Generator] Final Thought: Based on the generated CIF, find the material that has the largest surface area.

> Entering new TableSearcher chain...

[Table Searcher] Thought: To find the material with the largest surface area, we can use the max() function in pandas on the 'accessible_surface_area' column. However, we also need to know the corresponding 'cif_id' for that maximum surface area. Therefore, we should use the idxmax() function which returns the index of the first occurrence of maximum over requested axis.

[Table Searcher] Input: max_surface_area_material = df['accessible_surface_area'].idxmax()

print(df.loc[max_surface_area_material].to_markdown())

[Table Searcher] Observation:

	9193
cif_id	rtl+N535+N234
accessible_surface_area	6411.28173828125

[Table Searcher] Final Thought: The material with the largest surface area is 'rtl+N535+N234' with a surface area of 6411.28173828125 m^2/g.

> Finished chain.

> Finished chain.

Observation: The answer for question "Based on the generated CIF, find the material that has the largest surface area." is 'rtl+N535+N234'.

Thought:I now know the final answer

Final Answer: The structure with the largest surface area is 'rtl+N535+N234'.

> Finished chain.

########## Output ################################

The structure with the largest surface area is 'rtl+N535+N234'.

Thanks for using CHATMOF!

> Finished chain.

Supplementary Note S2. Question generation prompts for search and prediction tasks

I have a model that generates the structures with specific properties, and you need to generate a question to match. The question should be as diverse as possible, and should be something a human would ask the model. The property should be one of [bandgap, hydrogen uptake (100bar), hydrogen diffusivity, O2 diffusivity, N2 diffusivity, O2 uptake, N2 uptake, thermal stability, CO2 henry coefficient, surface area, largest cavity diameter, pore limiting diameter, pore volume, volume fraction]. You add temperature 298K and 1 bar condition for each question with property listed in ['diffusivity', 'uptake', and 'henry coefficient'].<br><br>Example:<br>- What are the top 10 materials with the highest bandgap?<br>- What is the bandgap of [mat]?<br>- Can you show the materials with a hydrogen uptake greater than [value] at 298K, 1 bar?<br><br>Generate questions.<br><br>Begin!

After creating the prompt, select the CoreMOF refcode and value for the property in the [mat] and [value] fields. For 298K and 1 bar, select the appropriate temperature and pressure for the property. Among the properties, those that can be obtained from the look-up table are classified as search tasks, and the rest are classified as prediction tasks.

Supplementary Note S3. Question generation prompts for generation tasks

I have a model that generates the structures with specific properties, and you need to generate a question to match. The question should be as diverse as possible, and should be something a human would ask the model. The property should be one of [bandgap, hydrogen uptake (100bar), hydrogen diffusivity, O2 diffusivity, N2 diffusivity, O2 uptake, N2 uptake, thermal stability, CO2 henry coefficient, surface area, largest cavity diameter, pore limiting diameter, pore volume, volume fraction] You add temperature 298K and 1 bar condition for each question with property listed in ['diffusivity', 'uptake', and 'henry coefficient'].<br><br>Example:<br>- Can you generate the structures with highest PLD?<br>- Can you generate the structures with band-gap near 0.4?<br>- Can you generate the structures with smallest bandgap?<br><br>Generate 30 question.<br><br>Begin!

For 298K and 1 bar, select the appropriate temperature and pressure for the property.

Supplementary Note S4. Prompt for planning in the searcher

```
You are working with a pandas dataframe in Python. The name of the dataframe is `df`.
You should make a valid python command as input. You must use print the output using the `print` function at the end.
You should use the `to_markdown` function when you print a pandas object.

Use the following format:

Question: the input question you must answer
Thought: you should always think about what to do
Input: the valid python code only using the Pandas library
Observation: the result of python code
... (this Thought/Input/Observation can repeat N times)
Final Thought: you should think about how to answer the question based on your observation
Final Answer: the final answer to the original input question. If you can't answer the question, say `nothing`

The index of the dataframe must be be one of {df_index}. If it's not in the index you want, skip straight to Final Thought.
{information}

Begin!

Question: What is the head of df? If you extracted successfully, derive 'success' as the final answer
Thought: To get the head of a DataFrame, we can use the pandas function head(), which will return the first N rows. By default, it returns the first 5 rows.
Input:
```
import pandas as pd
import json
print(df.head().to_markdown())
```
Observation: {df_head}
Final Thought: The head() function in pandas provides the first 5 rows of the DataFrame.
```

```
   Final Answer: success



   Question: {question}
{agent_scratchpad}
```

Supplementary Note S5. Prompt for planning in the predictor

```
plan to use machine learning to predict the properties of matter. To answer the question, you have to fill in the following format:

Question: the input question you must answer
Thought: you should always think about what to do
Property: the property you can predict, should be one of [{model_names}]
Material: names of materials separated using comma. If you need to proceed for all material, write *. To proceed for a specific topology, append the topology name with an * (ex. pcu*)
... (this Property/Material can repeat N times)
Run Machine Learning: nothing to do
Final Thought: you should think about how you will derive a final answer from the results of machine learning.

Begin!

Question: predict the surface area and hydrogen uptake of ACOPEN and ELOBPE.
Thought: I need to gather data on ACOPEN and train a machine learning model to predict its surface area and hydrogen uptake.
Property: surface_area
Material: ACOPEN, ELOBPE
Property: hydrogen_uptake_100bar_77K
Material: ACOPEN, ELOBPE
Final Thought: Based on the result, answer the question using predicted surface area and the predicted hydrogen uptake at 100 bar.

Question: which MOF has a highest band-gap?
Thought: I need to gather data on the band-gap of different structures and compare them to find the one with the highest value.
Property: bandgap
Material: *
Final Thought: Based on the result, find the structure with the highest predicted band-gap value.

Question: Predict surface area and save results in csv format, only pcu topology.
Thought: I need to gather data on the surface area of materials with pcu topology and train a machine learning model to predict their surface area. Then, I can use the model to make predictions and save the results in a csv format.
Property: surface_area
Material: pcu*
Final Thought: The predicted surface area values for materials with pcu topology can be found in the saved csv file.

Question: {question}
```

Supplementary Note S6. Prompt for single data retrieval in predictor

```
You need to answer the question from the markdown table below


Markdown Table:
{table}


{information}


Question: {question}
Answer:
```

Supplementary Note S7. Prompt for planning in generator

Create a plan to generate material based on the following question.<br><br>Use the following format:<br><br><br>Question: the input question you must to answer<br><br>Thought: you should always think about what to do<br><br>Property: the property you can predict, must be one of [{model_names}]<br><br>Objective: you should decide what criteria you wan to generate by.<br><br>Search look-up table: plan to extract 100 material for the purpose from the look-up table where the property is pre-calculated.<br><br>Genetic algorithm: plan to create a new materials using the 100 extracted materials.<br><br>Final thought: get a final answer based on the structures you generate.<br><br><br>Begin!<br><br>Question: generate a material with a porosity of 0.5 and surface area of 120 m2/g<br><br>Thought: I need to generate a material with a porosity value of 0.5 and surface area of 120 m2/g.<br><br>Property: void_fraction, accessible_surface_area<br><br>Objective: near 0.5, near 120<br><br>Search look-up table: extract cif_id, void_fraction, and accessible_surface_area of 100 materials with porosity close to 0.5 and surface area near 120 m2/g from look-up tables.<br><br>Genetic algorithm: create a new material with a porosity close to 0.5 and surface area near 120 m2/g from 100 materials<br><br>Final Thought: Based on the generated CIF, find the material that is closest to a porosity of 0.5 and a surface area of 120 m2/g.<br><br><br>Question: generate a material with a highest band-gap<br><br>Thought: I need to generate a material with a highest band-gap.<br><br>Property: bandgap

Objective: max

Search look-up table: extract cif_id and bandgap of 100 materials with high band-gap value from look-up tables.

Genetic algorithm: generate 100 new materials with the highest band gap from the 100 materials.

Final Thought: Based on the generated CIF, find the material that has highest band-gap.

Question: {question}

Supplementary Note S8. Prompt for genetic algorithm in generator

"You should act as a generator to find the optimal material. A substance consists of a block1, block2, and must maintain the order. I will give you 100 parent materials. Based on these, you must answer as many new children as you expect to answer the question. The block1 and block2 used in the child must be the blocks used in the parent, and you must not create blocks that don't exist. You must generate children diverse. "The children must not be duplicates of existing parents or already created children. You output children only and nothing else.<br><br>Begin.<br>Question: {question}<br>Parent:<br>V12+T31, V24+T32, V7+T12<br>4 new Children:<br>V12+T12, V24+T31, V7+T31, V7+T32<br>Parent:<br>{parents}<br>100 new Children:

The objective of the genetic algorithm prompt is to stimulate the creation of materials that are not simply sequencing permutations of their parents, but instead generate diversity so that the child can explore a wide range of material space. This approach aims to ensure that the emerging materials encompass a broad array of potential structures, which is essential for pinpointing prospective contenders in material design. We endeavored to minimize repetition and enhance the diversity of the children MOFs. Additionally, it was set to yield 100 children to balance the generation of ample datasets against the constraints of the token limit.

Supplementary Note S9. Prompt for planning in agent

```
You act like a material scientist answering a question. Answer the following questions as best you can. You have access to the following tools:

{tools}


You must not use tool `generator` when `Question` does not required to generate.


Use the following format:


Question: the input question you must answer
Thought: you should always think about what to do
Action: the action to take, should be one of [{tool_names}]
Action Input: the input to the action
Observation: the result of the action
... (this Thought/Action/Action Input/Observation can repeat N times)
Thought: I now know the final answer
Final Answer: the final answer to the original input question


Begin!


Question: What is the surface area and bandgap of ACOGEF?
Thought: I need to find the surface area of ACOGEF.
Action: search_csv
Action Input: "Search name ACOGEF and provide information on its surface area"
Observation: The surface area of material "ACOGEF" is 1138.35 m^2/g. Check to see if this answer can be you final answer, and if so, you should submit your final answer.
Thought: The search_csv tool provided the surface area of ACOGEF, but not the bandgap. I need to find the bandgap.
```

Action: search_csv

Action Input: "Search name ACOGEF and provide information on its bandgap"

Observation: The search_csv tool did not provide any information on the bandgap of ACOGEF. I need to find another way to obtain this information.

Thought: The search_csv tool provided the surface area of ACOGEF, but not the bandgap. I need to find the bandgap.

Action: predictor

Action Input: "Predict the bandgap of ACOGEF"

Observation: The bandgap of material "ACOGEF" is 3.41139 eV. Check to see if this answer can be you final answer, and if so, you should submit your final answer.

Thought: I now know the final answer

Final Answer: The bandgap and surface area of ACOGEF is 3.41149 eV and 1138.35 m^2/g.

Question: What is the highest bandgap MOF?

Thought: I need to find the MOF with the highest bandgap.

Action: search_csv

Action Input: "find for MOFs with highest bandgap and provide its name and bandgap"

Observation: There are no data in database

Thought: The search_csv tool did not provide any information on the bandgaps of MOFs. I need to find another way to obtain this information.

Action: predictor

Action Input: "predict the bandgaps of MOFs and find the name of MOF with highest bandgaps"

Observation: The highest bandgap MOF is ACOGEF.

Thought: I now know the final answer

Final Answer: The highest bandgap MOF is ACOGEF.

Question: {input}

Thought:{agent_scratchpad}

Supplementary Note S10. Prompt for planning in unit-converter

You should have a plan for converting units for metal-organic frameworks. The question specifies the unit to be converted from and the desired target unit for the property.

You need to explain the conversion process and identify any additional information required. To answer the question, you have to fill in the following format:

Question: the input question you must answer

Thought: you should always think about what to do

Unit: unit to be converted from and the desired target unit

Equation: you should explain the conversion process

Information: you should answer the information you need in this process

You must be careful with units. The units must match the final units when they are computed.

Begin!

Question: Change the m^2/g unit for surface area to m^2/cm^3

Thought: Thought: To convert the surface area from m^2/g to m^2/cm^3, we need to convert the mass unit from grams to cubic centimeters. This requires the knowledge of the density of the metal-organic framework.

Unit: The unit to be converted from is m^2/g and the desired target unit is m^2/cm^3.

Equation: Surface area (m^2/cm^3) = Surface area (m^2/g) * Density (g/cm^3)

Information: The additional information required for this conversion is the density of the metal-organic framework in g/cm^3.

Question: Change the mol/kg unit for H2 uptake to cm^3/cm^3

Thought: To convert the H2 uptake from mol/kg to cm^3/cm^3, we need to convert the amount of substance from moles to volume and the mass from kilograms to cubic centimeters. This requires the knowledge of the molar volume of H2 at STP and the density of the metal-organic framework.

Unit: The unit to be converted from is mol/kg and the desired target unit is cm^3/cm^3.

Equation: H2 uptake (cm^3/cm^3) = H2 uptake (mol/kg) * Molar volume of H2 (cm^3/mol) * Density (kg/cm^3)

Information: The additional information required for this conversion is the molar volume of H2 at STP (which is 22.4 L/mol or 22400 cm^3/mol at standard temperature and pressure) and the density of the metal-organic framework in kg/cm^3.

Question: {question}

Supplementary Note S11. Prompt for planning in ASE repl tool

You are working with a ase library in Python. The name of the object `ase.atoms` is `atoms`.

You should make a valid python command as input. You must use print the output using the `print` function at the end.

Use the following format:

Question: the input question you must answer

Material: name of material

Convert: convert material to atoms object.

Thought: you should always think about what to do

Input: the valid python code only using the ase library

Observation: the result of python code

... (this Thought/Input/Observation can repeat N times)

Final Thought: you should think about how to answer the question based on your observation

Final Answer: the final answer to the original input question. If you can't answer the question, say `nothing`

Begin!

Question: calculate the cell volume of ABAYOU

Material: ABAYOU

Convert: Convert material to atoms object. I now have to write a code using `atoms` object

Thought: To calculate the cell volume of `atoms`, I need to access the cell information of the atoms object and then use the appropriate method to calculate its volume.

Input:

```
```

```
import ase

volume = atoms.get_volume()

print(volume)
```

Observation: 18921.03834202299

Final Thought: The volume of the cell associated with the ase.atoms object atoms was successfully calculated using the get_volume() method from the ASE library.

Final Answer: The cell volume of the ABAYOU is 18921.03834202299 cubic Ångströms.

Question: {question}

{agent_scratchpad}

Supplementary Note S12. Analyzing ChatMOF with LLaMA2

LLaMA2 (Language Model for Many Applications) is an open-source, large-scale, high-performance language model developed by Meta AI.[1,2] Designed for research and commercial use, it offers the advantage of direct access to a large language model (LLM). We evaluated the accuracy of two versions of ChatMOF with different LLMs, "Llama-2-7B-chat-hf" and "Llama-2-13B-chat-hf". Their accuracy results are detailed in Supplementary Table 4. Unfortunately, both models underperform in the ChatMOF environment. We categorized accuracy metrics into specific areas: True, errors in the agent (Agent), challenges in evaluating and interpreting outcomes (Evaluation), issues in the planning stage for problem resolution (Plan), difficulties in generating code for problem-solving (Code), and issues related to the LLM's tokens (Token). In scenarios with multiple concurrent issues, we prioritized the initial error for measurement. Regrettably, both models exhibited suboptimal performance in the ChatMOF framework. Further examination revealed that these models struggled not only with planning but also significantly in code generation. Notably, a substantial portion of these errors (45%, 67% in search tasks and 21%, 37% in prediction tasks, 7B and 13B model respectively) were linked to code generation, leading to a marked decline in accuracy when compared to the GPT models. This decrease is attributed to LLaMA2's inferior code-generating capabilities compared to GPT-3.5 and GPT-4.[3] Moreover, GPT-4 surpasses LLaMA2 in both few-shot and zero-shot training scenarios.[2] These findings indicate that LLaMA2 might not be an ideal choice for deployment in the ChatMOF system.

Supplementary Note S13. Collaborative Online Platform

One limiting factor of ChatMOF is the performance reliance on the number of fine-tuned models in the MOFTransformer used in the predictor task. An increased quantity of fine-tuned models allows for the prediction of more properties, thereby enabling more active prediction and generation processes. However, each user faces constraints on the number of models that can be utilized, given that it is unrealistic for one individual to possess all the data.

To train a model, the collection of experimental data or the execution of computational simulations is necessary. While some calculations, such as pore limiting diameter or surface area, demand less time, other tasks such as band-gap, HOMO, and LUMO calculations are considerably more computationally demanding. The generation and training of data for these complex tasks can be quite cumbersome.

To address this challenge, an online platform was developed where users can freely upload their customized fine-tuned models. The ChatMOF-online platform operates on the principle that HuggingGPT[4] selects the most appropriate model from those available on HuggingFace platform. Supplementary Figures S11 and S12 depict the scheme of the ChatMOF-online platform and how it can be used to predict properties that are not within the capabilities of the user's fine-tuned models. Users can upload their own fine-tuned models to ChatMOF-online, making these models readily available and downloadable by others. When ChatMOF needs to make a prediction and the required model is not in the user's collection, it searches for it on the ChatMOF-online platform. In case a suitable fine-tuned model is found on the platform, it is downloaded and used for prediction. This functionality significantly expands the range of properties ChatMOF can predict, enhancing its utility as a robust tool for predicting MOF properties.

Supplementary Note S14. Fine-tuning agent

Fine-tuning refers to the further adjustment of the parameters of a pre-trained model to fit a specific task or data, and research is being conducted on fine-tuning LLM to help users perform the desired task.[5-7] Although fine-tuning offers the advantage of efficiently repeating a designated task, it may reduce answer diversity and model generality due to its reliance on the fine-tuning dataset.[8]

In the context of our ChatMOF application, we evaluated the fine-tuned LLM, with a focus on the Agent and Table search tool. The evaluation used 90% of the data for training and 10% for testing, based on correct answers derived from questions tested with GPT-4. The agent underwent training with 83 search tasks and 83 prediction tasks (432 question-answer pairs), while 83 search tasks (182 question-answer pairs) were used as data for the table agent. According to OpenAI API, the gpt-3.5-turbo-0613 model underwent three epochs of training and achieved training losses of <0.0001 and 0.0949 for the agent and table search tool, respectively.

The fine-tuned model's performance was then evaluated using ChatMOF on a test set consisting of 10 search tasks. Notably, our model successfully completed all 10 tasks and outperformed GPT-4 in 1 out of 7 tasks where GPT-4 encountered difficulties (specifically, search task 65). This observation presents convincing evidence that the fine-tuned model can produce dependable answers based on the training data it was exposed to.

The response generated by the fine-tuning model is significantly influenced by the original training data and can show independence from the system prompt. Supplementary Figure S13 displays ChatMOF's response, fine-tuned with GPT, to the question 'What is the non-accessible surface area of ARAHIM02?'. In the initial version 0.0.0 of ChatMOF (version for accuracy test), the training dataset included the accessible surface area in the table, denoting both m^2/g and

m^2/cm^3 units. However, in the recent ChatMOF 0.1.0 version, the unit m^2/cm^3 has been replaced with m^2/g. Therefore, even though the m^2/cm^3 property is not included in the system prompt, the model still generates data in this unit, resulting in a KeyError in the latest version. This incident highlights the model's increased dependence on existing training data compared to the system prompt.

This scenario demonstrates that fine-tuned models perform well in accurately answering questions that are similar to the ones in the training data, but are less effective for inquiries that are not included in the prior dataset. Supplementary Figure S14 shows the fine-tuning agent's response to the same question as in Supplementary Figure S3. Unlike the original agent, which immediately uses the ASE_repl tool, the fine-tuning agent first initiates the search_csv and predictor tools. The fine-tuning agent's preference for existing training data over the system prompt is highlighted by its similarity to the earlier case. Furthermore, Supplementary Figure S15 demonstrates the fine-tuning agent's responses to questions identical to those in Figure 6. The standard GPT-4 agent uses the predictor and then applies the convert_unit tool for unit conversion. On the other hand, the fine-tuning agent provides the final answer without any unit conversion, which lead to incorrect results.

Therefore, diversifying the types of training data is essential to optimize the application of fine-tuning in the ChatMOF system. ChatMOF can provide more reliable answers than a general LLM when human queries align with the trained data. However, efficiency diminishes and the risk of providing inaccurate responses increases for questions unrelated to the training data. The data and code used for training are available on GitHub.

Supplementary Note S15. Evaluation of ChatMOF at different temperatures of GPT.

To evaluate ChatMOF's performance in relation to temperature, we conducted a systematic evaluation. Adjusting the temperature in GPT allows for control over the randomness of generated text. Higher temperatures yield more varied and creative responses, but with less consistency and a higher probability of randomness. Conversely, lower temperatures result in more accurate and conservative answers, leading to a more consistent generation of text. The relationship between temperature and output characteristics is a crucial aspect of language model applications.

In our evaluation, ChatMOF was executed with temperatures ranging from 0.0 to 1.0 in 0.2 increments. We selected four questions of varying difficulty, determined by the number of tools executed by ChatMOF (N_{step}). ChatMOF's planning and execution were evaluated using GPT-4, which provided an Evaluation Score on a scale of 0 to 100. Five tests were conducted for each temperature setting. The prompt for evaluator and questions were provided in supplementary Note S16.

Supplementary Figure S16(a) shows the average Evaluation Score for GPT-4 at each temperature. For the easiest task (N_{step}=1), the score consistently remains high across temperature variations. However, when faced with a difficult task, the average evaluation score tends to decrease as temperatures rise. Supplementary Figure S16(b) displays the standard deviation of five evaluation scores, demonstrating that as the temperature increases, the diversity of generated sentences increases. As ChatMOF aims to provide accurate information to users, it is recommended to operate at lower temperatures for optimal performance.

Supplementary Figure S16(c) and (d) show the mean and standard deviation of the evaluation for GPT-3.5-turbo. GPT-3.5-turbo exhibits lower mean values and higher standard deviations

compared to GPT-4, particularly for $N_{step}>1$. This indicates that GPT-3.5-turbo produces less stable responses than GPT-4, highlighting the significant impact of language model performance on ChatMOF outcomes. These findings reveal the complex relationship between temperature, model architecture, and the effectiveness of language models in ChatMOF applications.

Supplementary Note S16. Prompt for Evaluation Score

```
You are the evaluator of the chatbot.

You have to rate the process of chatbot on a scale of 0-100 on how well it planned to solve the question and how well it executed.


Question : {question}


True Answer: {answer}


Key Point : {keypoint}


Chatbot Process : {process}


Score :
```

Questions:

"What is the largest cavity diameter of MIBQAR16?", (1 step)
"How does the density of LITDAV compare to other materials", (2 step)
"What is the relationship between H2 uptake and accessible volume in MOFs?", (4 step)
"Provide the CO2 henry coefficient of XEGKUR at 298 K in mol/cm3Pa", (8 step)

Answers:

'The largest cavity diameter of MIBQAR16 is 15.0001 Å', (1 step)
'The density of LITDAV is 1.01002 g/cm^3, which is lower than the average density of other materials, which is 1.373 g/cm^3.', (2 step)
'The correlation between H2 uptake and accessible volume in MOFs is approximately 0.6, indicating a moderate positive relationship.', (4 step)
"The CO2 Henry's coefficient of XEGKUR at 298 K is 2.75e-05 mol/cm3Pa.", (8 step)

Keypoints:

"How efficiently can you write code to get a property?", (1 step)
"How efficiently can you write code to get a property? How efficiently can you perform a 'compare to other material'?", (2 step)
"How efficiently can you write code to get a property? How do I get the relationship?", (4 step)
"How efficiently can you write code to get a property? Does it do the unit conversion correctly? Does it do a good job of finding the density needed for the unit conversion?", (8 step)

Supplementary Note S17. Diversity of questions

This study evaluates the accuracy of ChatMOF using questions generated by GPT-4. The diversity of these queries significantly impacts the evaluation of model performance. To assess this diversity, we conducted a comparative analysis between the questions generated by GPT-4 (original), GPT-3.5-turbo under identical prompts with GPT-4, and a template-based method. The template-based method involves a human generating questions by employing 10 predefined templates, with random variations in properties, materials, and values.

The Type-Token Ratio (TTR) was used to assess the lexical diversity of each question set. TTR is an indicator of textual diversity based on the ratio of unique words to the total number of words. Supplementary Figure S17 presents the TTR values for each group. Both GPT-4 and template-based questions had similar type-token ratio (TTR) values of approximately 0.11. GPT-3.5-generated sentences, however, had lower TTR values.

Furthermore, the text-embedding-3-small model was used to convert each sentence into a 1536-dimensional embedding size vector in order to determine diversity, using GPT. The diversity score, which represents the average pairwise distance between these vectors, was then calculated. These results are visually represented in Figure S17(b). Upon analysis of the diversity score, it was found that the GPT-4 model achieved the highest score. Figure S17(c) shows the two-dimensional embedding vectors obtained through principal component analysis (PCA). The figure indicates that the distribution of the GPT-4 model is relatively widespread compared to other models.

Having an expert from MOF devise 100 different questions would be the ideal way to maximize distinctiveness. However, given time and resource efficiency, GPT-4 would be a good

resource for question generation. The code and questions used in the diversity measurement process can be accessed in the experimental section on GitHub.

The TTR follows the folumla:

$$TTR = \frac{Number\ of\ Unique\ Words\ (Types)}{Total\ number\ of\ Words\ (Tokens)}$$

The Diversity score $\overline{D}(\mathrm{E})$ follows the formula:

$$\overline{D}(\mathrm{E}) = \frac{2}{n(n-1)}\sum_{i=1}^{n-1}\sum_{j=i+1}^{n}\mathrm{D}(e_i, e_j)$$

Where

$$\mathrm{D}(e_i, e_j) = \sqrt{\sum_{k=1}^{d}(e_{ik} - e_{jk})^2}$$

You

What is the surface area of JUKPAI?

ChatGPT

To determine the surface area of JUKPAI, I need more specific information. "JUKPAI" doesn't appear to correspond to a commonly recognized term or object. Could you please clarify what JUKPAI is? If it's a geometric shape, an object, or something else entirely, providing additional details will enable me to assist you more effectively.

You

Can you generate the MOF with largest surface area?

ChatGPT

As an AI developed by OpenAI, I'm unable to physically synthesize materials like Metal-Organic Frameworks (MOFs), which are what you seem to be referring to. MOFs are complex crystalline compounds made up of metal ions or clusters coordinated to organic ligands to form one-, two-, or three-dimensional structures.

However, I can provide some information about MOFs with large surface areas. As of my last training data, MOFs like NU-110, MOF-177, and HKUST-1 were known for having exceptionally high surface areas. The record for the highest surface area among MOFs has been continually evolving with new discoveries in the field.

To generate a MOF with the largest surface area, researchers typically focus on creating a highly porous structure, often by carefully selecting metal nodes and organic linkers that allow for a highly regular, extensive pore structure. This requires sophisticated techniques in chemistry and materials science and is usually done in specialized laboratory settings.

If you're looking for detailed instructions or specific designs for synthesizing such a MOF, you would typically need access to scientific literature and research in the field of materials science.

Figure S1. Responses to MOF-related questions posed directly to GPT-4. The answer was created on 23/11/11

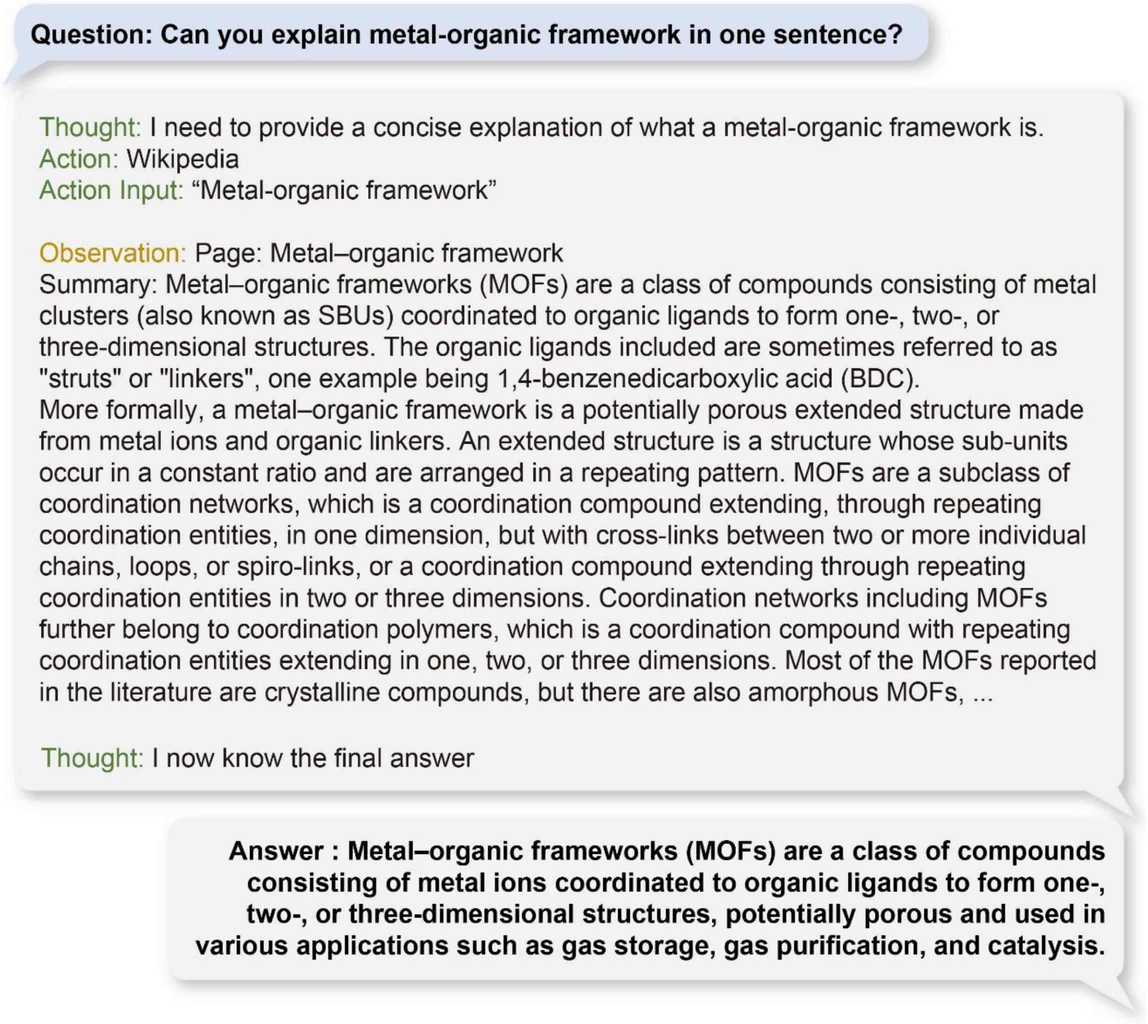

Figure S2. Example of an internet-search for the question “Can you explain metal-organic framework in one sentence?”. “Tested on November 17, 2023 at 21:49 KST with GPT-4.”

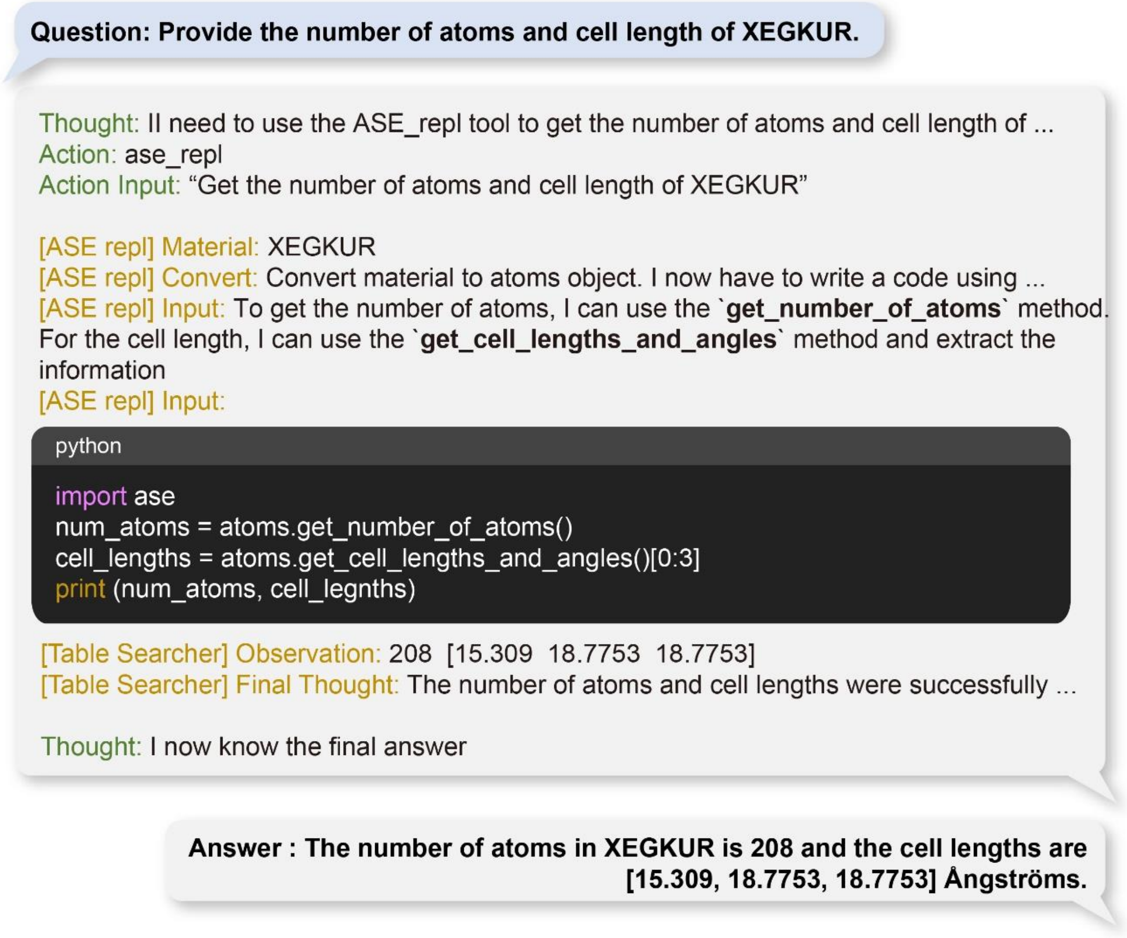

Figure S3. Example of an ASE repl tool for the question “Provide the number of atoms and cell length of XEGKUR”. “Tested on November 17, 2023 at 21:48 KST with GPT-4.”

Question: What is the relationship between H_2 uptake and accessible volume in MOFs?

Thought: I need to find the relationship between H2 uptake and accessible volume in MOFs.
Action: search_csv
...
Thought: The search_csv tool did not provide any information on the relationship between H2 uptake and accessible volume in MOFs. I need to find another way to obtain this information.
Action: predictor
Action Input: Predict the relationship between H2 uptake and accessible volume in MOFs

[predictor] Thought: I need to gather data on H2 uptake and accessible volume in MOFs and train a machine learning model to predict the relationship between these two properties.
[predictor] Property: hydrogen_uptake_100bar_77K
[predictor] Materials: *

bash
```
load model : hydrogen_uptake_100bar_77K/best.ckpt
Predicting DataLoader 0: 100% | ■■■■■■■■■■■■| 1246/1246  [00:45<00:00]
```

[predictor] Property: accessible_volume
[predictor] Materials: *

bash
```
load model : accessible_volume/best.ckpt
Predicting DataLoader 0: 100% | ■■■■■■■■■■■■| 1246/1246  [00:45<00:00]
```

[predictor] Final Thought: Based on the result, analyze the relationship between H2 uptake and accessible volume in MOFs.

[Table Searcher] Thought: To analyze the relationship between H2 uptake and accessible volume in MOFs, we can use the correlation function in pandas which computes pairwise correlation of columns, excluding NA/null values.
[Table Searcher] Input:

python
```python
print(df[['hydrogen_uptake_100bar_77K', 'accessible_volume']].corr().to_markdown())
```

[Table Searcher] Observation:

	hydrogen_uptake_100bar_77K	accessible_volume
hydrogen_uptake_100bar_77K	1	0.59717
accessible_volume	0.59717	1

[Table Searcher] Observation: There is a moderate positive correlation between hydrogen uptake and accessible volume in MOFs. As the accessible volume increases, the hydrogen uptake also tends to increase.

Thought: I now know the final answer

Answer : There is a moderate positive correlation between hydrogen uptake and accessible volume in MOFs. As the accessible volume increases, the hydrogen uptake also tends to increase.

Figure S4. Example of a complex and multi-step question "What is the relationship between H_2 uptake and accessible volume in MOFs?". "Tested on November 17, 2023 at 21:45 KST with GPT-4."

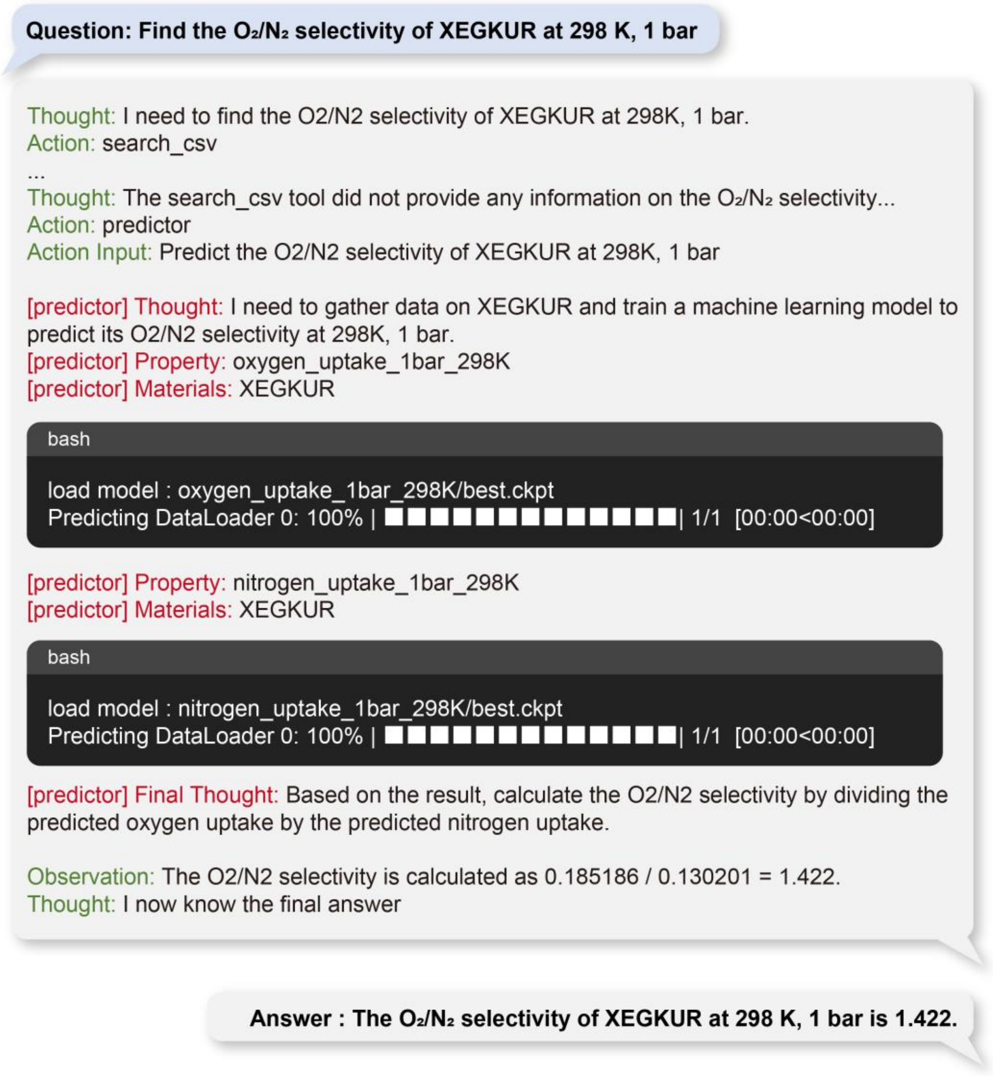

Figure S5. Example of a complex and multi-step question "Find the O_2/N_2 selectivity of XEGKUR at 298K, 1bar". Tested on November 17, 2023 at 10:26 KST with GPT-4."

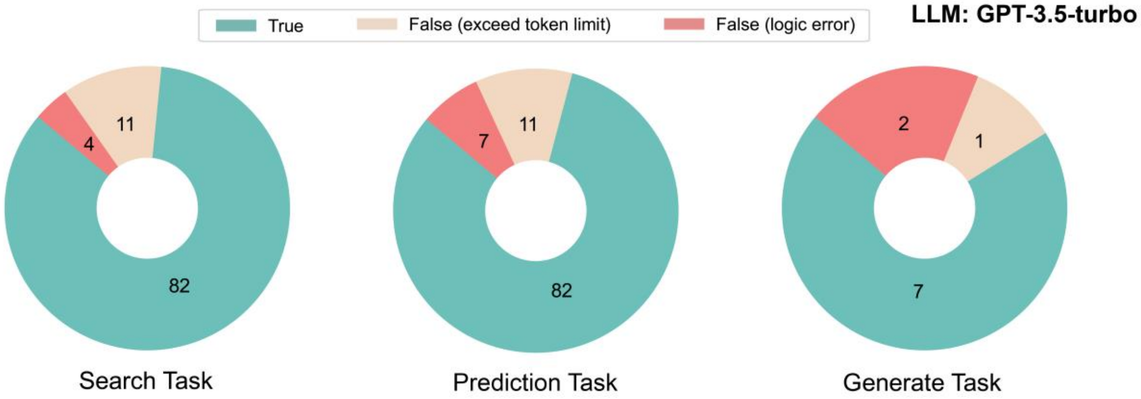

Figure S6. Depiction of accuracies for three tasks using GPT-3.5-turbo model - search, prediction, and generation (tested on 23.07.18 to 23.07.21). Accuracies were evaluated based on three labels: True, False (token limit exceeded), and False (logic error). False (Exceed Token Limit) indicates that the LLM has exceeded the maximum token allowance, while False (Logic Error) indicates that the ChatMOF's logic has resulted in an incorrect response or anomaly. The number in the bar represent the count of each label.

gpt-4

```
########## Question ##############################
How does the pore limiting diameter of YUSGID_clean compare with other materials?


> Entering new AgentExecutor chain...
I need to find the pore limiting diameter of YUSGID_clean and compare it with other
materials.
Action: search_csv
Action Input: "Search name YUSGID_clean and provide information on its pore limiting
diameter"

> Entering new TableSearcher chain...

[Table Searcher] Thought: To find the information for a specific name in the DataFrame,
we can use the loc function in pandas. This function allows us to access a group of rows
and columns by label(s) or a boolean array.
[Table Searcher] Input: print(df.loc[df['name'] == 'YUSGID']['Pore limiting diameter
(Å)'].to_markdown())
[Table Searcher] Observation:
|       |   Pore limiting diameter (Å) |
|------:|-----------------------------:|
| 11739 |                      3.71515 |
[Table Searcher] Final Thought: The 'Pore limiting diameter (Å)' for the material with the
name 'YUSGID' is 3.71515 Å.
> Finished chain.

Observation: The 'Pore limiting diameter (Å)' for 'YUSGID' is 3.71515 Å.
Thought:I have the pore limiting diameter for YUSGID_clean. Now I need to compare
this with the pore limiting diameters of other materials.
Action: search_csv
Action Input: "Provide information on the pore limiting diameters of all materials"

> Entering new TableSearcher chain...

[Table Searcher] Thought: To get information on the pore limiting diameters of all
materials, we can use the pandas function describe(), which provides descriptive
statistics of the DataFrame or Series.
[Table Searcher] Input: print(df['Pore limiting diameter (Å)'].describe().to_markdown())
[Table Searcher] Observation:
|       |   Pore limiting diameter (Å) |
|:------|-----------------------------:|
| count |                  12020       |
| mean  |                      4.87759 |
| std   |                      2.78185 |
| min   |                      2.40008 |
| 25%   |                      3.24153 |
| 50%   |                      4.10336 |
| 75%   |                      5.64609 |
| max   |                     71.502   |
[Table Searcher] Final Thought: The describe() function in pandas provides a statistical
summary of the 'Pore limiting diameter (Å)' column in the DataFrame. This includes the
count, mean, standard deviation, minimum, 25th percentile, median (50th percentile),
75th percentile, and maximum.
> Finished chain.

Observation: The pore limiting diameters of all materials range from approximately 2.40Å
to 71.50Å, with an average diameter of approximately 4.88Å.
Thought:I now know the final answer
Final Answer: The pore limiting diameter of YUSGID_clean is 3.71515 Å, which is below
the average pore limiting diameter of approximately 4.88Å for all materials. The range of
pore limiting diameters for all materials is approximately 2.40Å to 71.50Å.

> Finished chain.


########## Output ##############################
The pore limiting diameter of YUSGID_clean is 3.71515 Å, which is below the average
pore limiting diameter of approximately 4.88Å for all materials. The range of pore limiting
diameters for all materials is approximately 2.40Å to 71.50Å.
```

gpt-3.5-turbo

```
########## Question ##############################
How does the pore limiting diameter of YUSGID_clean compare with other materials?


> Entering new AgentExecutor chain...
I need to compare the pore limiting diameter of YUSGID_clean with other materials.
Action: search_csv
Action Input: "Search name YUSGID_clean and provide information on its pore limiting
diameter"

> Entering new TableSearcher chain...

[Table Searcher] Thought: To search for a specific value in the "name" column, we can
use the pandas function loc[], which allows us to access a group of rows and columns by
label(s) or a boolean array. We can then extract the information on the pore limiting
diameter for the material with the name "YUSGID".
[Table Searcher] Input: print(df.loc[df['name'] == 'YUSGID', 'Pore limiting diameter
(Å)'].to_markdown())
[Table Searcher] Observation:
|       |   Pore limiting diameter (Å) |
|------:|-----------------------------:|
| 11739 |                      3.71515 |
[Table Searcher] Final Thought: The pore limiting diameter for the material with the
name "YUSGID" is 3.71515 Å.
> Finished chain.

Observation: The answer for question "Search name YUSGID and provide information
on its pore limiting diameter" is 3.71515 Å.
Thought:The search_csv tool provided the pore limiting diameter of YUSGID_clean. Now
I need to find the pore limiting diameter of other materials for comparison.
Action: search_csv
Action Input: "Search for other materials and provide information on their pore limiting
diameter"

> Entering new TableSearcher chain...

[Table Searcher] Thought: To search for other materials and provide information on their
pore limiting diameter, we can use the pandas function loc[] to filter the dataframe based
on a condition. We can then select the "name" and "Pore limiting diameter (Å)" columns
to display the information.
[Table Searcher] Input: print(df.loc[:, ["name", "Pore limiting diameter (Å)"]].to_mark-
down())
ValueError: The number of tokens has been exceeded.
```

Figure S7. ChatMOF's answer to the question "How does the pore limiting diameter of YUSGID_clean compare with other materials?" using GPT-4 and GPT-3.5-turbo. The answer is successfully derived for gpt-4 but raises a tokenError for GPT-3.5-turbo.

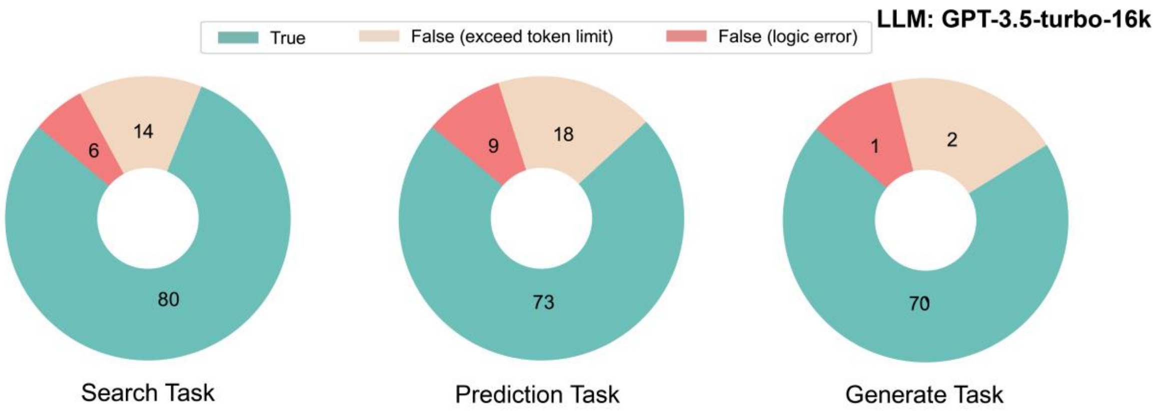

Figure S8. Depiction of accuracies for three tasks using GPT-3.5-turbo-16k model - search, prediction, and generation (tested on 23.11.09 to 23.11.11). Accuracies were evaluated based on three labels: True, False (exceeding token limit), and False (logical error). False (Exceed Token Limit) indicates that the LLM has exceeded the maximum token allowance, while False (Logic Error) indicates that the ChatMOF's logic has resulted in an incorrect response or anomaly. The number in the bar represent the count of each label

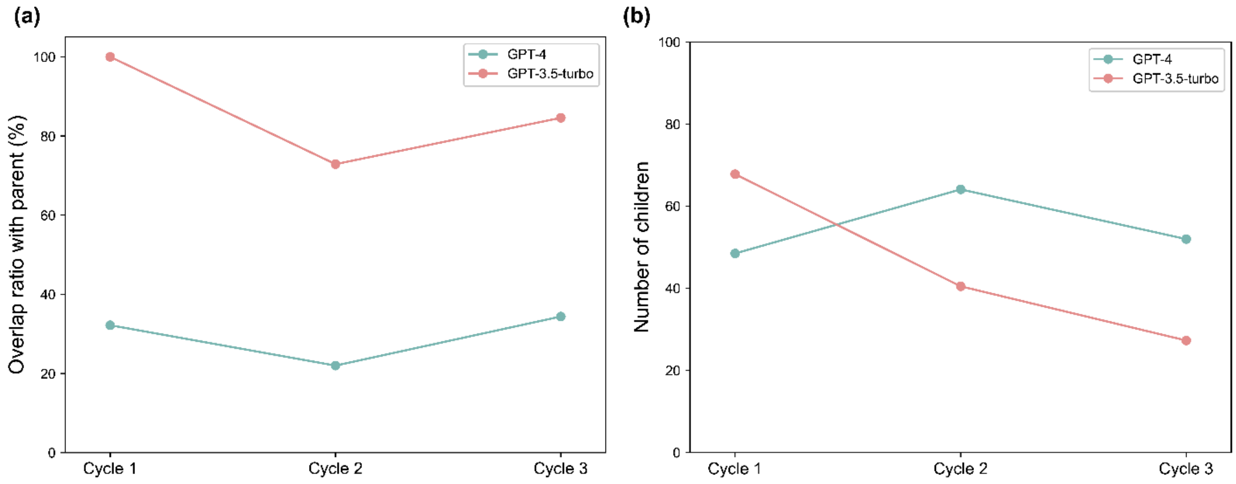

Figure S9. Evaluation of GPT-4 and GPT-3.5-turbo's genetic algorithm performance in generating structures with the largest accessible surface area. (a) Percentage of overlapping child genes with parent genes in each genetic algorithm cycle. (b) Average number of children generated per genetic algorithm cycle for GPT-4 and GPT-3.5-turbo when prompted to create 100 children. Tested on October 13, 2023, at 14:26

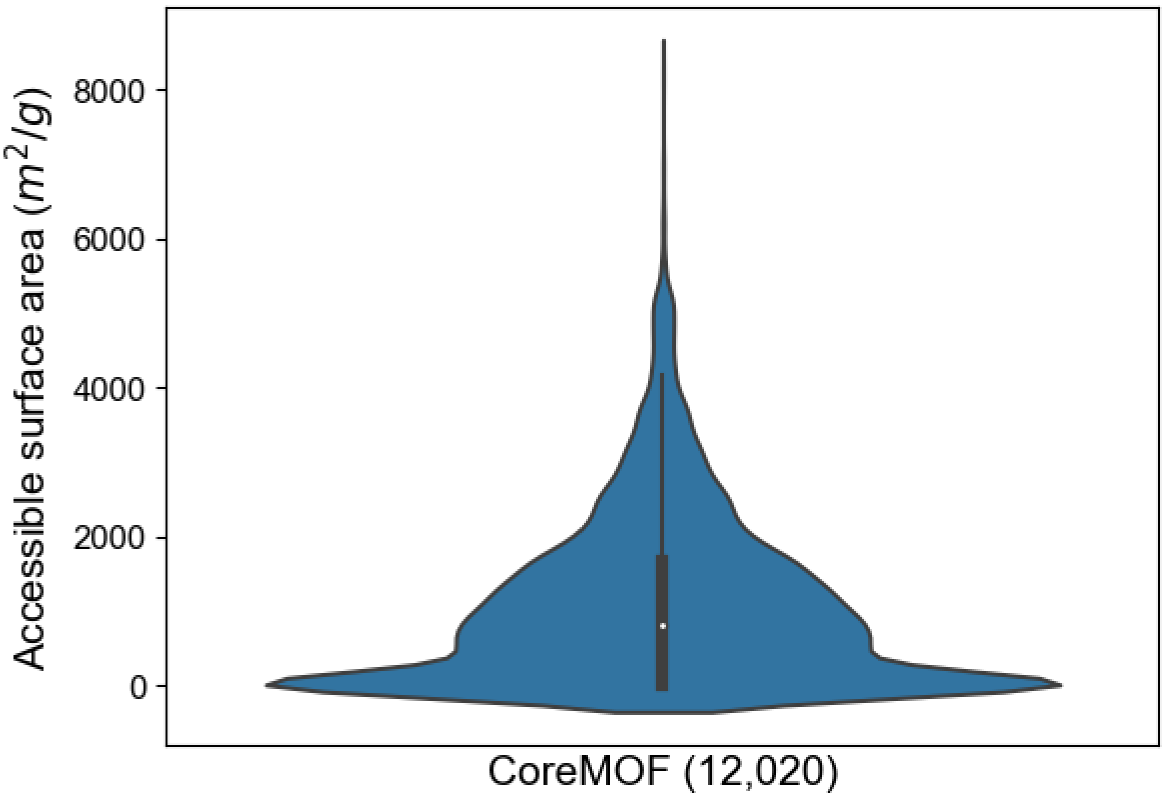

Figure S10. Violin plot illustrating the distribution of CoREMOF structure for a accessible surface area (m^2/g). The inner box represents the interquartile range (IQR) and the white horizontal line inside the box indicates the median of the distribution. The number in parentheses represents the number of data.

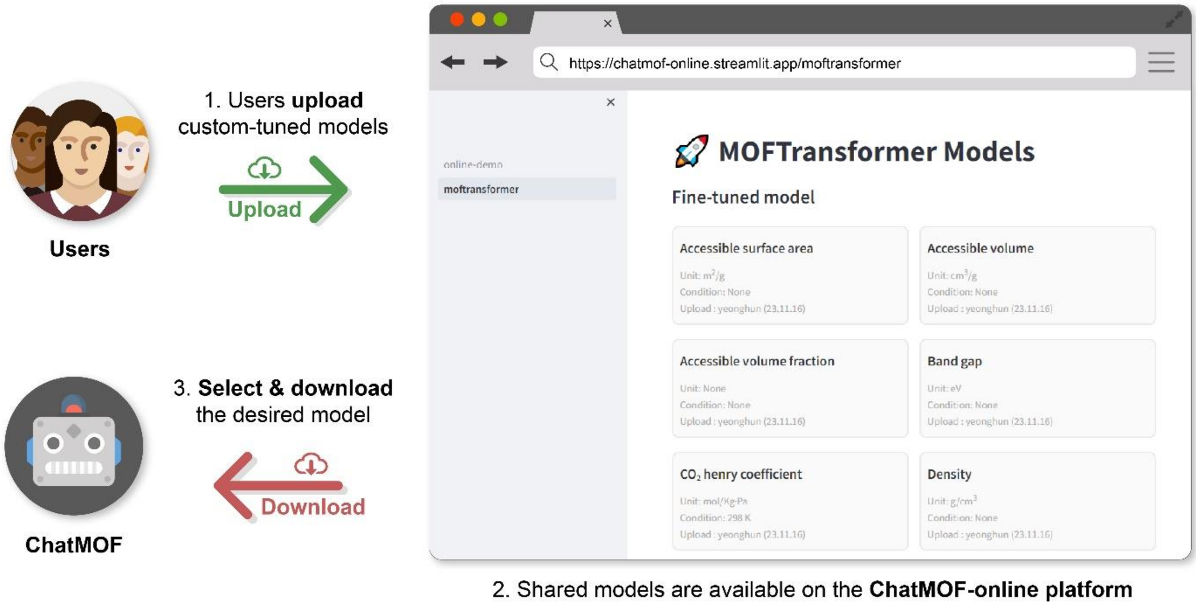

Figure S11. Illustration of ChatMOF-online platform. Customized fine-tuned models are uploaded and shared by users on this platform. In the process of running ChatMOF, the most suitable model is then selected from the ChatMOF-online platform, followed by its download and execution.

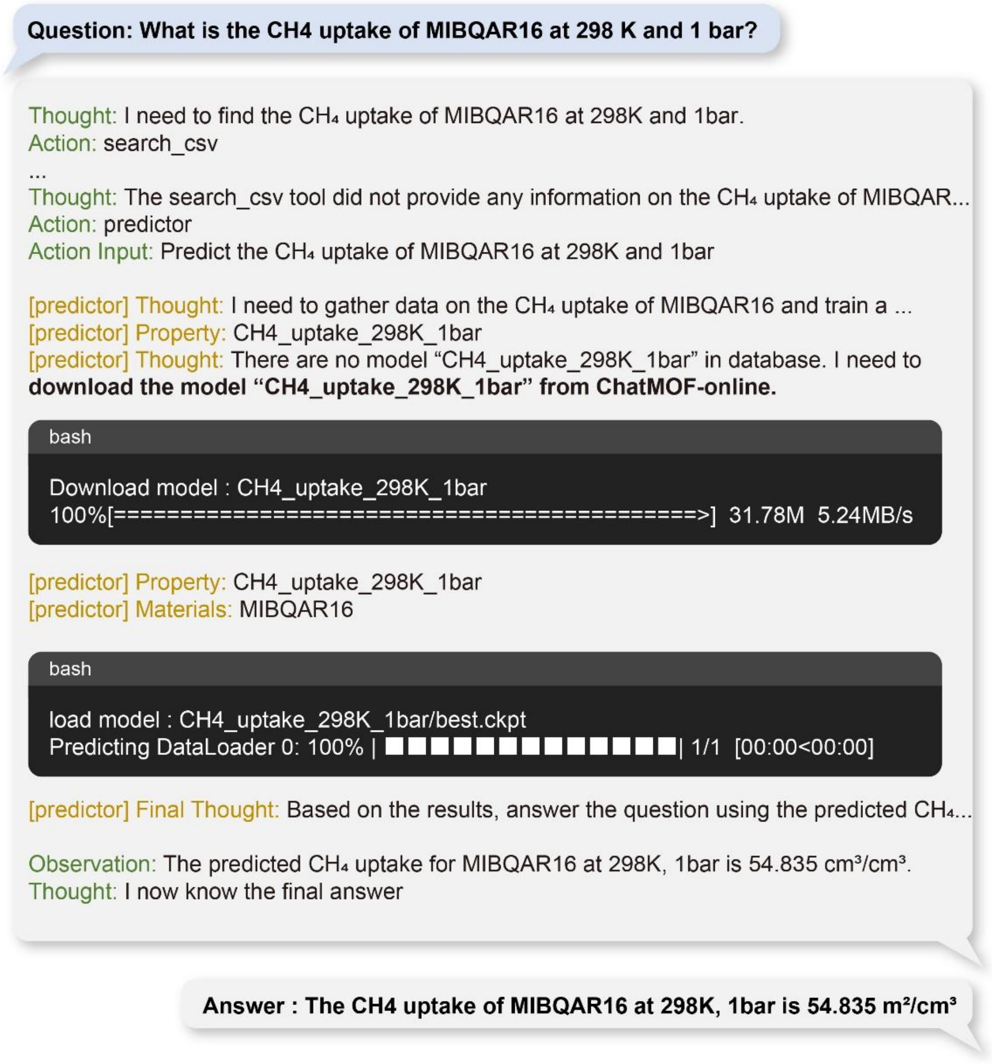

Figure S12. Example of ChatMOF's answer to the question "What is the CH_4 uptake of MIBQAR16 at 298 K and 1 bar". ChatMOF downloads the appropriate fine-tuned model from ChatMOF_online and proceeds to make property predictions.

Fine-tuning

Question: What is the non-accessible surface area of ARAHIM02?
Thought: I need to find the non-accessible surface area of ARAHIM02.
Action: search_csv
Action Input: "Search name ARAHIM02 and provide information on its non-accessible surface area"

Question: Search name ARAHIM02 and provide information on its non-accessible surface area
Thought: To find a specific row in a DataFrame based on a condition, we can use the loc function. In this case, we want to find the row where the 'name' column is equal to 'ARAHIM02' and then get the information on its non-accessible surface area.
Input: print(df.loc[df['name'] == 'ARAHIM02', ['Nonaccessible surface area (m^2/cm^3)', 'Nonaccessible surface area (m^2/g)']].to_markdown())
Observation: KeyError: "['Nonaccessible surface area (m^2/cm^3)'] not in index"
Thought: The 'Nonaccessible surface area (m^2/cm^3)' column does not exist in the DataFrame. We can only provide information on the 'Nonaccessible surface area (m^2/g)'.
Input: print(df.loc[df['name'] == 'ARAHIM02', 'Nonaccessible surface area (m^2/g)'].to_markdown())
Observation:
	Nonaccessible surface area (m^2/g)
327	23.5219
Final Thought: The non-accessible surface area of the material with the name 'ARAHIM02' is 23.5219 m^2/g.
Final Answer: The non-accessible surface area of 'ARAHIM02' is 23.5219 m^2/g.

Observation: The non-accessible surface area of 'ARAHIM02' is 23.5219 m^2/g.
Thought: I now know the final answer
Final Answer: The non-accessible surface area of ARAHIM02 is 23.5219 m^2/g.

Figure S13. ChatMOF's response with fine-tuned LLM to the question "What is the non-accessible surface area of ARAHIM02." The red text highlights the inefficient/wrong process generated by ChatMOF. The unit m^2/cm^3, which was not mentioned in the prompt, was generated during fine-tuning based on trained data. Tested on January 31, 2024, at 05:58 KST.

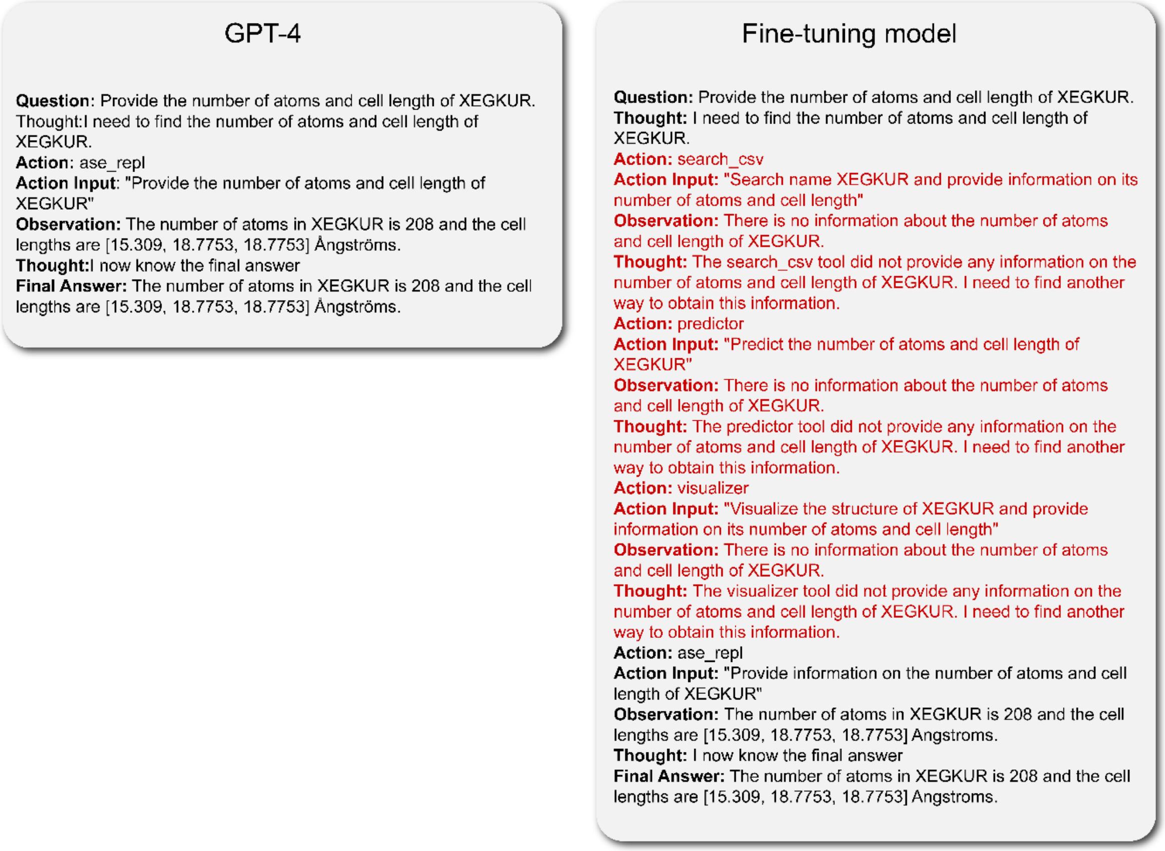

Figure S14. ChatMOF's response with fine-tuned Language Model (LLM) to the question "Provide the number of atoms and cell length of XEGKUR," compared with GPT-4. The red text highlights the inefficient/wrong process generated by ChatMOF, revealing challenges in tool selection for problem-solving during fine-tuning LLM. Tested on January 30, 2024, at 02:36 KST.

GPT-4

Question: Provide the CO2 henry coefficient of XEGKUR at 298 K in mol/cm3Pa
Thought: I need to find the CO2 Henry's coefficient of XEGKUR at 298K.
Action: search_csv
Action Input: "Search name XEGKUR and provide information on its CO2 Henry's coefficient at 298K"
Observation: There is no information about the CO2 Henry's coefficient at 298 K of XEGKUR.
Thought: The search_csv tool did not provide any information on the CO2 Henry's coefficient of XEGKUR at 298K. I need to find another way to obtain this information.
Action: predictor
Action Input: "Predict the CO2 Henry's coefficient of XEGKUR at 298K"
Observation: The predicted CO2 Henry's coefficient at 298K for XEGKUR is -3.62769 mol/Kg·Pa. However, this result is a logarithmic value. To get the original value, we need to apply an exponential.
Thought:I need to convert the logarithmic value to the original value.
Action: Python_REPL
Action Input: "import math\nprint(math.exp(-3.62769))"
Observation: 0.026577507595890823
Thought:I have the Henry's coefficient in mol/Kg·Pa, but I need to convert it to mol/cm^3Pa.
Action: unit_converter
Action Input: "Convert -3.62769 mol/Kg·Pa to mol/cm^3Pa"
Observation: Equation: H2 uptake (mol/cm^3·Pa) = H2 uptake (mol/kg·Pa) * Density (kg/cm^3), Information: The additional information required for this conversion is the density of the metal-organic framework in kg/cm^3.
Thought: I need to find the density of XEGKUR to complete the conversion.
Action: search_csv
Action Input: "Search name XEGKUR and provide information on its density"
Observation: The density of XEGKUR is 1.03463 g/cm^3.
Thought:I need to convert the density from g/cm^3 to kg/cm^3.
Action: unit_converter
Action Input: "Convert 1.03463 g/cm^3 to kg/cm^3"
Observation: Equation: Density (kg/cm^3) = Density (g/cm^3) / 1000, Information: No additional information is required for this conversion.
Thought: I can now calculate the density in kg/cm^3.
Action: Python_REPL
Action Input: "print(1.03463 / 1000)"
Observation: 0.00103463
Thought:I can now calculate the Henry's coefficient in mol/cm^3Pa.
Action: Python_REPL
Action Input: "print(0.026577507595890823 * 0.00103463)"
Observation: 2.749788668393652e-05
Thought: I now know the final answer
Final Answer: The CO2 Henry's coefficient of XEGKUR at 298K is 2.75e-05 mol/cm^3Pa.

Fine-tuning model

Question: Provide the CO2 henry coefficient of XEGKUR at 298 K in mol/cm3Pa
Thought: I need to find the CO2 henry coefficient of XEGKUR at 298 K in mol/cm3Pa.
Action: search_csv
Action Input: ""Search for the CO2 henry coefficient of XEGKUR at 298 K in mol/cm3Pa""
Observation: There is no information about the CO2 Henry's coefficient at 298 K of XEGKUR.
Thought: The search_csv tool did not provide any information on the CO2 henry coefficient of XEGKUR at 298 K in mol/cm3Pa. I need to find another way to obtain this information.
Action: predictor
Action Input: "Predict the CO2 henry coefficient of XEGKUR at 298 K in mol/cm3Pa"
Observation: The predicted CO2 Henry's coefficient at 298 K for XEGKUR is -3.62769 mol/Kg·Pa. However, this is a logarithmic value. To get the original value, an exponential must be applied.
Thought: I need to convert the logarithmic value to the original value.
Action: Python_REPL
Action Input: "import math\nprint(math.exp(-3.62769))"
Observation: 0.026577507595890823
Thought: I have the original value of the CO2 henry coefficient of XEGKUR at 298 K in mol/cm3Pa.
Final Answer: The CO2 henry coefficient of XEGKUR at 298 K in mol/cm3Pa is approximately 0.027.

Figure S15. ChatMOF's response with fine-tuned Language Model (LLM) to the question "Provide the CO_2 henry coefficient of XEGKUR at 298 K in mol/cm^{3}Pa" compared with GPT-4. The text highlights the inefficient or incorrect process generated by ChatMOF in red, while the correct process is highlighted in blue. Tested on January 31, 2024, at 03:42 KST.

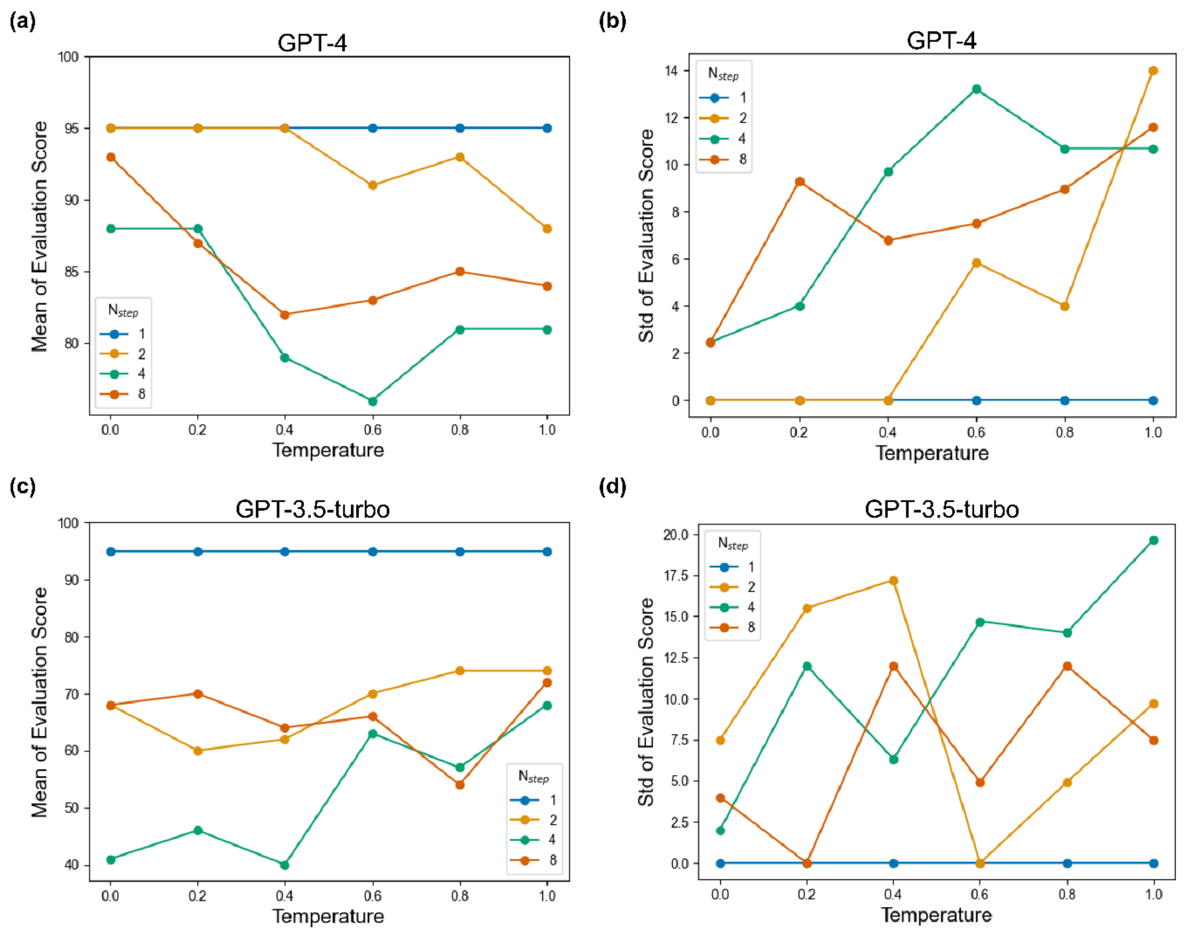

The evaluation score is explained in Supplementary Note S16. N_{step} refers to the number of tools executed by ChatMOF for each question. The experiment was conducted five times at each temperature, and the mean and standard deviation were calculated from the results. (a) Mean evaluation score for GPT-4 at different temperatures. (b) Standard deviation of the evaluation score for GPT-4. (c) Mean evaluation score for GPT-3.5-turbo. (d) Standard deviation of the evaluation score for GPT-3.5-turbo. Tested on January 28, 2024.

(a) Lexical Diversity (TTR)

GPT-4	GPT-3.5	Template-based
0.1149	0.0782	0.1125

(b) Diversity of embedding vectors

GPT-4	GPT-3.5	Template-based
1.022	0.897	0.9628

(c)

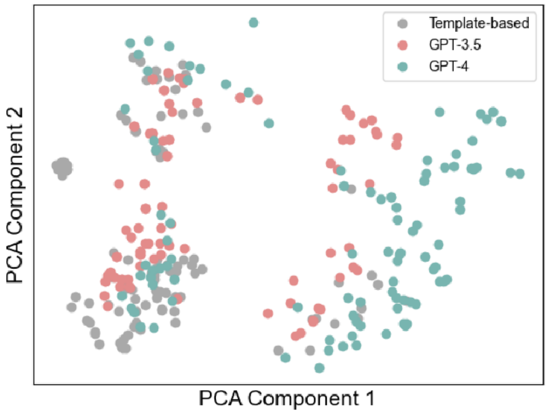

Figure S17. Comparison of Question Diversity between GPT-4, GPT-3.5-turbo, and Template-based Method. (a) Lexical diversity of questions. (b) Diversity of embedding vectors using the text-embedding-small model (OpenAI). (c) Principal Component Analysis (PCA) graphs representing the distribution of embedding vectors for each method.

What is the largest cavity diameter of ASOVEL_clean?
What's the pore limiting diameter of XEXLUJ_clean?
How large is the free pore diameter for DIBQUC?
What's the density of ROLCEC19?
Can you tell me the accessible surface area of ALIBUT?
What is the non-accessible surface area of ARAHIM02?
What is the accessible volume fraction for EREGOY_clean?
Can you tell me about the accessible volume of UZANOZ?
What's the non-accessible volume for NAHFOU?
What type of metal is present in PITPEP_clean?
Does DUBKAO have an open metal site?
Can you tell me the type of open metal in FECYUJ?
Can you provide the top 5 materials with the highest accessible surface area?
Which materials have the highest non-accessible volume?
What's the largest cavity diameter in materials with a density greater than a 7.0?
Can you provide a list of materials with an open metal site?
What's the accessible volume fraction for materials with a density less than a 0.25?
How does the pore limiting diameter of YUSGID_clean compare with other materials?
What materials have the largest free pore diameter?
Is there an open metal site in NELVAC?
Can you provide a list of materials with the highest pore limiting diameter?
What materials have the largest accessible volume?
What's the non-accessible surface area for WAWGOQ_clean?
What is the type of open metal in YUNJIB?
Can you list the top 10 materials with the highest non-accessible surface area?
How high is the accessible surface area for JUKPAI_clean?
What's the largest cavity diameter in materials with a density less than a 0.8?
Can cg901114e_si_002 have an open metal site?
What materials have the largest accessible volume fraction?
What is the pore limiting diameter of LOLREL?
How does the accessible volume of OSIXEU compare with other materials?
Can you provide the top 10 materials with the highest density?
What's the largest free pore diameter in materials with a density less than a 0.8?
How high is the non-accessible volume for FIJDIM05?
What materials have the largest cavity diameter?
What is the metal type in FOTNIN?
How high is the accessible volume fraction for WOBCAT?
Can you list materials with a pore limiting diameter greater than a 6.0?
What is the density of materials with an accessible surface area above a 3000 m2/g?
What is the type of open metal in IYAFIX?
Can JILXOS have an open metal site?
What materials have the largest non-accessible surface area?

Can you list the top 5 materials with the highest largest free pore diameter?
What is the accessible volume for MOJJUR?
Can you provide the top 10 materials with the highest largest cavity diameter?
What is the accessible surface area for materials with a pore limiting diameter below a 2.0?
What is the non-accessible volume for WABTOK?
What materials have the largest pore limiting diameter?
What's the type of open metal in QOWRAV11?
Can PODVUB have an open metal site?
Can you list the top 10 materials with the highest accessible volume fraction?
What is the metal type in QULMIU?
How does the non-accessible surface area of YICTUZ compare with other materials?
Can you provide the top 10 materials with the highest accessible volume?
What's the pore limiting diameter in materials with a density greater than a 2.5?
How high is the largest free pore diameter for DUFKAS?
What materials have the largest density?
What is the largest cavity diameter for TONTIB_clean?
Can you list the top 5 materials with the highest non-accessible volume?
What is the accessible surface area for UXUPEJ?
What is the density of NATXIR?
How does the largest free pore diameter of LITDAV compare with other materials?
Can you provide the top 10 materials with the highest largest free pore diameter?
What's the pore limiting diameter for CSMNOX?
How high is the non-accessible surface area for MUZKAV_clean?
What materials have the largest accessible surface area?
What's the type of open metal in cm503311x_F-mil-53-Al-lp-mono?
Can TAHYUZ have an open metal site?
Can you list the top 5 materials with the highest density?
What is the metal type in YUBTUM?
How does the accessible surface area of TATFOL compare with other materials?
Can you provide the top 5 materials with the highest accessible surface area?
What's the largest cavity diameter for NELVAC?
How high is the accessible volume for EPUJIJ_clean?
What materials have the largest non-accessible surface area?
What's the type of open metal in WAWGOQ_clean?
Can YUNJIB have an open metal site?
Can you list the top 5 materials with the highest accessible volume fraction?
What is the metal type in JUKPAI_clean?
How does the non-accessible surface area of HUZFIS compare with other materials?
Can you provide the top 5 materials with the highest largest cavity diameter?
What's the pore limiting diameter for XIPTAS_clean?
How high is the largest free pore diameter for LOLREL?

What materials have the largest density?
What is the largest cavity diameter for YAVVUP?
Can you list the top 5 materials with the highest non-accessible volume?
What is the accessible surface area for FIJDIM05?
What is the density of TUFTAR?
How does the largest free pore diameter of FOTNIN compare with other materials?
Can you provide the top 5 materials with the highest largest free pore diameter?
What's the pore limiting diameter for LAXGOH02?
How high is the non-accessible surface area for GEDQOX?
What materials have the largest accessible surface area?
What's the type of open metal in JILXOS?
Can VAHSON have an open metal site?
Can you list the top 5 materials with the highest density?
What is the metal type in MOJJUR?
How does the accessible surface area of REHVEH_clean compare with other materials?
Can you provide the top 5 materials with the highest accessible surface area?
What's the largest cavity diameter for NELVAC?

Table S1. 100 questions in search task.

At 77 K, what's the hydrogen uptake for ZAXQOG_clean at 100bar?
Under standard temperature conditions of 298K and dilute system, can you list the materials with the highest O2 diffusivity?
Is XEGKUR_clean thermally stable?
What's the thermal stability of QINSUD_clean?
Which materials have a bandgap between 1 and 2 eV?
At room temperature (298K), what's the CO2 Henry coefficient for XEGKUR_clean?
Can you provide the top 10 materials with the highest N2 diffusivity at 298K and infinity dilute system?
Is the solvent removal stability of XEGKUR_clean strong?
Can you list 5 materials with a bandgap near 1.6 eV when evaluated at 298K and 1bar?
Under 77K and infinity dilute conditions, what's the hydrogen diffusivity for NISPEL_charged?
What materials show the best performance in hydrogen uptake at 100bar considering a temperature of 77 K?
How high is the O2 uptake of LOQLIN_SL at 298K and normal pressure?
Can XAVDUV_clean stably store hydrogen under 100bar pressure when observed at 77K?
At 298K and standard atmospheric pressure, how high is the N2 uptake for UXABUR_clean?
What's the O2 diffusivity for OCUVUF_clean under conditions of 298K and dilute system?
Is XAHWAG_clean stable when the solvent is removed?
What are the top 5 materials with the highest CO2 Henry coefficient at room temperature?
At 298K and dilute system, how high is the N2 diffusivity for XEGKUR_clean?
What materials are known for their high O2 uptake at a temperature of 298K and 1 bar pressure?
How high is the bandgap for XEGKUR_clean?
What materials have the highest thermal stability?
Can you show materials with a bandgap greater than 3.0 eV?
At 298K and standard atmospheric pressure, how much O2 can XEGKUR_clean take up?
What is the CO2 Henry coefficient for materials with bandgap less than 1.5 eV at 298K?
At 77K, how does the hydrogen uptake of OCUVUF_clean compare with other materials at 100bar?
Can you list materials with hydrogen diffusivity greater than 0.1 cm2/s at 77K and dilute system?
What is the N2 diffusivity in XEGKUR_clean under 298K and dilute conditions?
What is the thermal stability of materials with bandgap above 2.5 eV?
Which materials demonstrate a high level of O2 diffusivity at 298K and dilute condition?
How does the N2 uptake of XEGKUR_clean compare with others at 298K and 1 bar?
Can QINSUD_clean remove solvents stably?
How high is the thermal stability for NISPEL_charged?
At 298K and 1 bar pressure, what is the O2 uptake of MIFROK_clean?
Under standard conditions of 298K and 1 bar, which materials have the highest N2 uptake?
Can you list materials that show solvent removal stability?
How does the hydrogen diffusivity for QINSUD_clean compare with others at 77K and dilute system?
Under conditions of 298K, what is the CO2 Henry coefficient for WOYJOL_clean?

What materials have the best N2 diffusivity when measured at 298K and dilute system?
Is the bandgap of XEGKUR_clean within the range 0.5-1.5 for photovoltaic applications?
At standard conditions of 77K and 100 bar, which materials have the highest hydrogen uptake?
How high is the bandgap for XEGKUR_clean when measured at 298K and under a pressure of 1 bar?
Can you provide a list of materials with high thermal stability?
At 77K and 100bar pressure, what is the hydrogen uptake of OCUVUF_clean?
What is the solvent removal stability of XAHWAG_clean?
Can GUCJAQ_clean diffusively handle N2 when evaluated at 298K and dilute system?
What materials show high O2 uptake capabilities under conditions of 298K and 1bar?
At 298K and dilute system, what is the diffusivity of O2 in XEGKUR_clean?
How much N2 can DUVNIS01_charged take up at 298K and 1 bar?
Under conditions of 298K, which materials have a high CO2 Henry coefficient?
How much hydrogen can XEGKUR_clean take up at 100bar pressure when observed at 77 K?
Under 298K and dilute conditions, how high is the O2 diffusivity for DUVNIS01_charged?
At standard temperature (298K) and 1 bar pressure, can IZEHAX_clean hold a high volume of N2?
At 298K and 1bar, how stable is JALCAD_clean when the solvent is removed?
What is the thermal stability of COKMUM_clean?
Can AYOYOE_clean handle a high volume of O2 when tested at 298K and 1 bar?
At 298K and dilute system, what is the diffusivity of N2 in IHAJIL_clean?
What materials show a high level of N2 diffusivity under conditions of 298K and 1 bar pressure?
Is the bandgap of XEGKUR_clean higher or lower than 1.5 eV?
At a temperature of 298K and a pressure of 1 bar, how does the O2 uptake of DUVNIS01_charged compare with others?
Under standard ambient temperature (298K), what is the CO2 Henry coefficient for XEGKUR_clean?
At 77K and 100 bar, can DUVNIS01_charged take up a significant volume of hydrogen?
Considering conditions of 298K and 1 bar, how much N2 can IZEHAX_clean hold?
At 77 K and dilute system, what is the hydrogen diffusivity for JALCAD_clean?
Can COKMUM_clean remain stable when the solvent is removed?
Which materials exhibit the best O2 diffusivity at 298K and dilute system?
How high is the bandgap for XEGKUR_clean?
What is the thermal stability of DUVNIS01_charged?
Under a temperature of 298K and pressure of 1 bar, how high is the O2 uptake for IZEHAX_clean?
At 298K and standard pressure, what materials have the highest N2 uptake?
Can you list the top materials that demonstrate thermal stability?
Given the conditions of 298K and dilute condition, what is the N2 diffusivity for QINSUD_clean?
What materials are thermally stable?

For a metal-organic framework at 298K and 1 bar, what is the CO2 Henry coefficient for KUGQIN_clean?
Can you provide the materials with the highest O2 diffusivity when measured at 298K and dilute system?
What materials have a high bandgap?
Considering 77K and 100bar conditions, how high is the hydrogen uptake of XEGKUR_clean?
Under ambient conditions of 298K and 1 bar, what is the N2 uptake for DUVNIS01_charged?
Can OCUVUF_clean remove solvents without compromising its stability?
At a standardized temperature of 77K and dilute system, how does XAHWAG_clean's hydrogen diffusivity measure?
What is the O2 uptake of GUCJAQ_clean at the typical conditions of 298K and 1 bar?
Can GAJTUI_clean take up a significant volume of N2 under conditions of 298K and 1 bar?
How stable is JEDJUY_clean at elevated temperatures?
At standard ambient conditions, which materials exhibit a high CO2 Henry coefficient?
Under the set conditions of 77K and dilute, how high is the hydrogen diffusivity of XEGKUR_clean?
What is the solvent removal stability of DUVNIS01_charged?
At 298K and a infinity dilute condition, how does OCUVUF_clean's N2 diffusivity compare to other materials?
Which materials show a pronounced hydrogen uptake at 77K and 100 bar?
Can XEGKUR_clean stably take up a large volume of O2 when examined at 298K and 1 bar?
What is the thermal stability of DUVNIS01_charged?
At standard conditions (298K and 1 bar), which materials are known for high O2 uptake?
Can XEGKUR_clean stably remove solvents?
How high is the bandgap of DUVNIS01_charged?
Which materials exhibit the highest N2 diffusivity at a temperature of 298K and infinity dilute condition?
Under conditions of 298K and 1 bar, how elevated is the N2 uptake for XEGKUR_clean?
At the standardized 298K and infinity dilute system, what is the O2 diffusivity of DUVNIS01_charged?
Under standard conditions, how high is the CO2 Henry coefficient for IZEHAX_clean?
At 77K and 100 bar pressure, can JALCAD_clean stably accommodate a large volume of hydrogen?
Under the given conditions of 77K and dilute system, which materials have the highest hydrogen diffusivity?
Is XEGKUR_clean thermally stable?
Can you provide a list of materials with a bandgap exceeding 2 eV?

Table S2. 100 questions in prediction task.

Can you generate the structures with the largest surface area?
I'm looking for structures with a hydrogen uptake of about 500 cm3/cm3 at 100bar and 77K, can you generate those?
Can you generate structures with a density close to 0.4?
What structures can you generate with a smallest accessible pore volume?
I'm interested in structures with a low O2 uptake at 298K and 1bar, can you generate these?
I'm curious about structures where accessible volume fraction is less than 0.4, can the model generate these?
Can the model generate structures where the largest cavity diameter is in the bottom quartile?
I am curious about structures where the thermal stability is the highest among all, can you generate those?
What structures can be generated with a N2 uptake in the 3rd quartile range?
Can you create a MOF with a void fraction greater than 0.5 that has the highest CO2 Henry coefficient at 298 K?

Table S3. 10 questions in generation task

	Model name	Llama-2-chat-7B-hf	Llama-2-chat-13B-hf
Search task	True	17	8
	False (Agent)	11	11
	False (Evaluation)	10	2
	False (Plan)	10	6
	False (Code)	45	67
	False (Token)	7	6
Prediction task	True	0	0
	False (Agent)	26	3
	False (Evaluation)	13	11
	False (Plan)	31	22
	False (Model Selection)	3	20
	False (Code)	21	37
	False (Token)	6	7

Table S4. Accuracy for search and prediction task using Llama-2-chat-7B-hf and Llama-2-chat-13B-hf model (tested on 23.11.10)

References

1 Touvron, H. *et al.* Llama: Open and efficient foundation language models. Preprint at https://arxiv.org/abs/2302.13971 (2023)

2 Touvron, H. *et al.* Llama 2: Open foundation and fine-tuned chat models. *arXiv preprint arXiv:2307.09288* (2023).

3 Roziere, B. *et al.* Code llama: Open foundation models for code. *arXiv preprint arXiv:2308.12950* (2023).

4 Shen, Y. *et al.* Hugginggpt: Solving AI tasks with ChatGPT and its friends in huggingface. Preprint at https://arxiv.org/abs/2303.17580 (2023)

5 Wei, J. *et al.* Finetuned language models are zero-shot learners. *International Conference on Learning Representations* (2022).

6 Ziegler, D. M. *et al.* Fine-tuning language models from human preferences. Preprint at https://arxiv.org/abs/1909.08593 (2019)

7 Dunn, A. *et al.* Structured information extraction from complex scientific text with fine-tuned large language models. Preprint at https://arxiv.org/abs/2212.05238 (2022)

8 Karpas, E. *et al.* MRKL Systems: A modular, neuro-symbolic architecture that combines large language models, external knowledge sources and discrete reasoning. Preprint at https://arxiv.org/abs/2205.00445 (2022)
