# Supplementary material for: ChatMOF: an artificial intelligence system for predicting and generating metal-organic frameworks using large language models
Source: Nat Commun. 2024 Jun 3;15:4705. doi: 10.1038/s41467-024-48998-4 (PMC11148193; doi:10.1038/s41467-024-48998-4)
Supplement: Supplementary file 3 — Description of Additional Supplementary Files [file 41467_2024_48998_MOESM3_ESM.pdf]

File Name: Supplementary Data 1
Description:

Zip file containing raw data for ChatMOF transcripts used in Figure 2, 3, 4, and 5, as well as Supplementary Notes S1, S2, S3, S4, and S5.

Supplementary Data 1.zip

- Figure 3.txt
- Figure 4.txt
- Figure 5 & Supplementary Note 1.txt
- Figure 6.txt
- Supplementary Figure S2.txt
- Supplementary Figure S3.txt
- Supplementary Figure S4.txt
- Supplementary Figure S5.txt

+++++++++++++++

File Name: Source Data 1
Description:

Zip file containing raw data for the properties of the generated MOF in the generate task using GPT-4

Source Data 1.zip

- Figure 7(a) – initial.csv
- Figure 7(a) – final.csv
- Figure 7(b) – initial.csv
- Figure 7(b) – final.csv
