# Supplementary material for: ChatMOF: an artificial intelligence system for predicting and generating metal-organic frameworks using large language models
Source: Nat Commun. 2024 Jun 3;15:4705. doi: 10.1038/s41467-024-48998-4 (PMC11148193; doi:10.1038/s41467-024-48998-4)
Supplement: Supplementary file 2 — Peer Review File [file 41467_2024_48998_MOESM2_ESM.docx]

|  |  |
| --- | --- |

| **Author Checklist** | NCOMMS-23-38310B |
| --- | --- |
| 0000000000000000000000000000000000000000000000000000000000000 | 0000000000000000000000000000000000000 |
| **Please check the items below carefully and add a response in each row of the table to indicate the changes that you have made. Please also check through any additional marked-up edits we may have provided within the manuscript file.** | |
|  |  |
| 1. Since we are a commercial publisher, you may need to find an alternative to the image used below:  Free for non-commercial use: https://www.iconfinder.com/icons/49597/ordering_table_icon  2. Where you have used the image below (in Fig 1), please attribute/give credit within the appropriate figure legend according to the license.  Creative Commons (Attribution 3.0 Unported)  https://www.iconfinder.com/icons/653262/bulb_light_electric_energy_idea_lamp_thought_icon | We have changed the non-commercial and creative common 3.0 icons to the basic license icon in Figure 1(b).  - Bulb (basic license): <https://www.iconfinder.com/icons/2757666/bulb_idea_lamp_light_icon>  -Table (basic license): <https://www.iconfinder.com/icons/2305746/glass_lookup_magnifying_table_icon>  Since we changed to a BASIC license, we didn't specify it in the caption. |
| 3. Supplementary Data 2 has been mentioned in the legend for Fig.4. I'm assuming this is a typo since no Supplementary Data 2 has been uploaded, please amend this. | We have checked and found that Figure 4 is labeled Supplementary Note 1. We found that Figure 7 has supplementary data2, which we converted to Source data1. |
| 4. Please remove subjective or exaggerated language, such as "impressive", from the abstract and the main text. | We have the word “impressive” to objective language:  ChatMOF is an artificial intelligence (AI) system that is built to predict and generate metal-organic frameworks (MOFs). By leveraging a large-scale language model (GPT-4, GPT-3.5-turbo, and GPT-3.5-turbo-16k), ChatMOF extracts key details from textual inputs and delivers appropriate responses, thus eliminating the necessity for rigid and formal structured queries. The system is comprised of three core components (i.e. an agent, a toolkit, and an evaluator) and it forms a robust pipeline that manages a variety of tasks, including data retrieval, property prediction, and structure generations. ChatMOF shows high accuracy rates of 96.9% for searching, 95.7% for predicting, and 87.5% for generating tasks with GPT-4. Additionally, it successfully creates materials with user-desired properties from natural language. The study further explores the merits and constraints of utilizing large language models (LLMs) in combination with database and machine learning in material sciences and showcases its transformative potential for future advancements.  Additionally, we have checked for the use of subjective words in other paragraphs. |
|  |  |
|  |  |
| **Remaining reviewer comments** |  |
| Our guidance: | Your response: |
| Please consider following the suggestions of Reviewer #5 regarding small changes in the code repository. | We made changes to the github repository as requested by the reviewer. The modifications were written in a point-by-point response to the reviewer's comment. |
|  |  |
| **Abstract and editor's summary** |  |
| Our guidance: | Your response: |
| The abstract — which should be roughly 150-200 words long and contain no references — should serve both as a general introduction to the topic and a non-technical summary of your main results and their implications. It should contain a brief account of the background and rationale of the work, followed by a statement of the main conclusions introduced by the phrase 'Here we show' or some equivalent phrase. Because we hope that researchers in a wide range of disciplines will be interested in your work, the abstract should be as accessible as possible, explaining essential but specialised terms concisely. We encourage you to show your abstract to colleagues outside of your direct field of expertise to uncover any problematic concepts. | We have modified the abstract.  ChatMOF is an artificial intelligence (AI) system that is built to predict and generate metal-organic frameworks (MOFs). By leveraging a large-scale language model (GPT-4, GPT-3.5-turbo, and GPT-3.5-turbo-16k), ChatMOF extracts key details from textual inputs and delivers appropriate responses, thus eliminating the necessity for rigid and formal structured queries. The system is comprised of three core components (i.e. an agent, a toolkit, and an evaluator) and it forms a robust pipeline that manages a variety of tasks, including data retrieval, property prediction, and structure generations. ChatMOF shows impressive accuracy rates of 96.9% for searching, 95.7% for predicting, and 87.5% for generating tasks with GPT-4. Additionally, it successfully creates materials with user-desired properties from natural language. The study further explores the merits and constraints of utilizing large language models (LLMs) in combination with database and machine learning in material sciences and showcases its transformative potential for future advancements. |
|  |  |
| **Author information** |  |
| Our guidance: | Your response: |
| We ask that you consult with your coauthors to ensure that all names, affiliations, and titles are represented correctly. Note that if any authors are added or removed after this point then all authors will be requested to provide approval documentation that could potentially delay the production of your paper. | All authors have given approval for the complete author list and confirmed all names, affiliations, and titles. |
| Ensure affiliations are appropriately labelled, written in English, and featured sequentially and in order (1,2,3,... or a,b,c...). Please ensure all corresponding authors are marked with a specific symbol and include their emails. Please also use a specific symbol for equally contributing authors if relevant. | The affiliations have been labelled and featured sequentially in order. Additionally, the corresponding author and email address have been added to the first page of the manuscript, marked in red font. |
| Please ensure the author contributions section mentions each author's initials at least once with their contributions to the work. Authors with the same initials must be differentiated in the statement. | We have verified that all authors' initials are mentioned at least once along with their contributions. |
|  |  |
| **Article structure** |  |
| Our guidance: | Your response: |
| We can accommodate up to 10 display items (Figures or Tables) in the main article. Each Figure and Table must fit easily within an A4 page (210 x 297 mm). Please ensure that the number and size of your Figures and Tables fulfil these requirements to avoid any delay in the acceptance of your article. | We have checked that all figures are under A4 size (210 * 297 mm). |
| **To comply with this format and optimise the presentation of data in your Article, we suggest the following changes to the display items in your paper:** |  |
| *When scaled to fit our article template, text and detail in Figures 5 and 6 may be too small to be legible. Please provide your final figures at article scale and as a guideline, ensure that text is not smaller than 6 pts.* | We have increased the font and figure size in figures 5 and 6. |
| **Please ensure your main manuscript file includes the following sections, in this order:** | We have changed the order to match the guidelines. |
| *Title*  *Author list*  *Affiliations*  *Abstract*  *Introduction*  *Results*  *Discussion (optional)*  *Results and Discussion (optional)*  *Methods*  *Data Availability*  *Code Availability (if relevant)*  *References*  *Acknowledgements*  *Author Contributions Statement*  *Competing Interests Statement* |  |
| We do not edit Supplementary Information files; they will be uploaded with the published article as they are submitted with the final version of your manuscript. Any tracked changes should be removed from the file and the file should be provided as a PDF file. Supplementary Figures do not need to be provided separately. |  |
|  |  |
| Please supply Source Data files for all data presented in graphs within the Figures. We are aware there are tools that can extract values from images, but these hinder both accessibility and accuracy of the extracted data, therefore we require authors to also provide the underlying data for all plots/graphs in the manuscript and supplementary information. | We have labelled the data in Figure 7 with the number of data for each label. Additionally, the raw data for Figure 7(a) and 7(b) has been included in Supplementary Data 2.  We have added the following sentence in Caption of Figure 7:  The data used to plot the violin is provided in Supplementary Data 2. |
|  |  |
| **Main text** |  |
| Our guidance: | Your response: |
| Please refrain from using words such as new/novel/first, when referring to the scientific findings. Please also remove exaggerated or subjective language such as 'extremely'/'outstanding', 'innovative', 'ingenious', etc. | We have removed subjective language from the text such as first/outstanding/innovative. |
| Please do not use italics, bold font, underlining or speech marks/quotation marks except in headings unless required for technical terms (in both the main text and the display items). | We checked that there are no italics, bold font in the body, and no quotation marks except where necessary (e.g., questions in ChatMOF). |
| Please make sure that mathematical terms throughout your manuscript and Supplementary Information (including in figures, figure axes, and legends) conform strictly to the following guidelines. Equations must be supplied in editable format, and not as images. Scalar variables (e.g. x, V, χ) must be typeset in italic, whereas multi-letter variables and functions (e.g. log) must be formatted in roman. Vectors (such as the wavevector k or the magnetic field vector B) must be typeset in bold without italics. | We have verified that mathematical terminology adheres to the guidelines. |
| We encourage to use consistently exponential notation to define units, e.g. kcal mol⁻¹ instead of kcal/mol, throughout your text and Figures. Please feel free to not follow this request if that is in conflict with the output format of ChatMOF. | We think it's important to match ChatMOF's output format, so we're keeping the current unit system in the figures and text. We think it's better to match the actual results of the run than to standardize the units. |
|  |  |
| **Figures and Tables** |  |
| Our guidance: | Your response: |
| Please see the guidelines linked below for detailed instructions about how your figures should be prepared. Following these instructions will reduce the chances of delays should we need to request replacement artwork from you at a later stage. | We have verified that the Figure follows the guideline. |
| <https://www.nature.com/documents/NRJs-guide-to-preparing-final-artwork.pdf> |  |
| Wherever statistics have been derived (e.g. error bars, box plots, statistical significance) the legend needs to provide and define the n number (i.e. the sample size used to derive statistics) as a precise value (not a range). | In the violin plots in Figures 8 and S10, we have given the number of each data point as a number in parentheses, and also mentioned in the caption that the number in parentheses is the number of data points.  The number in parentheses represents the number of data. |
| **8, S10** |  |
| All error bars need to be defined in the legends (e.g. SD, SEM) together with a measure of centre (e.g. mean, median). For example, the legends should state something along the lines of “Data are presented as mean values +/- SEM” as appropriate. All box plots need to be defined in the legends in terms of minima, maxima, centre, bounds of box and whiskers and percentile. | We have added the following sentences in Caption of Figure 8 and Figure S10  The inner box represents the interquartile range (IQR) and the white horizontal line inside the box indicates the median of the distribution. |
| **8, S10** |  |
| Please make sure that the legends of all Figures can be understood without needing to refer to the main text. In particular, any abbreviations, symbols or colours present in your figures must be defined in the associated legends. See below for specific guidance. | We have written the legends for the figures so that they can be understood without referring to the main text. For abbreviations, we have mentioned the exact meaning in the caption. |
| We suggest you to consider removing Figure 1 altogether, or merging it with Figure 2 - at this point, Figure 1 is quite simple and provides a limited amount of information. | We have combined Figure 1 and Figure 2 to Figure 1 (a and b). |
| Please extend the legend of Figure 2 to give some more details on the meaning of "agent", "toolkit" and "evaluator": which kind of software each of these is, etc. | We have modified Figure 2 to provide additional details on the agent, toolkit, and evaluator. We also added the label 'LLM' in agent, and evaluator to indicate that the they are composed of LLM. |
| In Figure 7 and all the similar Supplementary Figures, please define the accuracy metrics and failure cases (logic error vs token limit exceeded). | We have added the following sentences in the caption of Figure 7 and Supplementary Figure S6, S8  False (Exceed Token Limit) indicates that the LLM has exceeded the maximum token allowance, while False (Logic Error) indicates that the ChatMOF's logic has resulted in an incorrect response or anomaly. |
|  |  |
| **Data and Code** |  |
| Our guidance: | Your response: |
| Nature journals strongly support public availability of data and code. Please deposit the data and code used in your paper into a public data repository, or alternatively, present the data as Supplementary Information. If data can only be shared on request, please explain why in your Data Availability Statement, and also in the correspondence with your editor.  Please note that for some data types, deposition in a public repository is mandatory. Any restrictions on sharing of these data types must be clearly indicated in the statement and discussed with the editor. More information on our data deposition policies and available repositories can be found here: | We uploaded the data through github and figshare and informed data sharing through data availability.  Procedures and answers to questions in LLM-specific ChatMOF (GPT-4, GPT-3.5-turbo, GPT-3.5-turbo-16k, Llama-2-7B-chat-hf, Llama-2-13B-chat-hf) are available in the experimental section of the GitHub repository under https://github.com/Yeonghun1675/ChatMOF.git. The fine-tuned model used in ChatMOF is available at https://figshare.com/articles/dataset/Fine-tuned_MOFTransformer_model/24288919, and the hMOF used in the genetic algorithm is available at https://figshare.com/articles/dataset/Database_for_ChatMOF/24287731. |
| <https://www.nature.com/nature-research/editorial-policies/reporting-standards#availability-of-data> |  |
| All published manuscripts reporting original research in Nature Portfolio journals must include a data availability statement, within the Methods and under the heading 'Data Availability'.  The data availability statement must make the conditions of access to the “minimum dataset” that are necessary to interpret, verify and extend the research in the article, transparent to readers.  This minimum dataset may be provided through deposition in public community/discipline-specific repositories, custom proprietary repositories or general repositories like Figshare, Zenodo and Dryad. Providing large datasets in supplementary information is strongly discouraged and the preferred approach is to make data available in repositories. Scientific Data, a Nature Portfolio journal, maintains a list of approved and recommended data repositories to support researchers seeking suitable repositories for their data (https://www.nature.com/sdata/policies/repositories).  The Data Availability Statement should also reference any source data published alongside the paper.  If DOIs are provided, we also strongly encourage including these in the Reference list (authors, title, publisher (repository name), identifier, year).  For clinical datasets or third party data, please ensure that the statement adheres to our policy (https://www.nature.com/nature-research/editorial-policies/reporting-standards#availability-of-data) | We uploaded the data through github and figshare and informed data sharing through data availability.  Procedures and answers to questions in LLM-specific ChatMOF (GPT-4, GPT-3.5-turbo, GPT-3.5-turbo-16k, Llama-2-7B-chat-hf, Llama-2-13B-chat-hf) are available in the experimental section of the GitHub repository under https://github.com/Yeonghun1675/ChatMOF.git. The fine-tuned model used in ChatMOF is available at https://figshare.com/articles/dataset/Fine-tuned_MOFTransformer_model/24288919, and the hMOF used in the genetic algorithm is available at https://figshare.com/articles/dataset/Database_for_ChatMOF/24287731. |
| Please use the following template to provide all the information stated above:  The XX data generated in this study have been deposited in the YY database under accession code ZZ [add hyperlink here]. The XX data are available under restricted access for {insert reason}, access can be obtained by {explain how}. The raw XX data are protected and are not available due to data privacy laws. The processed XX data are available at YY. The XX data generated in this study are provided in the Supplementary Information/Source Data file. The XX data used in this study are available in the YY database under accession code ZZ [Add hyperlink here]. | - The procedures and answers to question in ChatMOF data generated in this study have been deposited in the github database under accession code ChatMOF  (https://github.com/Yeonghun1675/ChatMOF.git.).  - The Fine-tuned MOFTransformer model used in ChatMOF is deposited in the Figshare database under accession code  <https://figshare.com/articles/dataset/Fine-tuned_MOFTransformer_model/24288919>  - The hMOF used in the genetic algorithm is deposited un the Figshare database under accession code  https://figshare.com/articles/dataset/Database_for_ChatMOF/24287731. |
| When electronic structure calculations are reported in the manuscript, the atomic coordinates of the optimised computational models should be provided. For molecular dynamics trajectories at least the initial and final configurations should be supplied. We encourage you to make them available by uploading the structures in any of the existing data repositories (see https://www.springernature.com/gp/authors/research-data-policy/recommended-repositories). Alternatively, they can be supplied as a separate Supplementary Data file (ideally as a plain, unformatted text file) | Electronic structure calculations are not provided in our manuscript. |
| We notice that you have deposited your code in a Github repository, which we fully support. We strongly encourage you in addition to make your code citable by obtaining a DOI for the Github repository in order to provide a permanent reference to the version of the code used in this study and improve reproducibility. This can be done by linking the repository to Zenodo, following the instructions here: https://guides.github.com/activities/citable-code/ Please cite the Github repository in your manuscript text or Code Availability statement and in your reference list: authors, title (this paper), repository name, DOI identifier, year. Alternatively, you can deposit the code in Gigantum or Code Ocean for the same purpose. | We published our github code on zenodo and cited it in the manuscript.  DOI : 10.5281/zenodo.10806289 |
| Thank you for providing the transcripts of ChatMOF as part of the supplementary information. However, having these in a pdf makes it a bit difficult to extract the data for reusability. We suggest that you add raw text files as Supplementary Data files. | We have provided the raw file of the ChatMOF transcript provided in the manuscript and supporting information as Supplementary data 1.  (Figure 2, 3, 4, 5, Supplementary Note S1, Supplementary Figure S2, S3, S4, and S5)  We have added the following sentence in Caption of Figure 4 and 5:  The full process of the example is provided in Supplementary Note S1 and Supplementary Data 1.  The full process of the example is provided in Supplementary Data 1. |
|  |  |
| **Methods** |  |
| Our guidance: | Your response: |
| Sufficient details of the experiments must be provided in the Methods section such that they could be reproduced without reference to published papers. Use of the term "as described previously" is not encouraged. | We have checked the method sections contains sufficient details. |
|  |  |
| **References** |  |
| Our guidance: | Your response: |
| All references must be cited in numerical order. The reference list will be formatted according to the Nature style by our journal production team, however please ensure that references contain all of the information required, eg:  Kurumada, S., Takamori, S. & Yamashita, M. An alkyl-substituted aluminium anion with strong basicity and nucleophilicity. <i>Nat. Chem.</i> <b>12</b>, 36–39 (2020). | We have checked that the reference followed the guideline. |
| Supplementary References should appear at the end of the Supplementary Information file, and must be self-contained and numbered from 1. References mentioned in both the main text and the Supplementary Information should be part of both reference lists so that the Supplementary Information does not refer to the reference list in the main paper and vice versa. | We have checked that the Supplementary reference followed the guideline. |
|  |  |
| **End matter** |  |
| Our guidance: | Your response: |
| Nature Portfolio defines Competing Interest (CI) as financial and non-financial interests (including but not limited to funding, employment, stocks, shares, patents, personal or professional relationships with individuals or institutions, and unpaid membership advocacy) that could be perceived to directly undermine the objectivity, integrity, and value of a publication, or could be seen as having an influence on the judgments and actions of authors with regard to objective data presentation, analysis, and interpretation.  Please thoroughly review our policy on Competing Interests and include a detailed statement both in your final manuscript file and in our manuscript tracking system. Please ensure the statements are identical in both. Be specific about how each point stated relates to the research and list applicable author initials, and/or patent numbers.  If there are no competing interests, a negative statement must be included. | We have checked the competing interests section. |
| <https://www.nature.com/nature-research/editorial-policies/competing-interests> |  |
| Please confirm that all relevant funding awarded to each author is described in the Acknowledgements section. List each grant number, followed by the initials of the author who received it. | We have checked the acknowledgements section. |
|  |  |
| **Preparing your manuscript files** |  |
| Our guidance: | Your response: |
| Unless otherwise stated please limit individual file sizes to approximately 20MB. We strongly encourage the use of repositories for large datasets or source data due to size considerations. | We have checked that all individual file sizes are under 20 MB. |
| Please supply a brief (maximum 250 characters {not words}, including spaces) summary of the main findings of the paper to be used on our website and in our e-alerts. The summary should be written in the third person in language suitable for a broad audience. It should follow the structure: "[[Brief background first sentence]]. Here, authors [[brief description of accomplishment]]." The summary may be edited by the editors prior to publication. Please provide this summary here. | Recently, there has been a growing interest in applying LLMs in the field of materials. Here, authors have developed an artificial intelligence system called ChatMOF, which is designed to predict and generate metal-organic frameworks using LLMs. |
| Please note that all Supplementary Information must be provided as a single separate PDF file, not within the manuscript file.  All Supplementary Information items (e.g. Supplementary Figures, Supplementary Tables, Supplementary Methods, Supplementary Notes, Supplementary Discussion, Supplementary References) must be included in one PDF document. Please refer to our formatting guide when preparing your supplementary information file: https://www.nature.com/documents/ncomms-formatting-instructions.pdf  All Supplementary Information files (e.g. Supplementary Data, Supplementary Software, etc.) must be cited in the main text.  Every Supplementary Figure must be accompanied by a legend of up to 350 words, referring to all panels, and a brief title that summarises the whole figure.  Only Supplementary Movie, Audio, Data and Software files should be submitted separately from the Supplementary Information. | We have checked that all supplementary information items are included in one PDF document. Additionally, we have checked that all supplementary information files are cited in the main text.  We have added the following sentence in Caption of Figure 4 and 5:  The full process of the example is provided in Supplementary Note S1 and Supplementary Data 1.  The full process of the example is provided in Supplementary Data 1.  We have added the following sentence in Caption of Figure 7:  The data used to plot the violin is provided in Supplementary Data 2. |
| Please include a PDF file titled "Description of Additional Supplementary Files” with legends for each Supplementary Movie/Audio/Data file. Please label each file as Supplementary Movie/Audio/Data 1, etc. please format the legends as follows: | We have added the description of additional supplement files that contain the description of supplementary data files. |
| The use or adaptation of previously published images is strongly discouraged. If this is unavoidable, please request the necessary rights documentation to re-use such material from the relevant copyright holders and return this to us when you submit your revised manuscript. Please check whether your manuscript or Supplementary Information contain third-party images, such as figures from the literature, stock photos, clip art or commercial satellite and map data.  For more information on what constitutes ownership by a third party, please contact our Editorial Assistant at [naturecommunications@nature.com](mailto:naturecommunications@nature.com) | We have check images from third-party illustrations, each of which is copyrighted for user to modify and use. (commercial use or free for non-commercial use) |
| Please check in particular: | Free for commercial use:  <https://www.iconfinder.com/icons/4417114/desktop_pc_server_icon>  <https://www.iconfinder.com/icons/8008249/toolbox_container_repair_box_tool_box_toolboxes_toolkit_tools_icon>  <https://www.iconfinder.com/icons/403019/avatar_male_man_person_user_young_icon?coming-from=related-results>  https://www.iconfinder.com/icons/403023/avatar_user_woman_female_person_young_icon  Basic license: |
| **Supplementary Figure 11, There is suspected third party content in Figure 1,2.Kindly look into it.** | <https://www.iconfinder.com/icons/2754718/future_head_robot_robotics_tech_technology_icon>  https://www.iconfinder.com/icons/2305746/glass_lookup_magnifying_table_icon  https://www.iconfinder.com/icons/2757666/bulb_idea_lamp_light_icon |
|  |  |
| **Forms to complete** |  |
| Our guidance: | Your response: |
| **Editorial Policy Checklist** |  |
| Please update and upload a final version of the Editorial Policy Checklist with your revised manuscript files. A blank Editorial Policy Checklist can be found via the link below. Note that this form is a dynamic ‘smart pdf’ and must be downloaded and completed in Adobe Reader. | We have uploaded the final version of the editorial policy checklist. |
| Please update your current checklist or download from: |  |
| <https://www.nature.com/documents/nr-editorial-policy-checklist.zip> |  |
|  |  |
|  |  |
|  |  |
|  |  |
| 1. Please upload your main figures as individual files in ONE of these acceptable file formats: .ai, .eps, .pdf, .ps rather than combining them into one file. | We have divided the figure files into individual .ai format. |
| 2. Please ensure your main manuscript file includes the following sections, in this order, no other sections are permitted:  Title  Author list  Affiliations  Abstract  Introduction  Results  Discussion (optional)  Results and Discussion (optional)  Methods  Data Availability  Code Availability (if relevant)  References  Acknowledgements  Author Contributions Statement  Competing Interests Statement  Tables  Figure Legends/Captions (for main text figures | We have changed the order to match the guidelines. |
| 3. Since we are a commercial publisher, you may need to find an alternative to the image used below:  Free for non-commercial use: https://www.iconfinder.com/icons/49597/ordering_table_icon  4. Where you have used the image below (in Fig 1), please attribute/give credit within the appropriate figure legend according to the license.  Creative Commons (Attribution 3.0 Unported)  https://www.iconfinder.com/icons/653262/bulb_light_electric_energy_idea_lamp_thought_icon | We have changed the non-commercial and creative common 3.0 icons to the basic license icon in Figure 1(b).  - Bulb: <https://www.iconfinder.com/icons/2757666/bulb_idea_lamp_light_icon>  -Table: https://www.iconfinder.com/icons/2305746/glass_lookup_magnifying_table_icon |
| 5. Please provide your Supplementary Information as a .pdf source file. | We have provided the supplementary information as a .pdf source file. |
| 6. Thank you for your modifications on the abstract - however, we must ask you to please write the bits about your research in present tense rather than present perfect. | We have modified the following sentences into present tense.  "ChatMOF shows impressive accuracy rates of 96.9% for searching, 95.7% for predicting, and 87.5% for generating tasks with GPT-4."  " Additionally, it successfully creates materials with user-desired properties from natural language." |
| 7. We notice that you provide the raw data from Figure 7 as Supplementary data. However there are sets of chat-pgt3 and chat-pgt4 results in that file. Please clarify in the Figure legend which model was used. Additionally, we ask you to provide the exact data used to make those figures as a Source Data (not Supplementary Data) file. | We have added the following sentences in Caption for Figure 7,  “Tested on October 13, 2023 at 14:26 KST with GPT-4.”  Furthermore, we have changes Supplementary Data 2 to Source Data1 |
|  |  |
|  |  |
|  |  |
| **You will need to upload:** |  |
| Editorial Policy Checklist | O |
| Completed Third Party Rights Table (if relevant) |  |
| A point-by-point response to the reviewers' comments | O |
| A completed copy of this checklist | O |
| The main manuscript file in either Microsoft Word or LaTeX format | O |
| Separate Figure files | O |
| Inventory of Supporting Information | O |
| A Supplementary Information file | O |
| Reporting Summary | O |
|  |  |
